# RAF isoform selectivity of MEK inhibitors and rational design of a covalent ARAF-MEK inhibitor

**DOI:** 10.1126/sciadv.aee5241

**Published:** 2026-10-23

**Authors:** Sayan Chakraborty, Jie Jiang, Javier Vinals Camallonga, Dimitris Gazgalis, Thomas W. Gero, Maurício T. Tavares, Georgia Dittemore, Emre Tkacik, Tyler S. Beyett, Dong Man Jang, Byung Hak Ha, Felix H. Gottlieb, Talya Levitz, Rebecca J. Metivier, Anna M. Schmoker, David A. Scott, Hyesung Jeon, Pasi A. Jänne, Michael J. Eck

**Affiliations:** ^1^ Department of Cancer Biology, Dana-Farber Cancer Institute, Boston, MA 02215, USA.; ^2^ Department of Biological Chemistry and Molecular Pharmacology, Harvard Medical School, Boston, MA 02115, USA.; ^3^ Department of Medical Oncology, Dana-Farber Cancer Institute, Boston, MA 02215, USA.; ^4^ Department of Medicine, Harvard Medical School, Boston, MA 02115, USA.; ^5^ Department of Chemistry and Chemical Biology, Harvard University, Cambridge, MA 02138, USA.

## Abstract

Aberrant activation of the RAS/RAF/mitogen-activated protein kinase kinase (MEK)/extracellular signal–regulated kinase pathway is a frequent cause of cancer. Allosteric MEK inhibitors block this pathway by binding RAF-MEK complexes to prevent activation of MEK by RAF. However, how MEK inhibitor potency varies across the three RAF isoforms remains poorly understood. We profiled seven allosteric MEK inhibitors and observed a notable hierarchy of sensitivity: All most potently inhibited CRAF-driven MEK activation while relatively sparing ARAF-driven activation. We identified point mutations in ARAF and CRAF proximate to the MEK inhibitor binding site that markedly altered inhibitor sensitivity. Using a rational design approach, we developed a more potent inhibitor of ARAF-driven MEK signaling, TWG-07-148. Our cryo–electron microscopy structure shows how this acrylamide-containing analog of MEK inhibitor trametinib covalently targets Cys^514^, a residue unique to ARAF. Our studies highlight the importance of the activating RAF isoform as a determinant of MEK inhibitor sensitivity and provide proof of concept for the development of MEK inhibitors that more effectively block ARAF-driven MEK signaling via covalent targeting of Cys^514^.

## INTRODUCTION

Mitogen-activated protein (MAP) kinase kinase (MEK) is the central kinase in the three-tiered RAF/MEK/extracellular signal–regulated kinase (ERK) (MAP kinase) cascade (*1*–*3*). This signaling axis is normally triggered in response to activation of receptor tyrosine kinases on the cell surface, leading to activation of RAS, which, in turn, engages and activates RAF, the kinase at the top of this cascade (*2*, *4*, *5*). Physiologic activation of the pathway results in regulated changes in cell proliferation, differentiation, and survival, but mutations in components of this pathway can lead to its uncontrolled activation and are a frequent cause of cancer (*2*, *3*, *6*, *7*). RAS is the most frequently mutated oncogenic driver (*8*–*10*). Mutations in RAS are estimated to occur in 19% of all cancers (*10*, *11*), and mutations in BRAF, one of three RAF isoforms, drive ∼7% of all cancers (*12*). By far, the most common activating mutation in BRAF is the V600E point mutation (*13*), which accounts for approximately half of all malignant melanoma (*14*) and as much as 60% (*15*) of papillary thyroid carcinoma and is also a less frequent driver of lung, colorectal, and other cancers (*12*, *16*).

MEK inhibitors are a valuable therapeutic for treatment of certain tumors arising from aberrant activation of this pathway (*17*, *18*). MEK inhibitors trametinib, cobimetinib, and binimetinib are used in combination with RAF inhibitors dabrafenib, vemurafenib, and encorafenib, respectively, to treat BRAF^V600E+^ melanomas (*19*–*23*). Selected RAF and MEK inhibitor combinations are also approved for treatment of other solid tumors driven by BRAF^V600E^ (*20*, *22*, *24*, *25*). MEK inhibitors selumetinib and mirdametinib are used to treat plexiform neurofibromas in patients with type I neurofibromatosis (*26*), a condition caused by loss-of-function mutations in NF1, a guanosine triphosphatase–activating protein that down-regulates RAS.

These and other clinical stage MEK inhibitors differ from most kinase inhibitors in that they have an allosteric mechanism of action (*27*, *28*). Rather than blocking binding of adenosine 5′-triphosphate (ATP) and directly inhibiting the ability of MEK to phosphorylate ERK, they bind in an allosteric pocket where they stabilize a conformation of the MEK activation segment that interferes with the ability of RAFs to phosphorylate and activate MEK (*28*). This allosteric mechanism of action has been recognized almost from the time of the discovery of the first MEK inhibitors in the mid-1990s (*27*), and its general structural basis became clear with the first cocrystal structures in 2004 (*28*). More recently, structural studies of MEK inhibitors bound to RAF-MEK complexes and with MEK in complex with kinase suppressor of Ras (KSR; a RAF-like pseudokinase) have revealed that the MEK inhibitor binding pocket is formed, in part, by the juxtaposed RAF kinase and that MEK inhibitors can differentially stabilize these complexes (*29*–*32*).

Owing to their allosteric mechanism, the pharmacologic effects of MEK inhibitors can be idiosyncratic. In particular, they can vary in their efficacy depending on how the pathway is activated. For example, cobimetinib and mirdametinib are effective in blocking MAP kinase signaling in the context of BRAF^V600E^, but not mutant KRAS, while MEK inhibitors GDC-0623 and trametinib are effective in both oncogenic contexts (*33*, *34*). Activation of CRAF in KRAS mutant cells was identified as a determinant of sensitivity to mirdametinib—MEK was found to be less susceptible to inhibition when activated by CRAF as compared with BRAF^V600E^ (*34*). While MEK inhibitors act most potently on RAF-MEK complexes to block MEK activation, at higher concentrations, most can inhibit active, phosphorylated MEK (*29*, *35*). This property may explain, in part, the differing ability of MEK inhibitors to block ERK activation in KRAS mutant cells (*29*, *35*). One exception is avutometinib (CH5126766), which is known to selectively target RAF-MEK complexes and to have essentially no ability to inhibit free, active MEK (*29*, *36*).

Although MEK inhibitors have long been known to target RAF-MEK complexes, to our knowledge, their activity against complexes containing different RAF isoforms has not been systematically investigated. This question has important therapeutic implications. Although rare in comparison with BRAF mutations, activating mutations in both ARAF and CRAF have also been identified as oncogenic drivers (*37*–*41*). In addition, RAF isoforms can compensate for one another during targeted therapy and can mediate resistance to BRAF and MEK inhibitors that arises via relief of negative feedback inhibition (*42*, *43*). In this study, we systematically profiled seven allosteric MEK inhibitors across RAF isoforms using both biochemical and cell-based assays. We found marked differences in isoform selectivity with most MEK inhibitors exhibiting relative sparing of ARAF-driven MEK signaling. Through structure-guided mutagenesis, we identified RAF residues in the proximity of the MEK inhibitor binding pocket—Gly^513^ (G513) and Cys^514^ (C514) in ARAF and Asn^553^ (N553) in CRAF—as critical determinants of sensitivity to MEK inhibitors. Recognizing the proximity of Cys^514^ in ARAF to the MEK inhibitor binding site, we explored covalent targeting of this residue leading to the development of TWG-07-148, an acrylamide-containing analog of trametinib. TWG-07-148 binds irreversibly to Cys^514^ on ARAF and more potently inhibits ARAF-MEK signaling as compared with trametinib and other existing MEK inhibitors. Collectively, our studies reveal a hierarchy of RAF isoform sensitivity to current MEK inhibitors, with CRAF>BRAF>ARAF, and provide a strategy for more potent inhibition of the ARAF-driven MEK activation via covalent targeting of a cysteine residue that is unique to ARAF.

## RESULTS

### MEK inhibitors differ in potency across RAF isoforms

To test MEK inhibitor potency in an isogenic context, we prepared Ba/F3 cell lines that stably express activated alleles of ARAF, BRAF, or CRAF [ARAF^Ser214Ala (S214A)^, BRAF^Ser365Ala (S365A)^, and CRAF^Ser259Ala (S259A)^]. These mutations ablate the negative-regulatory phosphorylation and 14-3-3 binding site in each RAF and all promoted interleukin-3 (IL-3)–independent proliferation of the respective Ba/F3 cell line. We also prepared Ba/F3 cells transformed by BRAF^Val600Glu (V600E)^. We tested a panel of seven MEK inhibitors on proliferation of each cell line and on the parental Ba/F3 cells using the CellTiter-Glo (Promega) cell viability assay (Fig. 1A and fig. S1, A and B). Although Ba/F3 cells endogenously express low levels of wild-type (WT) RAFs, all were sensitized to MEK inhibition as compared with the parental Ba/F3 cells, further confirming their dependence on the activated RAF alleles. While we cannot exclude the possibility of heterodimerization of the exogenously expressed activated RAF isoforms with endogenous WT RAFs, we expect this to be negligible due to the relative abundance of the exogenously expressed RAFs and because the WT RAFs should be maintained in an autoinhibited conformation that is resistant to dimer formation.

**Fig. 1.**
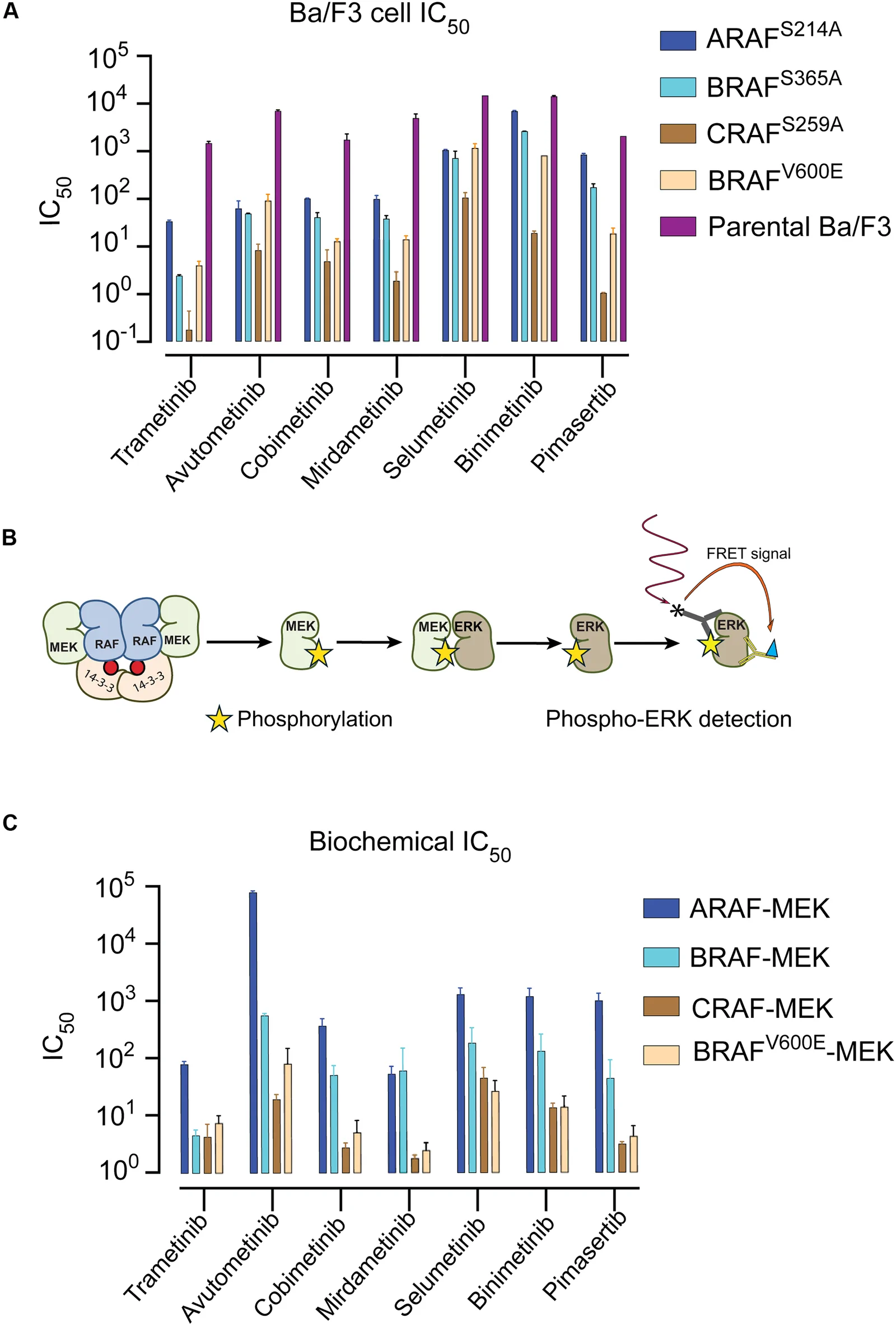
Potency and selectivity of allosteric MEK inhibitors across RAF isoforms. (**A**) Bar chart showing the cellular median inhibitory concentration (IC_50_) values of allosteric MEK inhibitors in Ba/F3 cells expressing activated alleles of ARAF, BRAF, or CRAF. The parental Ba/F3 cell line lacking exogenous RAF expression was included as a control. IC_50_ values for inhibition of proliferation are reported as mean; *N* = 2 with ± SEM indicated by error bars. (**B**) Schematic of the cascade biochemical assay used to measure ERK phosphorylation in the presence or absence of MEK inhibitors. (**C**) Bar chart summarizing the biochemical potency of MEK inhibitors against purified active RAF-MEK complexes, reported as mean of *N* = 3 with SEM indicated by error bars.

All seven of the MEK inhibitors tested were most potent on the CRAF-driven Ba/F3 cells and least potent on ARAF-driven cells (Fig. 1A, fig. S1B, and table S1). While the difference in potency on CRAF versus ARAF varied, it was typically on the order of 10- to 100-fold. Among the MEK inhibitors we tested, trametinib was the most uniformly potent across RAF isoforms, with a subnanomolar median inhibitory concentration (IC_50_) against CRAF^S259A^ and an IC_50_ of ∼29 nM against ARAF^S214A^. The tested MEK inhibitors exhibited intermediate potency on BRAF^S365A^, with measured IC_50_ values ranging from 3 nM for trametinib to ∼700 nM for selumetinib. Potencies on BRAF^S365A^ and BRAF^V600E^ were very similar, indicating that the two different mechanisms of activation do not differentially affect MEK inhibition (Fig. 1A, fig. S1B, and table S1).

We also compared potencies of MEK inhibitors in biochemical assays against purified ARAF-MEK1, BRAF-MEK1, and CRAF-MEK1 complexes. These preparations consist of active, 14-3-3–bound dimers of the respective RAF kinase domains, all prepared as complexes with MEK1 (*44*). For ARAF and CRAF, we incorporated an “SSDD” substitution in their N-terminal acidic motifs (NtA motifs; replacing their native SSYY and SGYY sequences, respectively), which has previously been shown to increase their catalytic activity in lieu of tyrosine phosphorylation of their native NtA motifs (*45*). In this cascade assay, phosphorylation of ERK2 is measured using a TR–fluorescence resonance energy transfer (FRET)–based sandwich immunoassay that uses a phosphospecific antibody for phospho-ERK1/2 (pERK1/2; Thr^202^/Tyr^204^) (Fig. 1B). Relative potencies in these biochemical assays largely paralleled those in our Ba/F3 assays—all seven MEK inhibitors were most potent against the CRAF-MEK complex and least potent against the ARAF-MEK complex (Fig. 1C, fig. S1C, and table S2). As in the cellular assays, differences in potency on ARAF versus CRAF were on the order of 10- to 100-fold. One difference, however, was that most of the tested MEK inhibitors were considerably more potent against BRAF^V600E^-MEK than against the WT BRAF-MEK complex in the biochemical assay. This difference could arise from our use of a monomeric preparation of BRAF^V600E^ that lacked the 14-3-3–stabilized dimerization of the BRAF^V600E^-MEK complex. Overall, our findings in both the cellular and biochemical assays demonstrate that all seven MEK inhibitors tested are most potent against CRAF-driven MEK activation and least potent against ARAF-driven MEK activation. While this rank-order potency was the same irrespective of the MEK inhibitor chemotype, the extent of the RAF isoform selectivity did vary markedly among the inhibitors tested (tables S1 and S2).

### Mutations in the RAF α_G_ helix can confer sensitivity or resistance to MEK inhibition

Crystal structures of RAF-MEK complexes show that the MEK inhibitor binding cleft is bordered on one end by three residues at the N terminus of the α_G_ helix in RAF (Fig. 2A) (*29*). Both trametinib and avutometinib make direct contacts with Asn^661^ in this segment in BRAF-MEK complexes, and mutations in this region have been shown to affect the ability of trametinib to stabilize RAF-MEK and KSR-MEK complexes (*29*, *30*). Sequence comparisons reveal that the Asn-Asn-Arg (NNR) sequence conserved in BRAF and CRAF is replaced by Gly-Cys-Arg (GCR) in ARAF (Fig. 2B), and given the proximity of these residues to the MEK inhibitors binding site and their divergence in ARAF, we were interested to test the effect of mutations in this region on the potency of MEK inhibitors.

**Fig. 2.**
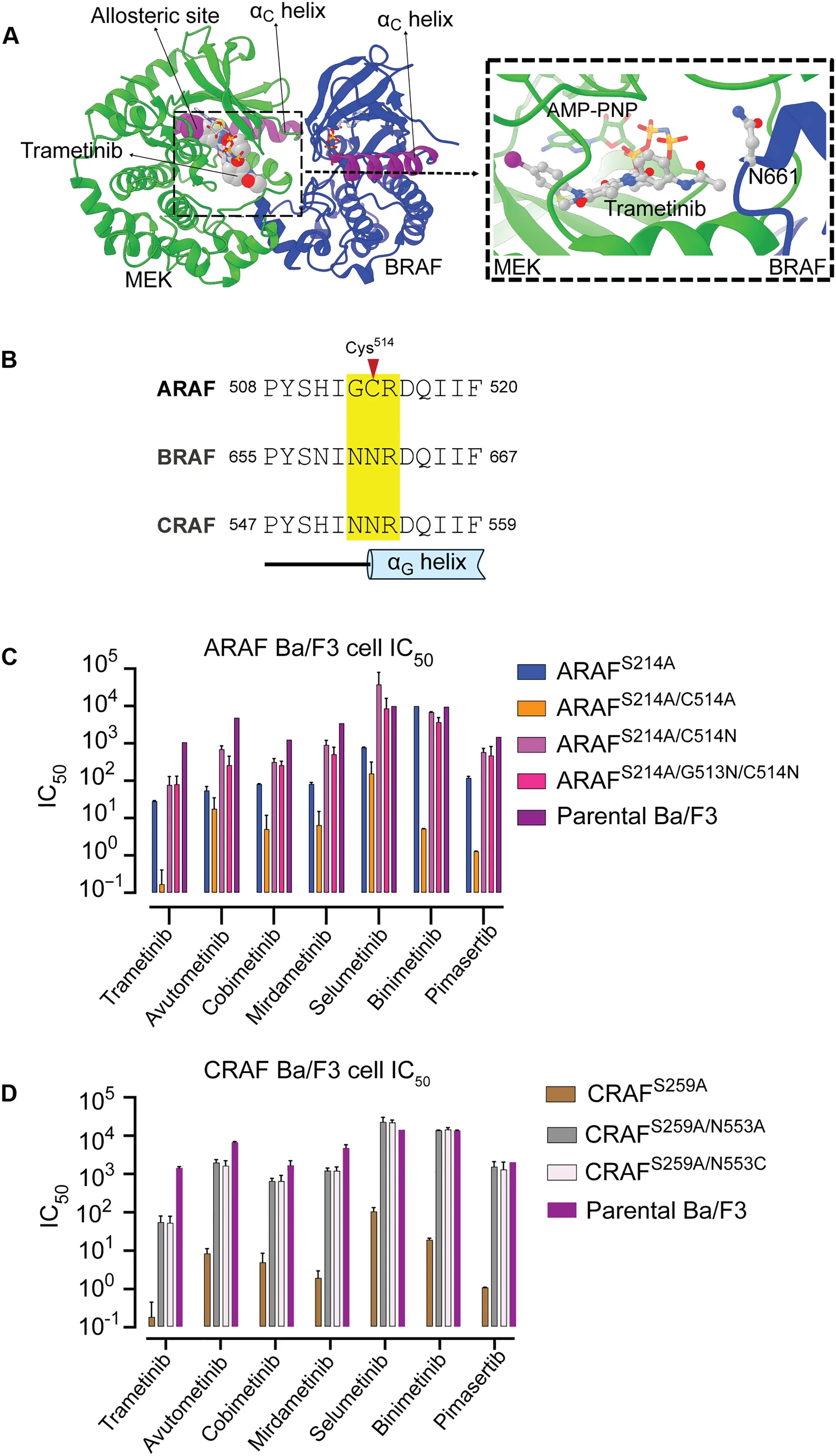
Impact of ARAF and CRAF mutations on MEK inhibitor sensitivity. (**A**) Ribbon diagram depicting the structure of the BRAF-MEK complex, with trametinib bound to the allosteric site of MEK [Protein Data Bank (PDB) entry 7M0Y]. The inset shows a detailed view of trametinib and its relationship to Asn^661^ in the BRAF α_G_ helix. (**B**) Aligned sequences of human ARAF, BRAF, and CRAF in the region of the RAF-MEK allosteric pocket. Residues that form the RAF portion of the pocket are highlighted in yellow, and the position of Cys^514^ in ARAF is marked with an arrow. (**C** and **D**) Bar charts showing the cellular IC_50_ values of allosteric MEK inhibitors in Ba/F3 cells expressing individual ARAF (C) or CRAF (D) mutants, as indicated. The parental Ba/F3 cell line lacking exogenous RAF expression was included as a control.

We mutated Cys^514^ in ARAF (corresponding to Asn^661^ in BRAF) to alanine and asparagine, and we also prepared the ARAF Gly^513^Asn/Cys^514^Asn (G513N/C514N) double mutant to match the NNR motif found in BRAF and CRAF. These mutations were introduced into the activated ARAF^S214A^ construct in Ba/F3 cells. The Cys^514^Ala (C514A) mutation markedly sensitized ARAF to MEK inhibition. All seven MEK inhibitors were more potent against this mutant, with IC_50_ shifts ranging from ∼250-fold for trametinib to ∼8-fold for avutometinib (Fig. 2C, fig. S2A, and table S3). By contrast, the single and double asparagine mutations increased IC_50_ values for the panel of MEK inhibitors (Fig. 2C, fig. S2A, and table S3). These large shifts in MEK inhibitors sensitivity were not recapitulated in our biochemical assay, although the C514A mutant did modestly sensitize to the tested MEK inhibitors (fig. S2C and table S4).

To better understand the underlying basis for these changes in inhibitor sensitivity, we carried out molecular dynamics simulations with the WT ARAF-MEK1 complex and with the Cys^514^Ala (C514A) and Cys^514^Asn (C514N) mutants. In all trajectories, we observed coupled movements between the ARAF α_G_ helix and the MEK1 activation segment helix. With both mutants, we observed closer contact between these structural elements and also between the α_G_ helix and the bound inhibitor (fig. S3, A to C). Calculated protein-ligand interaction energies predicted higher affinity for the trametinib scaffold in both mutants as compared with WT (fig. S3D). These simulations support the notion that changes in the dynamics of the interface affect inhibitor affinity, and calculated changes in ligand interaction energies are consistent with observed changes in our biochemical, but not cellular, assays.

We also examined the effect of cysteine and alanine mutations in the corresponding residue of CRAF Asn^553^Ala and Asn^553^Cys (N553A and N553C) in our Ba/F3 cell model. Both substitutions resulted in profound resistance to MEK inhibition, resulting in sensitivity similar to that of the parental Ba/F3 cells (Fig. 2D, fig. S2B, and table S3). Collectively, these findings reinforce the importance of the RAF partner in RAF-MEK complexes as a determinant of sensitivity to the tested MEK inhibitors.

### A more potent ARAF-MEK inhibitor via covalent targeting of ARAF^Cys514^

Although ARAF itself is only rarely mutated in cancer (*12*, *39*, *46*), it is increasingly appreciated as an important therapeutic target owing to its role in mediating resistance to RAF inhibitors, particularly in NRAS-mutant cancers (*40*, *47*). As we find above, most MEK inhibitors are least effective in the context of ARAF-driven MEK activation, and furthermore, current RAF inhibitors, particularly type II inhibitors, are also least effective against ARAF (*44*, *48*). Motivated by this need and encouraged by the position of ARAF Cys^514^ in the MEK inhibitor binding pocket, we sought to develop a more potent inhibitor of ARAF-MEK via covalent targeting of this residue.

In our rational design approach, we appended covalent warheads (acrylamide or chloroacetamide) to MEK inhibitor scaffolds in a manner expected to juxtapose the warheads with the target cysteine residue in ARAF. We synthesized a series of 11 compounds based on the core scaffolds of trametinib, avutometinib, cobimetinib, and selumetinib and assessed their ability to form the desired covalent bond with ARAF and their potency against activated ARAF^S214A^ in the Ba/F3 cell assay (Fig. 3 and Synthesis and characterization for all 11 target compounds in the Supplementary Materials). Selected acrylamide and chloroacetamide derivatives of both trametinib and avutometinib stoichiometrically labeled ARAF when applied to the ARAF-MEK complex (Fig. 3). Intact mass analysis suggested a single site of labeling at ∼100% occupancy for both trametinib-based TWG-07-148 and avutometinib-based GD-01-012 (Fig. 4A), and mapping of the sites by liquid chromatography–tandem mass spectrometry (LC-MS/MS) analysis of a tryptic digest of the labeled samples confirmed the site of labeling as Cys^514^ in ARAF, the target residue (Fig. 4B). The trametinib-based compounds were potent in the ARAF-driven Ba/F3 assay, with TWG-07-148 exhibiting an IC_50_ = 5.5 nM, ∼5-fold more potent than trametinib (Fig. 4C and table S1). TWG-07-148 was also more potent than trametinib in the biochemical ARAF-MEK assay (Fig. 4D).

**Fig. 3.**
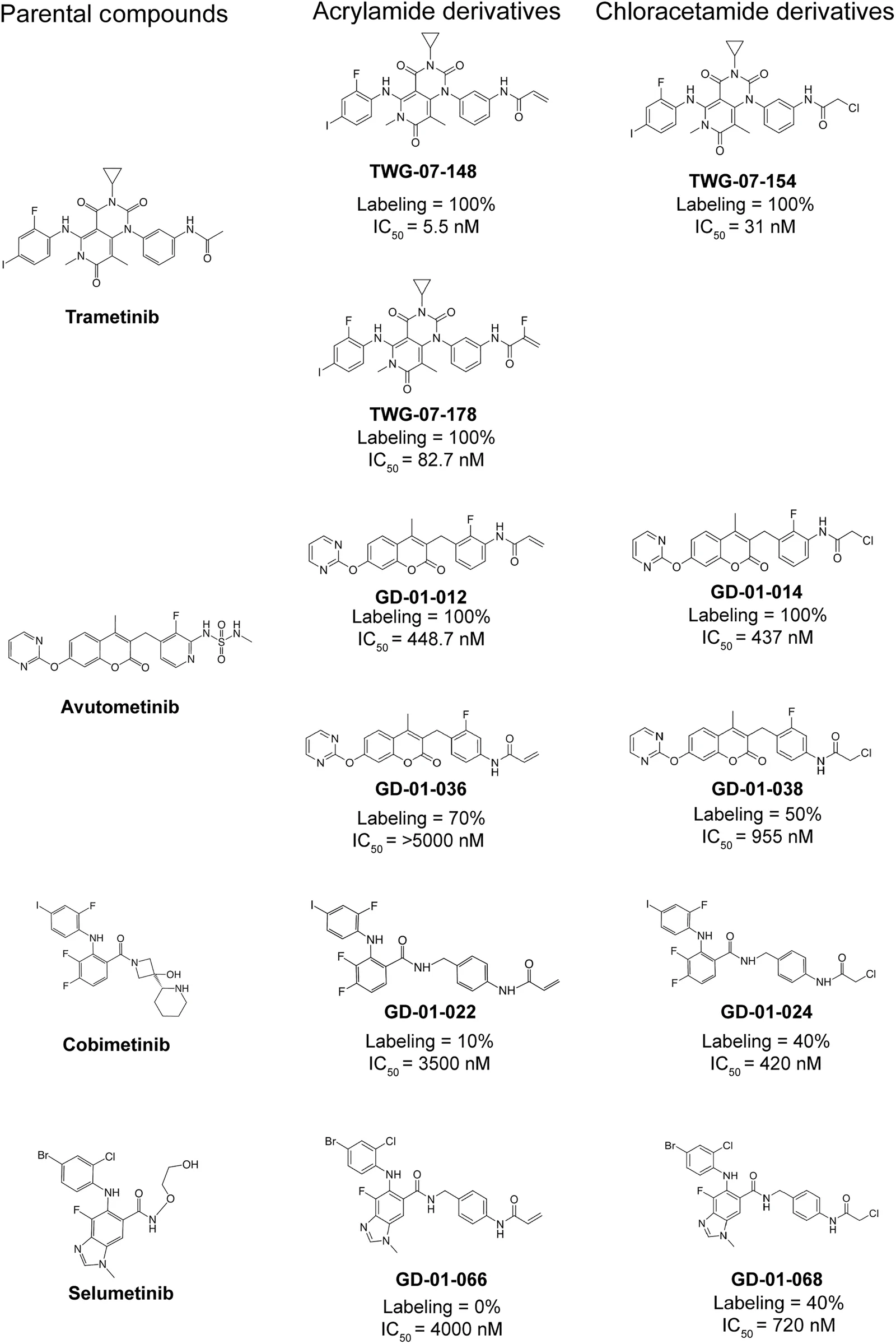
Structures of covalent MEK inhibitors targeting ARAF Cys^514^. Electrophilic acrylamide or chloroacetamide groups were introduced in existing allosteric MEK inhibitor scaffolds (those of trametinib, avutometinib, cobimetinib, and selumetinib) with the goal of covalent targeting of Cys^514^ in ARAF. For each of the resulting compounds, covalent labeling of ARAF was measured in an intact MS assay with the ARAF-MEK complex (labeling %). IC_50_ values provided for each compound are for inhibition of proliferation of Ba/F3 cells expressing ARAF^S214A^.

**Fig. 4.**
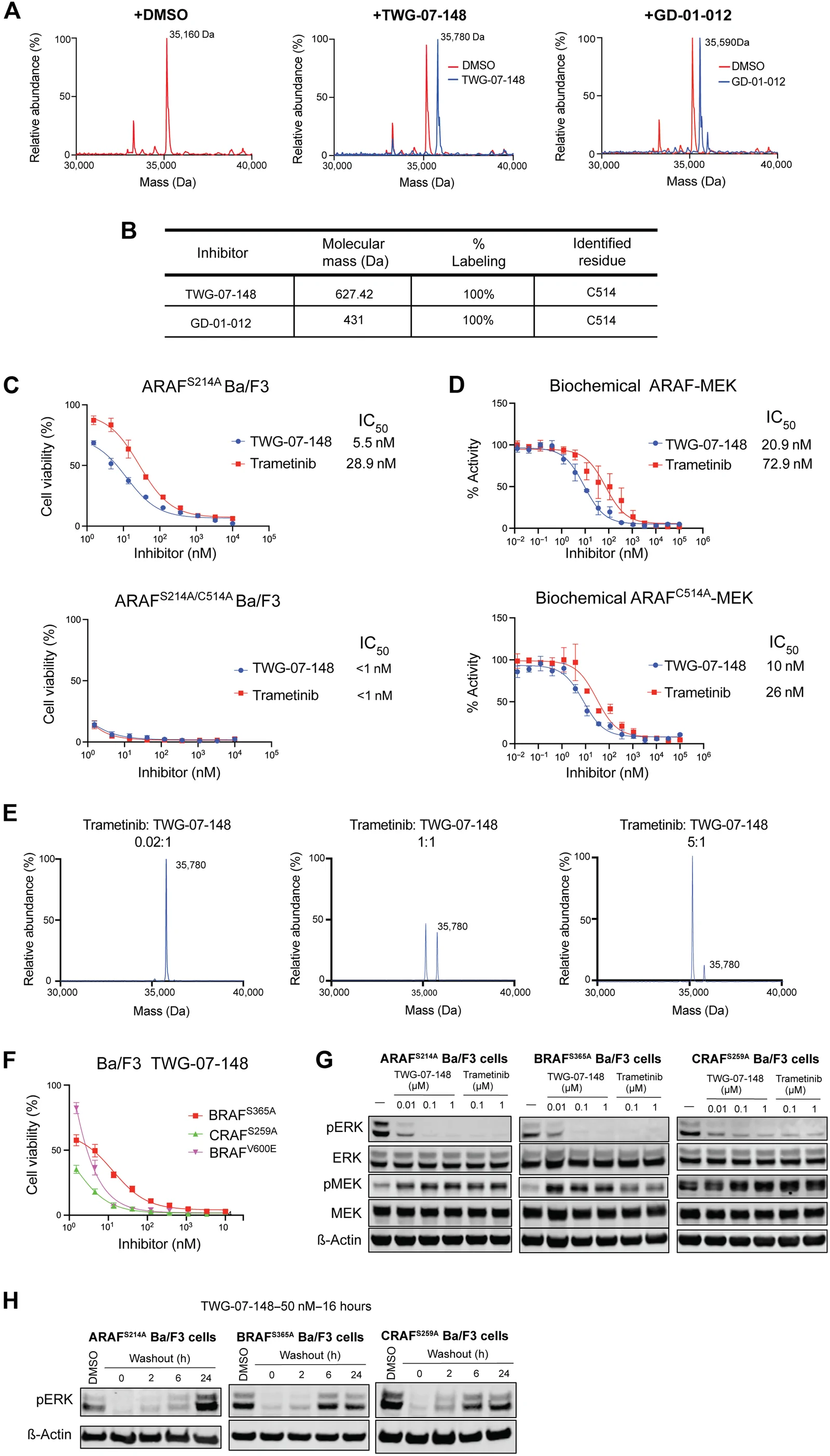
Biochemical and cellular evaluation of covalent ARAF-MEK inhibitor TWG-07-148. (**A**) Intact MS analysis of the ARAF-MEK1 complex after treatment with trametinib derivative TWG-07-148 (center), avutometinib derivative GD-01-012 (right), or dimethyl sulfoxide (DMSO) control (left). The DMSO control is superimposed in red for reference in the TWG-07-148 and GD-01-012 panels. MEK1 lies outside the mass range illustrated. (**B**) LC-MS/MS analysis of a tryptic digest of the TWG-07-148– and GD-01-012–treated samples identified Cys^514^ as the site of covalent modification. (**C**) Dose-response curves for TWG-07-148 and trametinib in Ba/F3 cells expressing ARAF^S214A^ (top) or ARAF^S214A/C514A^ (bottom). (**D**) Dose-response curves for TWG-07-148 and trametinib in the biochemical cascade assay with ARAF-MEK1 (top) or ARAF^C514A^-MEK1 (bottom). (**E**) MS-based competition assay with trametinib and TWG-07-148. Increasing concentrations of the reversible MEK inhibitor trametinib compete for the same pocket as TWG-07-148 to block labeling of ARAF Cys^514^, supporting MEK-mediated delivery of the covalent compound to Cys^514^ of ARAF. The ARAF-MEK1 complex (3 μM) was treated with an equimolar concentration of TWG-07-148 plus trametinib at 0.06 μM (0.02:1), 3 μM (1:1), or 15 μM (5:1) and analyzed by intact MS. (**F**) Dose-response curves of TWG-07-148 against Ba/F3 cell lines expressing BRAF^S365A^, CRAF^S259A^, and BRAF^V600E^. (**G**) Immunoblot analysis of Ba/F3 cells demonstrating inhibition of pERK by TWG-07-148 compared with trametinib. Trametinib is known to allow single-site phosphorylation of MEK on Ser^222^, and the single-phosphorylated MEK is recognized by most commercial anti–phospho-MEK (pMEK) S217/221 antibodies (*29*). (**H**) Immunoblot analysis showing prolonged inhibition of ERK phosphorylation in ARAF^S214A^ Ba/F3 cells compared with BRAF^S365A^ and CRAF^S259A^ Ba/F3 cell lines upon TWG-07-148 treatment. Ba/F3 cell lysates were collected at 0, 2, 6, and 24 hours after washout following treatment with DMSO or TWG-07-148 (50 nM) for 16 hours. Blots are representative of three independent experiments. h, hours.

Because of the idiosyncratic increase in sensitivity of the ARAF^C514A^ to reversible inhibition with MEK inhibitors, this mutation was not useful for assessing the role of covalent bond formation in the increased potency of TWG-07-148 as compared with trametinib (Fig. 4, C and D, bottom). However, in support of the importance of a covalent contribution to its potency, we note that replacement of the acrylamide in TWG-07-148 with the less reactive fluoroacrylamide warhead in TWG-07-178 resulted in a significant decrease in potency against ARAF^S214A^ (IC_50_ = 82.7 nM for TWG-07-178 versus 5.5 nM for TWG-07-148; Fig. 3).

Although avutometinib derivatives GD-01-012 (acrylamide) and GD-01-014 (chloroacetamide warhead) efficiently labeled ARAF, they were less potent than the parent compound in the Ba/F3 cell assay, with IC_50_ values of ∼450 nM (Fig. 3). We did not observe efficient labeling with any of the cobimetinib or selumetinib derivatives, and these were not studied further. We therefore focused our further characterization on trametinib analog TWG-07-148, the only covalent compound that was demonstrably more potent than its reversible parent.

To determine whether covalent labeling of ARAF was MEK dependent, i.e., whether engagement of the compound in the MEK allosteric pocket “delivered” the warhead to Cys^514^, we carried out a competition experiment with trametinib. In support of the intended mechanism of action, the presence of a 1:1 or 5:1 ratio of trametinib to TWG-07-148 proportionately blocked labeling of ARAF by TWG-07-148 when the ARAF-MEK complex was incubated with both compounds (Fig. 4E). We also profiled TWG-07-148 on our Ba/F3 cell models driven by BRAF^S365A^, CRAF^S259A^, and BRAF^V600E^ (Fig. 4F and table S1). Measured potencies in the BRAF and CRAF cell lines closely paralleled those of trametinib; the enhanced potency of TWG-07-148 was unique to ARAF, which contains the target cysteine residue. Western blotting for phospho-MEK (pMEK) and pERK in these cell lines confirmed dose-dependent suppression of MAP kinase pathway signaling by TWG-07-148, comparable to that observed with trametinib (Fig. 4G). In a Ba/F3 cell–based washout experiment, TWG-07-148 demonstrated prolonged inhibition of ERK phosphorylation in ARAF-driven cell line compared to BRAF- and CRAF-expressing cell lines (Fig. 4H). These findings are consistent with our proposed mechanism of covalent targeting of ARAF, resulting in prolonged suppression of ARAF-MEK signaling.

Considering the exquisite selectivity of its trametinib scaffold for MEK (*28*, *29*), we expected TWG-07-148 to exhibit excellent selectivity across the kinome. This was confirmed by profiling in a 70-kinase diversity panel that used a radiometric biochemical assay (table S6) (*49*, *50*).

### Cryo–electron microscopy structure of an ARAF-MEK complex with TWG-07-148

To better understand the binding mode of TWG-07-148, we determined a cryo–electron microscopy (cryo-EM) structure of the ARAF kinase domain in complex with MEK1, TWG-07-148, and the RAF inhibitor GDC-0879 (see Materials and Methods, fig. S4, and table S5). GDC-0879 is a type I RAF inhibitor that promotes the active conformation of the kinase and, in turn, drives dimerization of the kinase domain (*51*). The protein complex was affinity purified with both GDC-0879 and TWG-07-148. Subsequent size exclusion chromatography (SEC), using an inhibitor-free buffer, yielded a sharp monodisperse peak (fig. S4A). SDS–polyacrylamide gel electrophoresis (SDS-PAGE) further confirmed the presence of pure ARAF-MEK1 complex (fig. S4B). This complex was used for cryo-EM imaging and single-particle reconstruction (fig. S4, A to G, and table S5). The nominal resolution of the cryo-EM map was 2.51 Å (fig. S4F and table S5).

The resulting structure revealed a twofold symmetric, heterotetrameric arrangement consisting of a back-to-back dimer of the ARAF kinase domain with MEK1 bound to each ARAF kinase (Fig. 5, A and B). To our knowledge, ARAF has not previously been resolved in its active state, but the dimer conformation and dimer interface are essentially the same as those observed in active BRAF and CRAF structures (Fig. 5C). This is not unexpected, considering that RAFs signal via formation of heterodimers and homodimers (*52*–*54*). The ARAF construct we used for structure determination incorporates the activating SSDD substitution of the NtA motif. However, as in available BRAF and CRAF structures, this region is not resolved in the ARAF cryo-EM density. Three-dimensional (3D) variability analysis of the reconstruction revealed a modest rocking motion of MEK1 with respect to the ARAF dimer (movie S1). This variability likely explains the moderately weaker density in the MEK1 portions of the cryo-EM map.

**Fig. 5.**
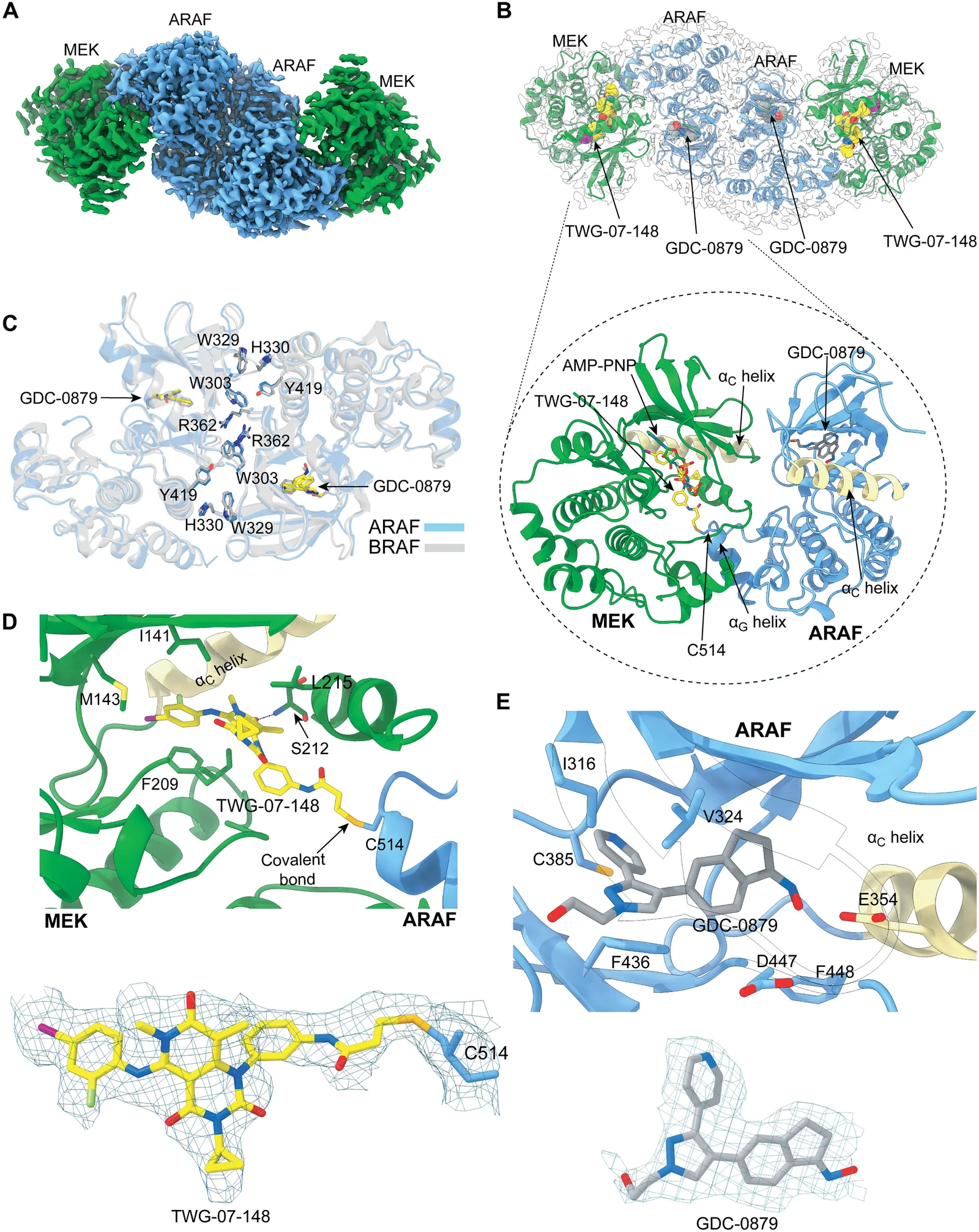
Cryo-EM structure of the ARAF-MEK1 heterotetramer bound to GDC-0879 and covalent MEK inhibitor TWG-07-148. (**A**) A 2.51-Å cryo-EM map of ARAF-MEK1 assembly reveals a heterotetrameric MEK1:ARAF:ARAF:MEK1 configuration. (**B**) Ribbon representation of the ARAF-MEK1 heterotetramer with the cryo-EM map shown as a transparent surface. GDC-0879 occupies the ARAF ATP binding pocket in both protomers, and TWG-07-148 occupies the MEK1-ARAF allosteric site. Adenylyl-imidodiphosphate (AMP-PNP) occupies the ATP site of both MEK1 protomers. The enlarged view highlights the ARAF-MEK interface. (**C**) Structural comparison of the ARAF back-to-back dimer interface in the present structure with that of active BRAF (from PDB entry 4MNF). Key dimer interface residues are identically conserved and are shown in blue for ARAF and gray for BRAF. Residue numbering corresponds to ARAF. (**D**) Detailed view of TWG-07-148 (yellow) in the allosteric pocket, where it forms a covalent bond with Cys^514^ in ARAF. Selected interacting residues are shown in stick form, and a hydrogen bond with the backbone amide of Ser^212^ is indicated by a dashed line. (**E**) Binding mode of GDC-0879 (gray colored) within the ARAF active site. Key interacting amino acids are highlighted, and hydrogen bonds are shown as dashed lines. Cryo-EM density in the region TWG-07-148 forming covalent bond with Cys^514^ of ARAF and GDC-0879 is shown in the bottom of (D) and (E), respectively.

Although the local resolution for the MEK1 portion of the structure, including the inhibitor binding pocket, was somewhat lower than for the structure as a whole (∼3 Å), the cryo-EM map revealed clear density for TWG-07-148 and confirmed formation of the covalent bond with Cys^514^ in ARAF (Fig. 5D). The overall binding pose of TWG-07-148 is essentially the same as that of the noncovalent parent compound trametinib. The 4-bromo-2-fluorophenyl group binds in the back of the MEK allosteric pocket where it engages in hydrophobic interactions with Ile^141^ and Met^143^, and the oxygen of central pyridopyrimidine ring system is positioned to hydrogen bond with the main chain amide of Ser^212^ in MEK (Fig. 5D). Contacts between ARAF and MEK in this region are closely similar to those seen in a recent structure of an inactive-state ARAF-MEK structure with NST-628 (*31*) and in other RAF-MEK complexes (*29*), indicating that covalent bond formation can be accomplished without notable main chain rearrangements in either ARAF or MEK.

The ARAF kinase domain exhibits a DFG-in, C helix–in conformation, consistent with the type I binding mode of GDC-0879. GDC-0879 binds in the ARAF ATP site in a manner analogous to that previously observed in BRAF (*55*). All residues that directly contact the compound are the same in ARAF and BRAF. The pyrazole core of GDC-0879 is sandwiched between Ile^316^ and Val^324^ in the P-loop and Phe^436^ in the C-lobe, and the pyridyl nitrogen hydrogen bonds with the backbone amide of Cys^385^ in the kinase hinge (Fig. 5E). The ketoxime oxygen is positioned to hydrogen bond with Glu^354^ in the C helix.

### TWG-07-148 inhibits proliferation of cancer cell lines driven by BRAF or KRAS mutations

We next evaluated the antiproliferative effects of TWG-07-148 on cancer cell lines harboring activating mutations in BRAF (HT29 and A375), KRAS (H2122, A549, and HCT116), or epidermal growth factor receptor (EGFR; PC9). TWG-07-148 potently inhibited proliferation of both BRAF and KRAS mutant cancer cell lines, with IC_50_ values ranging from 7 nM for A375 (BRAF^V600E^) to 48 nM in HCT116 (KRAS^G13D^; Fig. 6, A and B). The compound was somewhat less potent against PC9 cells (137 nM), which harbor an EGFR exon 19 deletion. We profiled established MEK inhibitors trametinib, avutometinib, cobimetinib, and selumetinib in the same cell lines (Fig. 6, A and B). Expectedly, the activity of TWG-07-148 most closely paralleled that of trametinib, with which it shares a common scaffold. In A549 cells, TWG-07-148 more comprehensively inhibited proliferation and was also modestly more potent as compared with trametinib. Similar to trametinib, TWG-07-148 inhibited phosphorylation of MEK and ERK in BRAF^V600E+^ A375 melanoma cell line and HT29 colorectal cell line (Fig. 6C). Substantial suppression of pMEK and pERK was observed at concentrations as low as 0.01 μM, demonstrating potent on-target inhibition of signaling through the MAP kinase pathway.

**Fig. 6.**
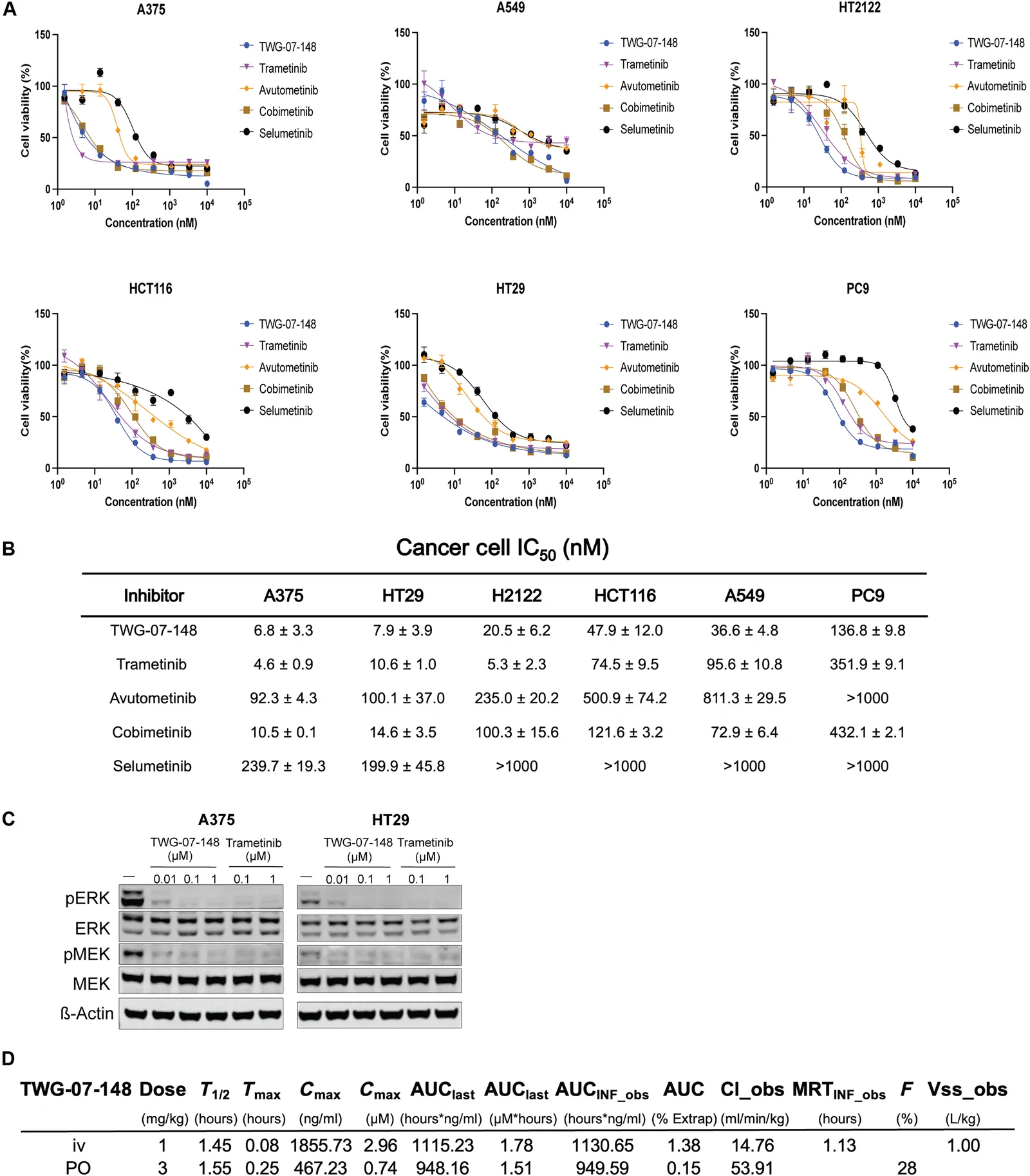
Profiling of TWG-07-148 and other allosteric MEK inhibitors in selected cancer cell lines. (**A**) Dose-response curves for TWG-07-148, trametinib, avutometinib, cobimetinib, and selumetinib in A375, HT29, H2122, HCT116, A549, and PC9 cells. Cells were treated for 72 hours, and cell viability was assessed using the CellTiter-Glo assay. (**B**) Table of IC_50_ values derived from the dose-response curves in (A). Data are presented as mean ± SEM (*N* = 3 or 4). (**C**) Immunoblot analysis showing inhibition of pMEK and pERK by TWG-07-148 and trametinib in A375 and HT29 cells. (**D**) Table showing the pharmacokinetic (PK) profile of TWG-07-148 in C57BL/6 male mice. Dosages were 1 mg/kg intravenously (iv) and 3 mg/kg PO. Data are represented as mean of three independent experiments.

Because MEK inhibitors have been shown to result in cell cycle arrest, we tested the effect of TWG-07-148 on cell cycle progression in both our RAF-mutant Ba/F3 cell lines and in BRAF^V600E+^ cancer cell lines. In ARAF^S214A^, BRAF^S365A^, and CRAF^S259A^ Ba/F3 cells, treatment with TWG-07-148 for 48 hours (at concentrations of 0.1 and 1 μM) significantly increased the fraction of cells in the G_0_/G_1_ phase, consistent with the expected effect of inhibiting the transition from G_1_ to the M phase (fig. S5, A and B). We observed a similar block when BRAF^V600E+^ A375 (melanoma) and HT29 (colorectal) cell lines were treated with TWG-07-148 or trametinib at the same concentrations (fig. S5, C and D).

We obtained mouse pharmacokinetic (PK) data for TWG-07-148 to assess its potential utility in future in vivo efficacy studies. Upon intravenous dosing, the compound showed low plasma clearance (15 ml/min per kilogram) and oral bioavailability of 28%, suggesting that good exposure levels could be achieved to deliver covalent binding in vivo (Fig. 6D).

## DISCUSSION

Our profiling of seven allosteric MEK inhibitors across RAF isoforms reveals a previously unrecognized hierarchy of sensitivity, with all tested compounds showing greatest potency against CRAF-driven MEK activation and substantially reduced activity against ARAF-driven signaling. This isoform-dependent variation in inhibitor efficacy spanned 10- to 100-fold differences in potency and likely reflects intrinsic properties of these distinct RAF-MEK complexes, as it was observed in both cellular and biochemical assays.

Through structure-guided mutagenesis, we identified mutations in the N terminus of the RAF α_G_ helix that alter MEK inhibitor sensitivity. The marked sensitization to MEK inhibition in the ARAF C514A mutant, coupled with the resistance conferred by introducing cysteine or alanine at the corresponding position in CRAF, demonstrates that sequence variations in regions bordering the allosteric binding pocket can profoundly influence inhibitor efficacy. In addition, these findings further highlight the importance of the RAF partner as a determinant of sensitivity to MEK inhibition.

The mechanistic basis for the differences in RAF isoform sensitivity is complex. Our mutagenesis experiments show that simply substituting the Gly-Cys-Arg sequence in the ARAF α_G_ helix with the Asn-Asn-Arg sequence found in CRAF does not confer comparable sensitivity to MEK inhibitors. Molecular dynamics simulations of the ARAF-MEK complex with ARAF C514A and C514N mutations show that both may have similar effects on the ARAF-MEK interface despite their differing effects on inhibitor potency. The mechanism of action of MEK inhibitors is itself complex, as it involves both stabilization of the MEK activation segment helix in a phosphorylation-resistant conformation and effects on the stability of the RAF-MEK interaction (*29*). Thus, differences across RAF isoforms in RAF-MEK affinity, in dynamic coupling between the RAF α_G_ helix and the MEK activation segment helix, as well as in direct contacts with the inhibitor, can all contribute to observed drug sensitivity. These considerations also apply to the effects of the point mutations studied here.

We leveraged the presence of Cys^514^ in ARAF to develop TWG-07-148, a trametinib analog containing an acrylamide warhead that covalently targets this residue, which is unique to ARAF. This compound exhibits ∼5-fold greater potency against ARAF-driven signaling compared to trametinib while maintaining comparable activity against BRAF and CRAF, providing proof of concept that the inherent ARAF-sparing tendency of current MEK inhibitors can be overcome through rational, covalent targeting of this residue.

The relative sparing of ARAF by current MEK inhibitors has important therapeutic implications, both positive and negative. The utility of anticancer therapeutics is always a balance between efficacy and tolerability, and a lack of potency on ARAF-MEK may contribute to the tolerability of MEK inhibitors because complete blockade of MAP kinase pathway signaling is not tolerated systemically. On the other hand, activating mutations in ARAF have been identified in Langerhans cell histiocytosis and in lung and other cancers (*37*–*39*), and existing MEK inhibitors may be less effective in these contexts as compared with BRAF or CRAF-driven cancers. Lack of potency against ARAF may also contribute to MEK inhibitor resistance. One widely observed mechanism of resistance to both RAF and MEK inhibitors is MAP kinase pathway reactivation due to relief of feedback inhibition (*56*–*58*). ERK signaling normally results in feedback inhibition of RAF activity, but RAF and MEK inhibitors can block this effect via receptor tyrosine kinase up-regulation and consequent RAS and RAF activation promoting recovery of ERK activity. A lack of potency against ARAF may contribute to this phenomenon, as RAFs both homodimerize and heterodimerize and can cooperate in promoting resistance (*59*). The extent to which TWG-07-148 can mitigate resistance due to pathway reactivation is an important area for future study. Most current RAF inhibitors are also ARAF sparing (*44*), which further emphasizes the potential value of more effective ARAF-MEK inhibitors.

Notwithstanding the potential confounding effects of RAF heterodimerization, ideally one would want to selectively block signaling from the specific RAF isoform activated in a given cancer. Our findings of varying potency of existing MEK inhibitors across RAF isoforms provide proof of principle for the feasibility of RAF-selective MEK inhibition, but we are not aware of deliberate efforts in the pharmaceutical industry to develop such an agent. Covalent targeting of Cys^514^ may offer an attractive approach to an ARAF-selective agent. While TWG-07-148 is not ARAF selective per se due to its high-affinity reversible binding to BRAF-MEK and CRAF-MEK complexes, its covalent mechanism may afford a degree of ARAF selectivity. Our Ba/F3 cell washout experiments reveal sustained suppression of ERK signaling in ARAF-MEK–driven cells as compared with cells expressing other RAF isoforms (Fig. 4H). The prolonged target engagement from covalent binding may enable sustained suppression of ARAF-induced ERK signaling while allowing recovery of signaling through BRAF and CRAF. Last, we speculate that a more selective ARAF-MEK inhibitor could be obtained by “detuning” its reversible affinity while maintaining the ability to form the covalent bond with ARAF.

Our study has several limitations. First, while our Ba/F3 cell models provide a controlled isogenic system for comparing MEK inhibitor potencies across RAF isoforms, these murine pro-B cells may not fully recapitulate the signaling context of human tumors driven by RAF or RAS mutations. Although we validated TWG-07-148 activity in human cancer cell lines harboring BRAF and KRAS mutations, we have not been able to identify an ARAF mutant or highly ARAF-dependent human cancer cell line that would allow us to more directly assess the advantage of its increased ARAF activity as compared with trametinib. Second, our biochemical assays used 14-3-3–bound RAF kinase domain dimers that lack the regulatory regions of the full-length protein that influence RAF activity, localization, and complex formation in cells (*6*). Differences between cellular and biochemical potencies across RAF isoforms and for mutations such as ARAF C514A likely stem from factors beyond the immediate RAF-MEK interface mediated by these regions in the cellular milieu. Third, while our kinome profiling of TWG-07-148 indicates excellent selectivity and its parent scaffold is exquisitely selective for MEK, we have not assessed potential proteome-wide off-target reactivity. Last, we have only evaluated the activity of TWG-07-148 in vitro—in vivo efficacy studies are an important future direction for this project.

In summary, our work establishes that RAF isoform identity is a critical determinant of MEK inhibitor potency, with current clinical agents exhibiting substantially reduced efficacy against ARAF-driven signaling. By identifying Cys^514^ as a unique structural feature of ARAF that can be exploited for covalent inhibition, we provide a rational strategy to overcome this limitation. While TWG-07-148 serves primarily as a proof-of-concept compound that will require further validation, it demonstrates that this covalent approach can meaningfully enhance inhibition of ARAF-MEK complexes. More broadly, our findings show that the view of MEK inhibitors as pathway selective but otherwise nondiscriminating agents is too simplistic; subtle differences in how these allosteric inhibitors engage different RAF-MEK complexes have functional consequences that could influence both therapeutic efficacy and resistance mechanisms. As our understanding of RAF isoform biology in cancer continues to evolve, the development of inhibitors tailored to specific RAF-driven contexts—whether through direct RAF kinase targeting or via RAF-selective MEK inhibition—represents a promising direction for improving outcomes in patients with MAP kinase pathway–driven malignancies.

## MATERIALS AND METHODS

### Expression and purification of MEK1–RAF(A/B/C)–14-3-3 complexes for assay

The RAF proteins were expressed and purified as previously described (*44*). Briefly, BRAF(419–766) and CRAF(308–648) constructs containing their kinase domains and C-terminal 14-3-3 binding sites were expressed in a baculovirus-mediated insect cell system. For ARAF(274–606), we used a previously described ARAF single-chain chimera in which 14-3-3 was genetically fused to the C-terminal end of the ARAF kinase domain (*44*). To express MEK1–RAF(A/B/C)–14-3-3 complexes, insect cells were coinfected with the corresponding baculoviruses. Cells were harvested 65 to 72 hours postinfection and lysed by sonication in buffer A [50 mM tris (pH 8.0), 150 mM NaCl, 10 mM MgCl_2_, 1 mM tris(2-carboxyethyl)phosphine (TCEP), and 1 μM adenylyl-imidodiphosphate (AMP-PNP)], and Halt protease inhibitor cocktail (Thermo Fisher Scientific). Clarified lysates were loaded onto Ni–nitrilotriacetic acid (NTA) agarose beads (QIAGEN), washed with buffer A containing 30 mM imidazole, and eluted with buffer A containing 250 to 300 mM imidazole. ARAF and CRAF complexes were further purified on StrepTrap HP columns (Cytiva). Eluted complexes were concentrated to 1 ml (Amicon Ultra centrifugal filter 30-kDa MWCO, Millipore) and subjected to SEC on Superose 6 10/300 or Superdex 200 Increase 10/300 column (Cytiva). SDS-PAGE confirmed coelution of MEK1 with the respective RAF kinase domain and 14-3-3. The resulting MEK1:RAF:14-3-3 complexes exhibited kinase activity in TR-FRET assays. ERK2 expressions and purifications as substrates for assay were done as previously described (*44*, *60*) using *Escherichia coli* BL21(DE3) cells.

### In vitro biochemical assay

Inhibition assays were performed using a modified cell-based HTRF phospho-ERK kinase assay kit (Revvity). We purified ERK protein in-house and used it as our substrate. Inhibitors were dispensed into black 384-well plates using an HP D300e dispenser and normalized to 1% final dimethyl sulfoxide (DMSO) concentration per well. Kit assay buffer was supplemented with purified RAF at a final concentration of 2 nM for MEK1-BRAF-14-3-3 and MEK1-CRAF-14-3-3 and 10 nM for MEK1-ARAF-14-3-3𝜀, as well as purified ERK at a final concentration of 500 nM. A total of 5 μl of supplemented kinase buffer was dispensed into 384-well plates using a Multidrop combi dispenser (Thermo Fisher Scientific) and incubated with inhibitors at room temperature for 40 min before reactions were initiated by adding 5 μl of 200 μM ATP. Plates were quenched after 2 hours at room temperature using the kit detection buffer supplemented with Anti–phospho-ERK1/2 antibodies (Revvity) and were incubated overnight at room temperature. The FRET signal ratio was measured at 665 and 620 nm using a PHERAstar microplate reader (BMG Labtech) and processed using GraphPad Prism fit to a three-parameter dose-response model. Assays were performed in triplicate three independent times.

### Expression and purification of ARAF-MEK1 with GDC-0879 for cryo-EM

For structural studies, residues 272 to 579 of the human ARAF kinase domain with SSDD mutations were inserted into an insect cell expression vector with an N-terminal maltose binding protein (MBP) tag and tobacco etch virus (TEV)–cleavable His_6_ tag. Cells coinfected with MEK1 virus were harvested ∼72 hours postinfection via centrifugation and lysed in buffer B (pH 7.4) (20 mM Hepes, 300 mM NaCl, 5 mM MgCl_2_, 1 mM TCEP, 1 μM AMP-PNP, 5% glycerol, 1 μM GDC-0879, and 1 μM TWG-07-148) and protease inhibitor cocktail (Thermo Fisher Scientific) via sonication. Clarified lysates were loaded onto Ni-NTA agarose beads (QIAGEN), washed with buffer B containing 30 mM imidazole, and eluted with buffer B containing 250 mM imidazole. The resulting fractions containing ARAF-MEK1 protein complex were concentrated and incubated with TEV protease (1:5 molar ratio) overnight to remove the MBP tag. ARAF-MEK1 protein complex was dialyzed against dialyzing buffer B.

The resulting sample was then subjected to another round of Ni-affinity purification (HisTrap, Cytiva), and the ARAF-MEK1 protein containing both the inhibitors was eluted along a 100-ml gradient from 0 to 50% of buffer B containing 250 mM imidazole. Fractions containing ARAF-MEK1 were pooled and immediately injected onto Superdex 200 16/10 column for SEC. SEC buffer did not contain any inhibitor. For plunging grids, the oligomeric ARAF-MEK1 (in complex with GDC-0879 and TWG-07-148) peak fractions were concentrated to 0.8 to 1 mg/ml immediately after SEC.

### Structure determination by cryo-EM

A total of 4 μl of ARAF-MEK1 with both GDC-0879 and TWG-07-148 prepared as above was applied to a glow-discharged QUANTIFOIL (Cu 1.2/1.3 300 mesh) grid, incubated for 10 s at 100% humidity and 10°C, blotted for 5 s, and plunge frozen in liquid ethane using a Leica EM GP (Leica Microsystems). Images were acquired on a 300-kV Titan Krios microscope (Thermo Fisher Scientific) equipped with a Falcon 4i direct electron detector and Selectris Energy Filter operated using EPU at Harvard Medical School Cryo–Electron Microscopy Laboratory.

Images were collected with a total exposure time of 2 s, a total dose of 50.98 e^−^/Å^2^, and a defocus range of −0.3 to −2.1 μm. Motion correction, CTF estimation, blob particle picking, and extraction were carried out in cryoSPARC (*61*). Particles were picked using an expected particle size of 80 to 160 Å and a box size of 360 pixels. The super-resolution images were binned by 2 to the physical pixel size of 0.736 Å during the extraction. A total of ∼6.5 million particles was selected by cryoSPARC using template-based picking and were subjected to 2D classification until clear, distinguishable secondary structural features were visible. Following the 2D classifications, ∼1.8 million particles were selected. Next, ab initio reconstructions were conducted with three classes. The resulting models were used for heterogeneous refinement, while a non–protein-like density was used as a decoy class. After a round of heterogeneous refinement, ∼775,000 particles were selected for nonuniform refinement using *C*_2_ symmetry. This set of particles (∼775,000) ultimately yielded a final map with a resolution of 2.51 Å (fig. S4, E and F). We obtained a second map with the same particle stack following symmetry expansion of these particles (new particle count: ∼1.5 million) with local refinement of the MEK1-ARAF-ARAF part of the volume (i.e., excluding one MEK1). Although this second map had a similar overall resolution of 2.55 Å, it exhibited improved density in the included MEK1 portion of the map, with degradation of the masked-out MEK1 as expected (fig. S4, E to G). The improvement is likely a consequence of the fact that the particles are not strictly *C*_2_ symmetric; the precise orientation of the MEKs with respect to the ARAF dimer is not identical on both sides of the dimer. The *C*_2_ symmetric map and the MEK1-ARAF-ARAF–focused map were deposited in the Electron Microscopy Data Bank (EMDB) under the accession code 74071. The overall scheme for the data processing is summarized in fig. S4E. 3D variability analysis was carried out with cryoSPARC using default parameters (*61*).

BRAF-MEK [Protein Data Bank (PDB): 4MNE] crystal structure was used as the reference to build the ARAF-MEK1 model. The initial model was then fit into the cryo-EM density in UCSF ChimeraX 1.5 (*62*). The model was refined against density map using Phenix 1.20 real space refinements with secondary structure restraints and no noncrystallographic symmetry constraints (*63*). The ARAF-MEK1 model was manually rebuilt and refined in the corresponding cryo-EM density map using Phenix 1.20 and WinCoot 0.9.8 (*63*, *64*). MolProbity was used to estimate the geometric restraints, clash score, and Ramachandran outliers (*65*).

### Generation of ARAF/BRAF/CRAF mutant Ba/F3 cells

Oncogenic kinase–transformed Ba/F3 cell lines were established as previously described (*66*). Briefly, 3 μg of ARAF/BRAF/CRAF mutant expression vector was cotransfected with 3 μg of psPAX2 and 0.3 μg of pMD2.G (the packaging plasmids) into 70 to 80% confluent 293T cells maintained in 3 ml of RPMI 1640 in a 60-mm dish by Lipofectamine 3000 (Thermo Fisher Scientific, catalog #L3000001)–mediated transfection. Forty-eight hours posttransfection, the viral supernatant was harvested and filtered with a 0.45-μm membrane.

For infection of Ba/F3 cells, 2.5 ml of viral supernatant and polybrene (8 μg/ml) were added to 2.5 million cells per well in six-well plates. Each plate was centrifuged at 2500 rpm for 90 min at 37°C for the spin infection. Forty-eight hours later, virus-containing medium was removed, and successfully infected cells were selected with the addition of puromycin (1 μg/ml) to the medium. After 3 days, cells were transferred into the fresh medium without IL-3 and puromycin. IL-3–independent transformed cells were maintained in RPMI 1640 supplemented with 10% fetal bovine serum. Expression of ARAF/BRAF/CRAF was examined by Western blot.

### Cell culture

The ARAF/BRAF/CRAF mutant Ba/F3, H2122, PC9, and HT29 cells were cultured in RPMI 1640 (Life Technologies, catalog #11875119) containing 10% fetal bovine serum (GeminiBio, catalog #100-106) and penicillin (100 U/ml) and streptomycin (100 μg/ml; Gibco, catalog #15140-22). A375 and A549 cells were cultured in Dulbecco’s modified Eagle’s medium (Life Technologies, catalog #11995073) containing 10% fetal bovine serum (GeminiBio, catalog #100-106) and penicillin (100 U/ml) and streptomycin (100 μg/ml; Gibco, catalog #15140-22). HCT116 cells were cultured in McCoy’s 5A medium (Life Technologies, catalog #16600082) containing 10% fetal bovine serum (GeminiBio, catalog #100-106) and penicillin (100 U/ml) and streptomycin (100 μg/ml; Gibco, catalog #15140-22). Parental Ba/F3 cells were cultured in RPMI 1640 (Life Technologies, catalog #11875119) containing 10% fetal bovine serum (GeminiBio, catalog #100-106), 200 ng of IL-3 (ProSpec, catalog #CYT-371), and penicillin (100 U/ml) and streptomycin (100 μg/ml; Gibco, catalog #15140-22). All the cell lines were cultured at 37°C in 5% CO_2_-humidified air and tested negative for mycoplasma.

### Antiproliferation assay

Cells were seeded at the density of 500 (adherent) or 1000 (suspension) cells per well in 384-well plates. For adherent cell lines, cells were cultured for 16 hours before compounds were added into the medium. The compounds were dispensed into the cells by HP D300e digital drug printer and incubated for 72 hours. Cell viability was determined using CellTiter-Glo (Promega, #G7571) according to the manufacturer’s instructions, measuring luminescence using a FLUOstar plate reader (BMG Labtech). Dose-response curves were generated using three-parameter nonlinear regression fits in GraphPad Prism (GraphPad Software). Data are presented as mean ± SEM (*N* = 3 or 4).

### Immunoblotting and antibodies

Cells were lysed in radioimmunoprecipitation assay buffer [150 mM NaCl, 1.0% IGEPAL CA-630, 0.5% sodium deoxycholate, 0.1% SDS, and 50 mM tris (pH 8.0)] (Sigma-Aldrich, catalog #R0278) with protease and phosphatase inhibitor (Thermo Fisher Scientific, catalog #1861282). The protein concentrations were measured by BCA analysis (Thermo Fisher Scientific, catalog #PI23225). Equal amounts of protein were resolved by 4 to 12% Tris-Base gels (Life Technologies) and then transferred to the Immuno-Blot PVDF membrane (Bio-Rad, catalog #1620177). Proteins were probed with appropriate primary antibodies at 4°C overnight and then with IRDye 800–labeled goat anti-rabbit immunoglobulin G (IgG; LICOR Biosciences, catalog #926-32211), IRDye 800–labeled goat anti-mouse IgG (LICOR Biosciences, catalog #926-32210), or IRDye 680RD goat anti-mouse IgG (LICOR Biosciences, catalog # 926-68070) secondary antibodies at room temperature for 1 hour. The membranes were imaged on an Odyssey CLx system.

Antibodies used in this study include anti-following proteins: pMEK1/2 (Ser^217/221^) (Cell Signaling Technology, #9154S; 1:1000), MEK1/2 (Cell Signaling Technology, #8727S; 1:1000), ERK1/2 (Cell Signaling Technology, #4696S; 1:1000), pERK1/2 (Cell Signaling Technology, #4370S; 1:1000), and β-actin (Cell Signaling Technology, #3700; 1:1000).

### Cell cycle analysis

Cells were treated with DMSO, TWG-07-148, or trametinib for 48 hours before harvesting and washing with cold phosphate-buffered saline (PBS). The cells were then fixed with cold 70% ethanol (prechilled at −20°C) and incubated at −20°C overnight. On the day of flow cytometry analysis, cells were collected by centrifugation, washed 3× with cold PBS, and stained in propidium iodide (PI) staining solution [PI (25 μg/ml), ribonuclease (0.2 mg/ml), and 0.1% Triton X-100 in PBS] for 30 min at room temperature and then analyzed by LSRFortessa flow cytometer (BD Bioscience, NJ, USA). Results were analyzed by FlowJo 10.1 software (www.flowjo.com/solutions/flowjo).

### Chemical compounds

TWG-07-148: *N*-(3-(3-cyclopropyl-5-((2-fluoro-4-iodophenyl)amino)-6,8-dimethyl-2,4,7-trioxo-3,4,6,7-tetrahydropyrido[4,3-d]pyrimidin-1(2H)-yl)phenyl)acrylamide.

A mixture of 1-(3-aminophenyl)-3-cyclopropyl-5-((2-fluoro-4-iodophenyl)amino)-6,8-dimethylpyrido[4,3-d]pyrimidine-2,4,7(1H,3H,6H)-trione (30 mg, 0.056 mmol), HATU (43 mg, 0.112 mmol), acrylic acid (4.3 μl, 0.062 mmol), diisopropylethylamine (29 μl, 0.168 mmol), and *N*,*N*-dimethylformamide (0.5 ml) was stirred at room temperature for 1.5 hours. The reaction mixture was purified by reversed-phase high-performance liquid chromatography (HPLC) using 0 to 80% ACN/H_2_O [trifluoroacetic acid (TFA) modifier] to give the title compound (4.9 mg, 14%). ^1^H nuclear magnetic resonance (NMR) (500 MHz, DMSO-*d_6_*), δ: parts per million 11.08 (s, 1H), 10.32 (s, 1H), 7.79 (dd, 1H), 7.70 (m, 2H), 7.56 (dd, 1H), 7.42 (t, 1H), 7.09 (dd, 1H), 6.93 (t, 1H), 6.43 (dd, 1H), 6.27 (dd, 1H), 5.78 (d, 1H), 3.08 (s, 3H), 2.63 (m, 1H), 1.27 (s, 3H), 0.96 (m, 2H), and 0.68 (m, 2H) (three protons masked by solvent); MS mass/charge ratio (*m/z*): 627.82 [M + 1]^+^.

1-(3-aminophenyl)-3-cyclopropyl-5-((2-fluoro-4-iodophenyl)amino)-6,8-dimethylpyrido[4,3-d]pyrimidine-2,4,7(1H,3H,6H)-trione was prepared according to the procedures in Abe *et al.* (*67*).

GD-01-012: *N*-(2-fluoro-3-((4-methyl-2-oxo-7-(pyrimidin-2-yloxy)-2H-chromen-3-yl)methyl)phenyl) acrylamide. A solution of 3-(3-amino-2-fluorobenzyl)-4-methyl-7-(pyrimidin-2-yloxy)-2H-chromen-2-one (50 mg, 0.13 mmol), diisopropylethylamine (34.5 μl, 0.20 mmol), and dichloromethane (2 ml) at 0°C was treated with acryloyl chloride (12.8 μl, 0.16 mmol). The reaction mixture was allowed to warm to room temperature, stirred for 15 min, and then concentrated under reduced pressure. The residue was purified by reversed-phase HPLC using 0 to 80% ACN/H_2_O (TFA modifier) to give the title compound (46 mg, 81%). ^1^H NMR (500 MHz, DMSO-*d_6_*) δ: 9.92 (s, 1H), 8.69 (d, 2H), 7.92 (d, 1H), 7.87 (m, 1H), 7.37 (d, 1H), 7.34 (t, 1H), 7.27 (dd, 1H), 7.06 (t, 1H) 6.96 (m, 1H), 6.62 (dd, 1H), 6.28 (dd, 1H), 5.78 (dd, 1H), 4.03 (s, 2H) (three protons masked by solvent peak); MS *m/z*: 432.11 [M + 1]^+^.

3-(3-amino-2-fluorobenzyl)-4-methyl-7-(pyrimidin-2-yloxy)-2H-chromen-2-one was prepared according to the procedures in Hyohdoh *et al.* (*68*). A complete experimental section detailing the synthesis and characterization for all 11 target compounds is included in the Supplementary Materials.

### MS analysis

Protein samples were acidified and desalted over C4 resin (ZipTip, Millipore). Samples were analyzed on an Orbitrap Eclipse mass spectrometer coupled to an Ultimate 3000 RSLCnano (Thermo Fisher Scientific). Proteins were separated over a 15-cm polystyrene divinylbenzene column (ES907, Thermo Fisher Scientific) with a 15-min gradient of 10 to 50% acetonitrile in 0.1% formic acid and electrosprayed (2 kV, 305°C) with an EasySpray ion source. Scans (600 to 2000 *m/z*) were obtained in the Orbitrap (15,000 resolution, profile). Deconvolution of the spectra and relative quantification of the peaks was done in the UniDec software (*69*). Percent labeling of proteins was calculated on the basis of the relative ratio of labeled species to the parent species. Here, 3 μM ARAF-MEK1 complex was treated with a fivefold excess concentration (1:5) of TWG-07-148 (15 μM) or GD-01-012 (15 μM).

### Kinome profiling

Kinome profiling of compound TWG-07-148 was performed at Reaction Biology using their Diversify Panel. This panel is a selection of 70 recombinant human kinases, which contains members from each kinase subfamily of human kinome, picked on the basis of protein conservation. Profiling used their radiometric HotSpot assay (*49*) with TWG-07-148 at a final concentration of 1 μM.

### PK studies

PK studies were conducted at the University of Florida Scripps Biomedical Research Institute. Male C57BL/6 mice strain received 1 mg/kg intravenously (iv) and 3 mg/kg PO dosage, after which concentrations of plasma and tissue samples harvested at various time points were quantified by MS. Briefly, 5-μl plasma samples were directly loaded to a 96-well Millipore MultiScreen Solvinert 0.45-μm low-binding hydrophilic polytetrafluoroethylene filter plate. Tissue samples were homogenized with water (×3 dilution), and then 5 μl was loaded to the filter plate. All plasma/tissue samples were treated with 75 μl of 90/10 acetonitrile/water with carbamazepine as IS to extract the analyte and precipitate protein. The plates were agitated on ice for ∼10 min before centrifugation into a collection plate. Separate standard curves were prepared in blank mouse plasma and tissue homogenate and processed in parallel with the samples. The filtrate was directly analyzed by LC-MS/MS.

### Molecular dynamics protocol

Initial structures were derived from the TWG-07-148 ARAF/MEK structure. Protein structures were prepared using the Protein Preparation Wizard in Schrödinger Suite (*70*). The TWG-07-148 covalent bond to ARAF C514 was broken. The terminal carbon of the acrylamide group was removed to convert TWG-07-148 into trametinib. Mutant systems were prepared by muting residue 514 in Maestro (*64*). A local minimization of 0.5 nm around the trametinib was performed to remove possible steric clashes. Ligands were extracted from the system for parameterization. Ligand parameters were generated using the AMBER ff19SB force field (*71*). Small-molecule structures were processed using AmberTools suite (*72*). AM1-BCC charges were calculated using Antechamber, with the GAFF2 force field parameters assigned via parmchk2 (*73*). Protein-ligand complexes were prepared by solvating the systems in a TIP3P water box with a padding of 1.2 nm. Counterions were added at a physiological ionic strength of 0.1 M. Energy minimization was performed using a convergence-based approach with a force tolerance of 1.0 kJ/mol per nanometer and an energy tolerance of 0.1 kJ/mol over 50 steps. The systems were equilibrated using a two-phase approach. A heating phase was conducted starting from 50 K and gradually increasing to 310 K over 2.5 ns while then simultaneously reducing restraints on protein backbone atoms from 10.0 to 0.0 kJ/mol per square nanometer during the second 1.25 ns. Production simulations were performed in the NPT ensemble at 310 K and 1.0 bar, using the Langevin integrator with a friction coefficient of 1.0 ps^−1^ and Monte Carlo barostat with a frequency of 25 steps. Hydrogen mass repartitioning was used with a hydrogen mass of 4.0 Da, allowing for an extended timestep of 4.0 fs (*74*). All simulations were performed in triplicate with different initial velocities to ensure statistical significance, with each production run extending for 500 ns, yielding a total simulation time of 1.5 μs. Nonbonded interactions were truncated at 1.0 nm, and all covalent bonds involving hydrogen atoms were constrained using the SHAKE algorithm (*75*, *76*). System stability during equilibration was confirmed by monitoring total energy and root mean square deviation convergence. Simulations were analyzed via MDTraj (*77*). A separate postprocessing protocol was implemented to quantify ligand-protein interaction energies from the production trajectories using OpenMM. For each saved frame, energy evaluations were performed, which were then averaged over the last 2500 frames to obtain mean and SD values per system. All stages, from force-field assignment to simulation execution, were automated using custom Python workflows (available on GitHub). Scripts are available at https://github.com/dgazgalis/RAF-isoform-selectivity-of-MEK-inhibitors. Structural biology software used in this study was curated by SBGrid (*78*).
