# Supplementary material for: RAF isoform selectivity of MEK inhibitors and rational design of a covalent ARAF-MEK inhibitor
Source: Sci Adv. 2026 Oct 23;12(43):eaee5241. doi: 10.1126/sciadv.aee5241 (PMC13693665; doi:10.1126/sciadv.aee5241)
Supplement: Supplementary file 1 [file sciadv.aee5241_sm.pdf]

Science Advances
AAAS

Supplementary Materials for

RAF isoform selectivity of MEK inhibitors and rational design of a covalent ARAF-MEK inhibitor

Sayan Chakraborty *et al.*

Corresponding author: Michael J. Eck, Michael_Eck@dfci.harvard.edu

Sci. Adv. **12**, eaee5241 (2026)
DOI: 10.1126/sciadv.aee5241

The PDF file includes:

Figs. S1 to S7
Tables S1 to S6
Supplementary Text
Legend for movie S1

Other Supplementary Material for this manuscript includes the following:

Movie S1

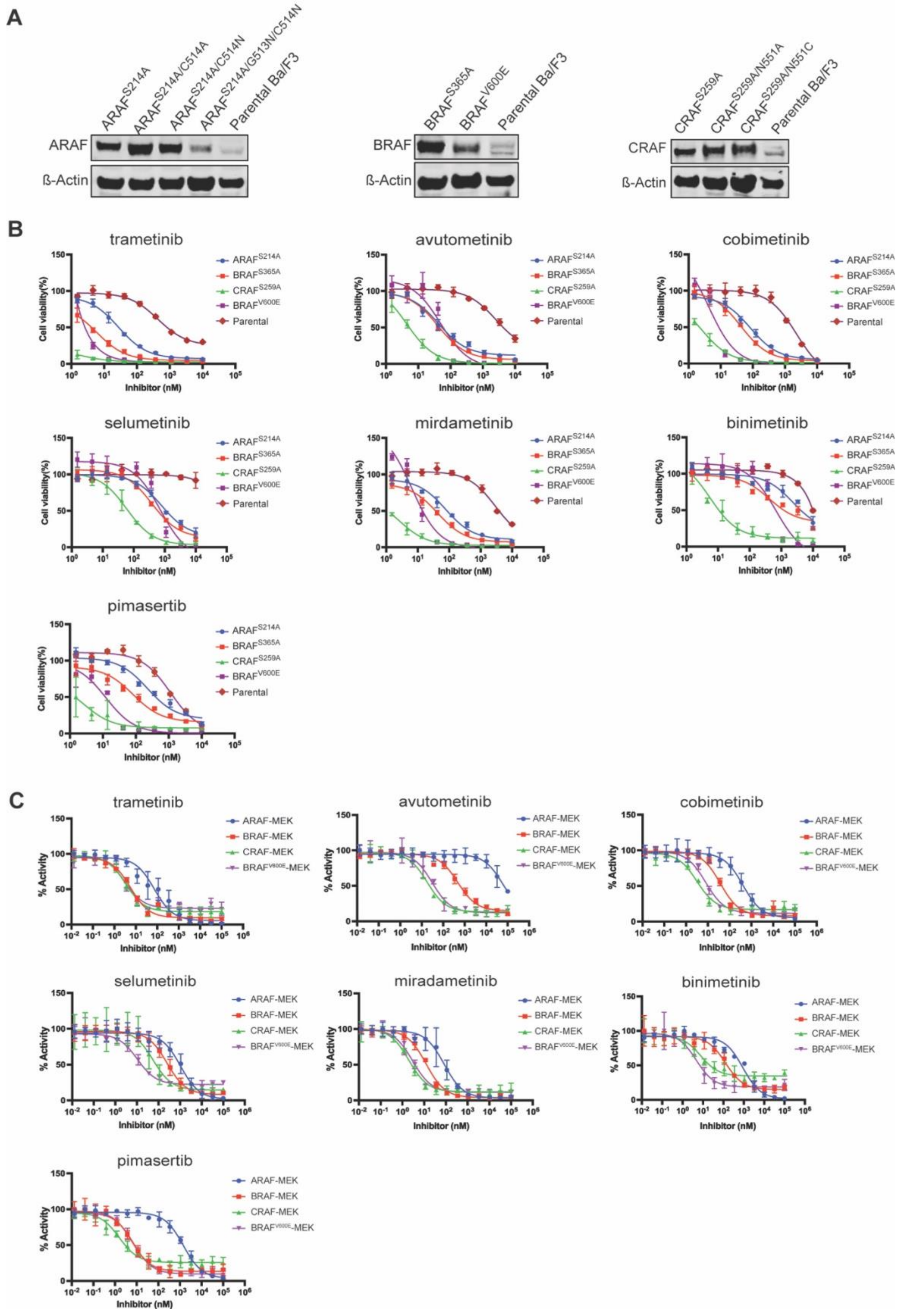

Fig. S1. Ba/F3 cell expression of activated RAFs and dose response curves for allosteric MEK inhibitors across RAF isoforms. (**A**) Immunoblot analysis of engineered Ba/F3 cells expressing constitutively active RAFs (ARAF^{S214A}, BRAF^{S365A}, CRAF^{S259A}, BRAF^{V600E}) and their corresponding mutant variants. (**B**) Dose-response curves for seven allosteric MEK inhibitors against engineered Ba/F3 cell lines expressing ARAF^{S214A},

ARAF$^{S214A/C514A}$, BRAF^{S365A}, V600E, and CRAF^{S259A}. (**C**) Dose response curves for seven allosteric MEK inhibitors in a biochemical cascade assay that measures phosphorylation of ERK2 by ARAF-MEK1, BRAF-MEK1, CRAF-MEK1, and BRAF^{V600E}-MEK1 complexes.

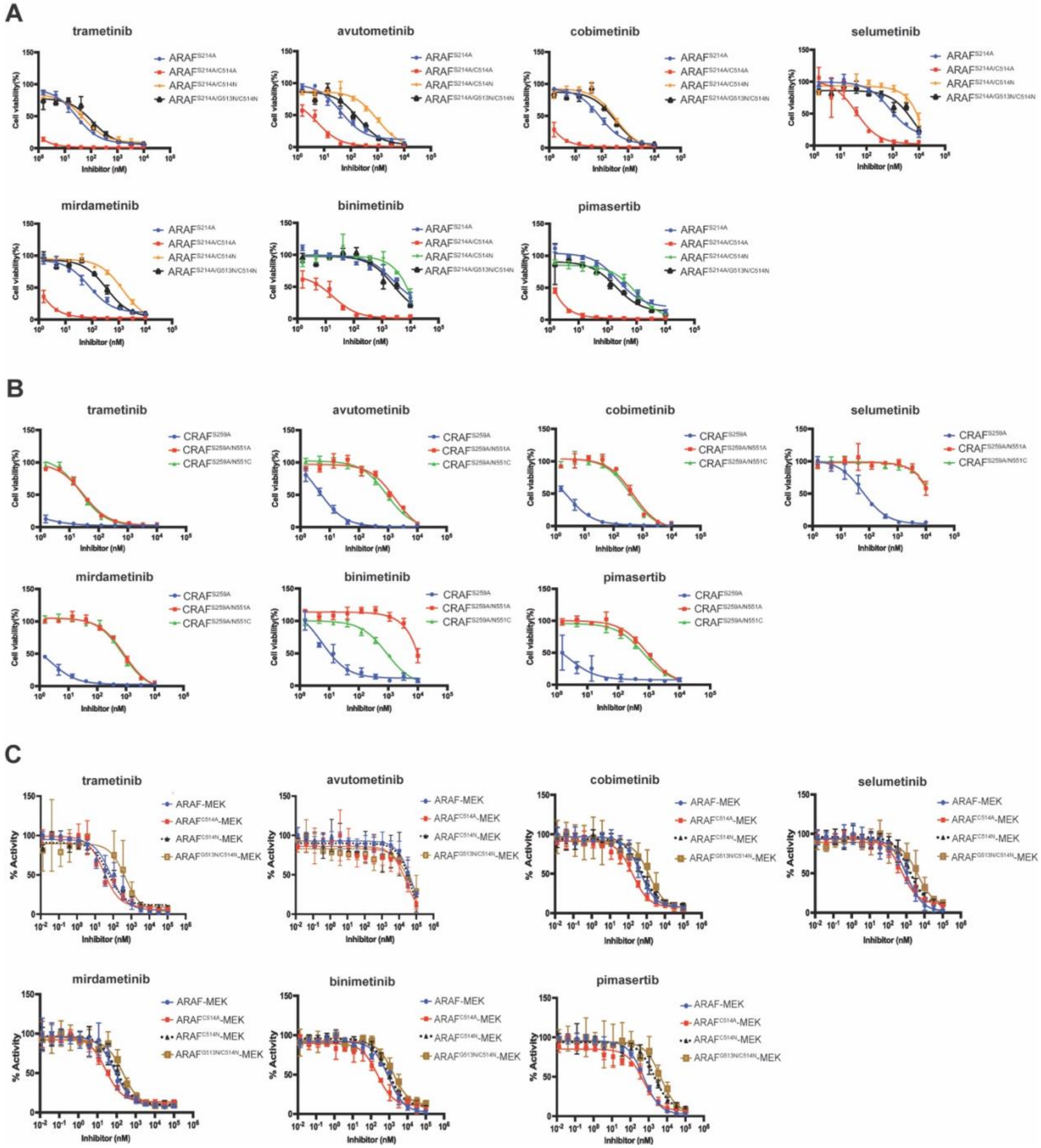

Fig. S2. Dose response curves for allosteric MEK inhibitors against ARAF and CRAF αG- helix mutants. (**A**) Cell viability assay assessing the potency of seven allosteric MEK inhibitors in engineered Ba/F3 cells expressing constitutively active ARAF^{S214A} with and without mutations in the ARAF portion of the inhibitor binding pocket (αG-helix mutants C514A, C514N, and G513N/C514N). (**B**) Cell viability assay assessing the potency of allosteric MEK inhibitors in engineered Ba/F3 cells expressing constitutively active CRAF^{S259A} with and without mutations in the CRAF portion of the inhibitor binding pocket (αG-helix mutants N553A and N553C). (**C**) Dose response curves for allosteric MEK inhibitors in a biochemical cascade assay that measures phosphorylation of ERK2 by ARAF-MEK1, with or without mutations in the ARAF portion of the inhibitor binding pocket (αG-helix mutants C514A, C514N, and G513N/C514N).

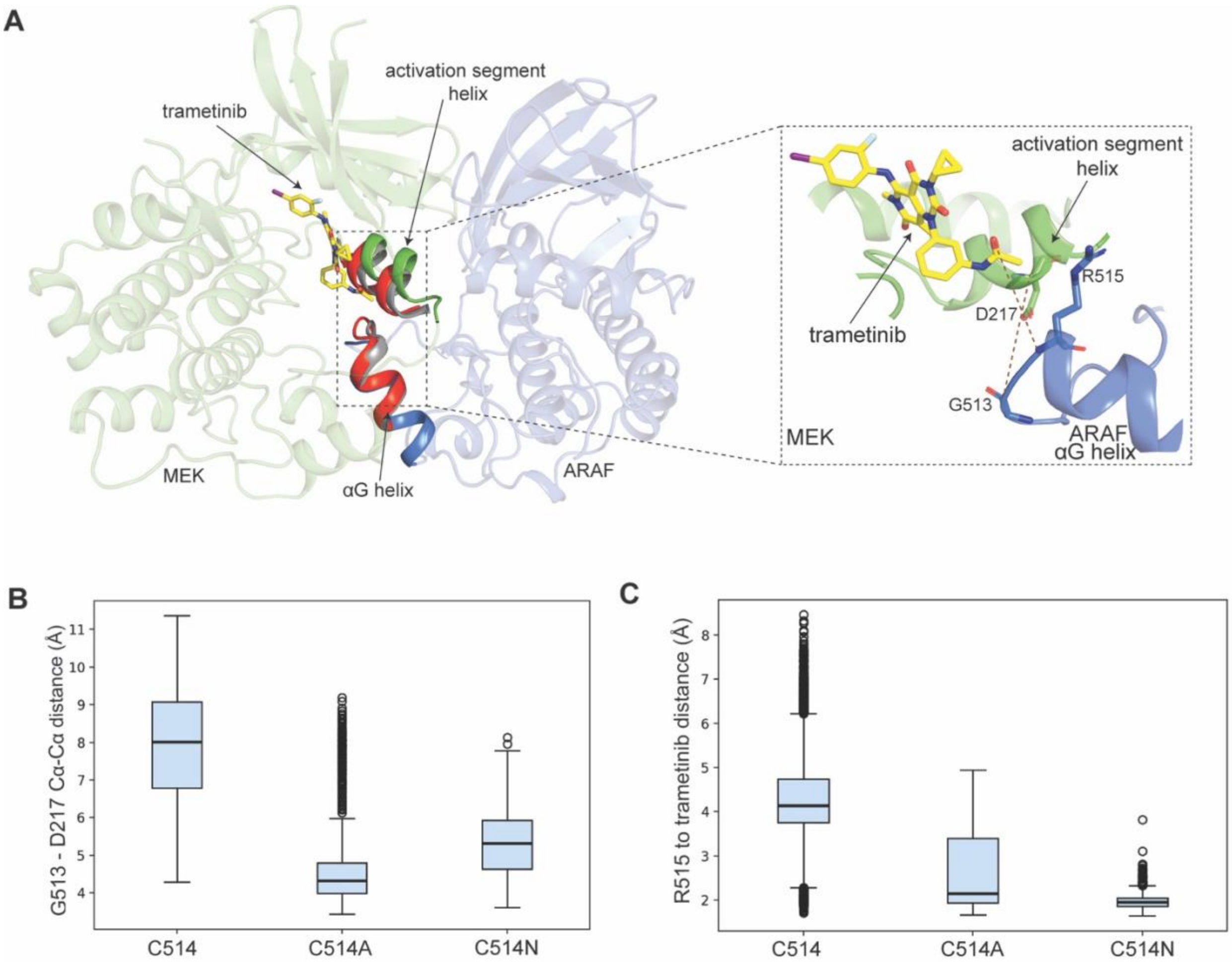

D

Calculated protein-ligand interaction energies

System	Coulomb interaction (kcal/mol)	LJ interaction (kcal/mol)	Total Interaction (kcal/mol)
ARAF	-21.08 ± 5.22	-71.90 ± 3.35	-92.99 ± 6.07
ARAF C514A	-27.91 ± 4.31	-75.42 ± 3.19	-103.33 ± 4.69
ARAF C514N	-29.55 ± 4.03	-71.58 ± 2.93	-101.13 ± 4.66

Fig. S3. Molecular dynamics analysis of the effect of ARAF αG-helix mutations on the ARAF-MEK protein complex and trametinib binding. (**A**) Molecular dynamics simulations reveal subtle shifts in the positions of the activation segment helix of MEK1 and the N-terminal portion of the αG-helix of ARAF in the C514A and C514N mutants as compared with WT ARAF. Wild-type ARAF is shown in blue and MEK in green. Mutants C514A (red) and C514N (grey) are superposed on the wild-type ARAF-MEK complex. The inset illustrates the distance measurements plotted in panels b and c. (**B**) Box and whisker plots of the distance between ARAF G513 and MEK D217 (Cα-Cα) in wild-type versus C514A and C514N mutants reveal closer apposition of the MEK1 activation segment helix and the αG-helix of ARAF as seen in panel A. These distances were derived from MD simulation. (**C**) Box and whisker plots of the distance between the backbone amide NH of Arg515 in ARAF and the carbonyl oxygen of trametinib in wild-type versus C514A and C514N mutants indicate that this hydrogen bond is more stably formed in the ARAF mutants. (**D**) Calculated interaction energies between ARAF (wild-type and mutants) and trametinib obtained from the molecular dynamics simulations are shown in a tabulated form. Simulations were carried out for a total of 500 ns, analysis in **A-D** corresponds to mean position over final 250 ns of the simulation.

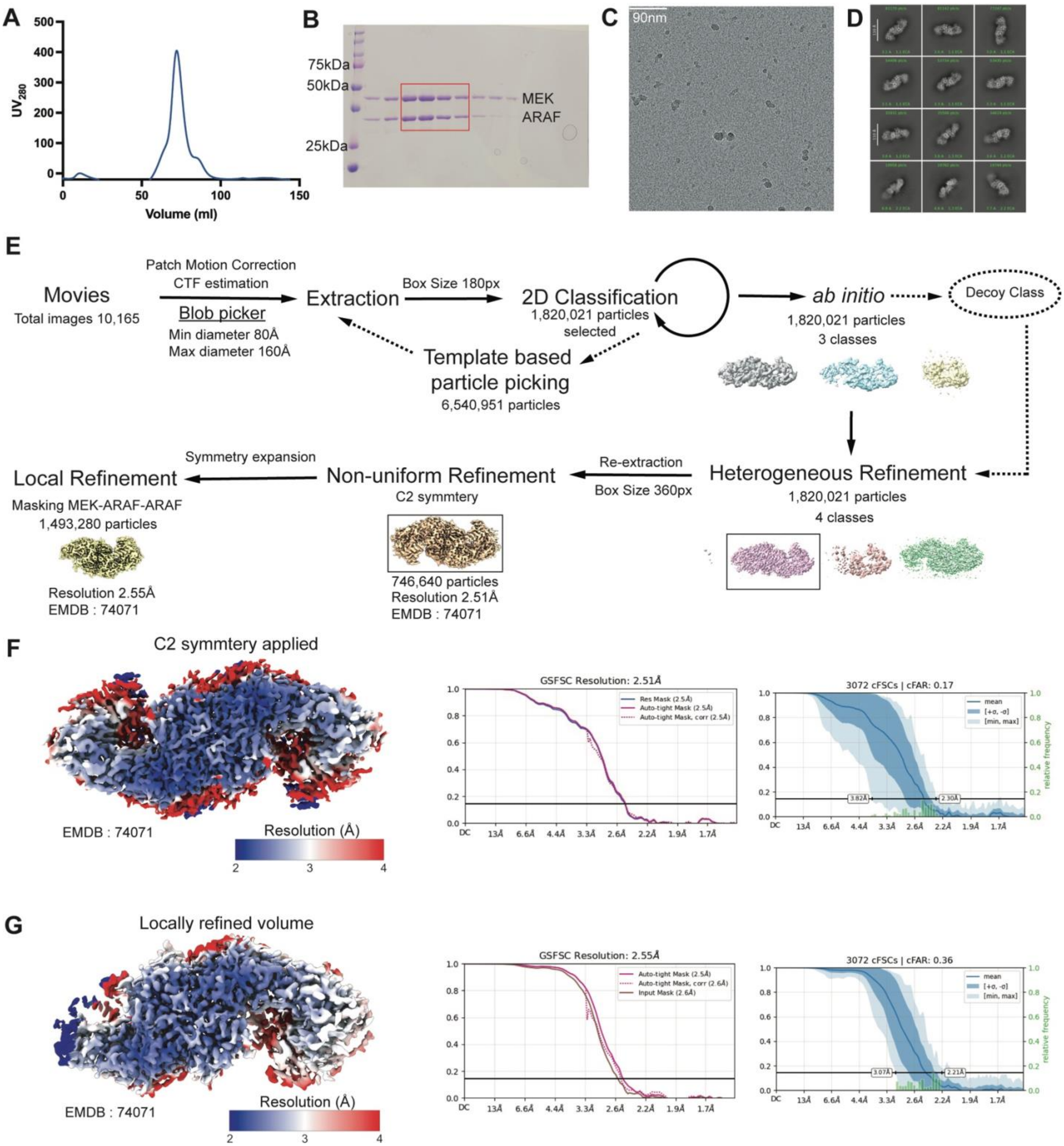

Fig. S4. ARAF-MEK1 protein production and cryo-EM reconstruction of ARAF-MEK1 complex. (A) Size exclusion chromatography (SEC) profile of purified ARAF-MEK1 with GDC-0879 and TWG-07-148. Note that the ARAF construct corresponds to the kinase domain (residues 272 to 579, see *Methods* for further details). (**B**) SDS-PAGE analysis of SEC elution fractions obtained in the presence of inhibitors. The red box indicates fractions that were pooled and used for cryo-EM imaging. (**C**) Representative micrograph (**D**) 2D class averages of ARAF-MEK1 with GDC-0879 and TWG-07-148 obtained from CryoSPARC showing clear secondary structural features. (**E**) Schematic diagram showing the workflow for cryo-EM image processing. Image processing was done entirely in CryoSPARC. Classes with black boxes were selected for further

processing. (**F**) Cryo-EM density map of the tetrameric assembly (C2 symmetry applied) colored by cryoSPARC estimated local resolution. Structural validation is provided by Gold Standard Fourier shell correlation (GSFSC) curves and the cFAR value, showing the overall resolution and the relative directional anisotropy of the final cryo-EM reconstruction, respectively. (**G**) Locally refined cryo-EM reconstruction colored by local resolution (estimated in CryoSPARC) showing the gradual omission of one MEK1 protomer from the ARAF homodimer (see *Methods* for further details). Gold Standard Fourier shell correlation (GSFSC) curves indicate the estimated resolution of the trimeric volume. The cFAR value illustrates the relative anisotropy of the final reconstruction.

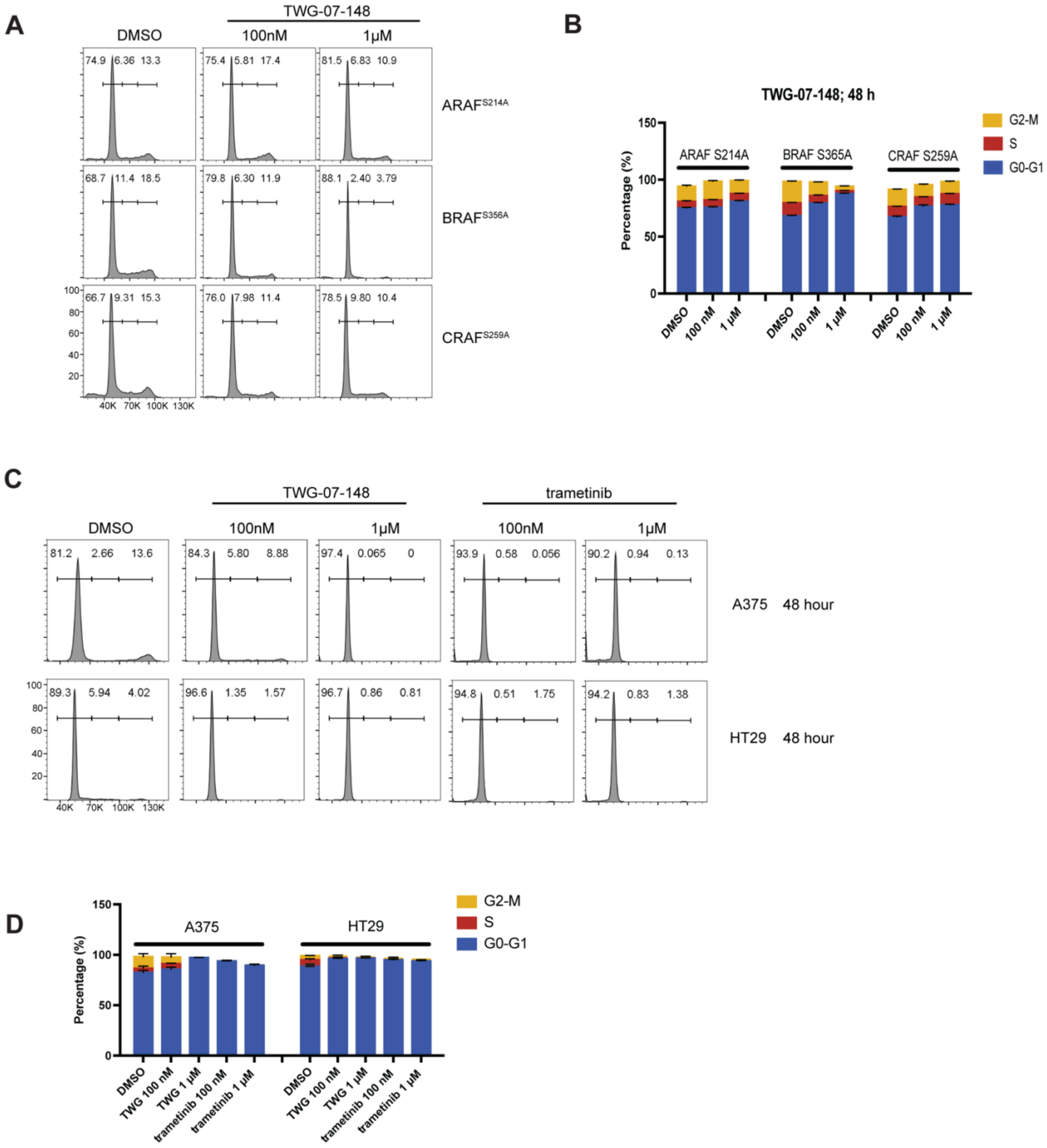

Fig. S5. TWG-07-148 and trametinib induce cell cycle arrest. (**A**) Flow cytometry cell cycle analyses of Ba/F3 cells transformed with $ARAF^{S214A}$, $BRAF^{S365A}$, or $CRAF^{S259A}$ and treated with TWG-07-148 (100 nM or 1 µM) or DMSO control. (**B**) Quantitation of fraction of cells in differing stages of the cell cycle (G_0-G_1, S, M) from panel **A**. (**C**) Flow cytometry cell cycle analyses of A375 and HT29 cells treated with TWG-07-148 (100 nM or 1 µM) or trametinib (100 nM or 1 µM). (**D**) Quantitation of fraction of cells in differing stages of the cell cycle (G_0-G_1, S, M) from panel **C**.

Inhibitor	Ba/F3 cell IC_{50} (nM)				
	$ARAF^{S214A}$	$BRAF^{S365A}$	$CRAF^{S259A}$	$BRAF^{V600E}$	Parental
trametinib	28.9±0.06	3.1±1.1	<1	3.8±0.3	>1000
avutometinib	82.3±19.1	44.1±6.3	7.2±2.4	81.7±12.9	>1000
cobimetinib	88.6±14.9	37.5±13.0	4.3±3.1	11±0.7	>1000
mirdametinib	100.8±8.6	31.4±4.7	1.7±1	11.6±0.8	>1000
selumetinib	>1000	707.3±38.0	84.5±22.0	788.3±96.5	>1000
binimetinib	>1000	250.2±188.0	16.3±1.3	605.2±0.3	>1000
pimasertib	119±12.4	74.6±5.3	<1	14±2.3	>1000
TWG-07-148	5.5±0.1	5.3±2.8	<1	3.5±0.7	>1000

Table S1. Ba/F3 cell IC_{50} values for MEK inhibitors against constitutively active RAF isoforms.*

*IC_{50} values correspond to dose response data plotted in Supplementary Fig. S1B and Fig. 1A in the main text. Data are presented as mean ± SEM (n = 3 or 4).

Inhibitor	ARAF/MEK	BRAF/MEK	CRAF/MEK	$BRAF^{V600E}$/MEK
trametinib	72.9±9.21	4.5±1	4.2±2.7	6.3±2.2
avutometinib	>1000	491±41	18.4±3.8	92±51.9
cobimetinib	324.3±107.02	47.6±21	2.8±0.5	4±2.4
mirdametinib	49.6±13.6	10.6±3.1	1.8±0.2	2±0.9
selumetinib	>1000	240±11.9	41.7±20.8	21±10
binimetinib	>1000	119.8±78.6	13±1.6	11±5.6
pimasertib	855±275.6	41.1±29.8	3.14±0.18	3.8±1.5

Table S2. Biochemical IC_{50} values (nM) for MEK inhibitors against purified RAF-MEK complexes.*

*IC_{50} values correspond to dose response data plotted in Supplementary Fig. S1C and Fig. 1C in the main text. Data are presented as mean ± SD (n = 3).

Inhibitor	ARAF^{S214A} IC$_{50}$ (nM)				CRAF^{S259A} IC$_{50}$ (nM)		
	C514	C514A	C514N	G513N/C514N	N553	N553A	N553C
trametinib	28.9±0.06	<1	131.5±34.7	79.8±53.7	<1	44.9±19.5	43.6±20.2
avutometinib	82.3±19.1	21.6±11.7	825.3±63.2	261.8±191.2	7.2±2.4	>1000	>1000
cobimetinib	88.6±14.9	3.4±0.9	356.2±20.2	259.6±73.9	4.3±3.1	499.5±88.8	503.2±191.1
mirdametinib	100.8±8.6	7.7±7	1044±81.6	514.2±275.6	1.7±1	931±141.4	908.1±231.8
selumetinib	>1000	41.3±54.5	>1000	>1000	84.5±21.9	>1000	>1000
binimetinib	>1000	12.6±10.5	>1000	>1000	16.3±1.3	>1000	>1000
pimasertib	119±12.4	5.1±5.3	758.8±72.7	473.3±356.1	<1	>1000	990.2±524.3
TWG-07-148	5.5±0.1	<1	95.9±6.1	55.6±12.6	<1	145.2±70.0	119.1±57.5

Table S3. Ba/F3 cell IC$_{50}$ values for MEK inhibitors against ARAF and CRAF variants.*

*IC$_{50}$ values correspond to dose response data plotted in Supplementary Fig. S2A, B and Fig. 2C, D in the main text. Data are presented as mean ± SEM (n = 3 or 4).

Inhibitor	ARAF/MEK IC_{50} (nM)			
	C514	C514A	C514N	G513N/C514N
trametinib	72.9±9.21	25.9±16.5	34.9±18.7	18±9.1
avutometinib	>1000	>1000	>1000	>1000
cobimetinib	324.3±107.02	181.1±22.7	337.3±81.7	765±164.4
mirdametinib	49.6±13.6	29.6±5.8	53.48±20.5	130.6±2.2
selumetinib	>1000	539.2±94	>1000	>1000
binimetinib	>1000	296.5±48	651.4±34.8	>1000
pimasertib	855±275.6	523.3±77	>1000	>1000

Table S4. Biochemical IC_{50} values for MEK inhibitors against purified ARAF variants.*

*IC_{50} values correspond to dose response data plotted in Supplementary Fig. S2C. Data are presented as mean ± SD (n = 3).

Data collection and processing	PDB : 9ZDQ<br>EMDB : 74071
Magnification	165,000x
Voltage (kV)	300
Electron exposure (e–/Å2)	50.98
Defocus range (μm)	-0.3 to -2.1
Pixel size (Å)	0.736
Symmetry imposed	C2
Initial particle images (no.)	6,540,951
Final particle images (no.)	746,640
Map resolution (Å)	2.51
FSC threshold	0.143
Map resolution range (Å)	2.2/3/3.8
**Refinement**	
Initial model used (PDB code)	4MNE
Model resolution (Å)	2.7
FSC threshold	0.143
Model resolution range (Å)	2.4/2.5/2.8
Map sharpening B factor (Å2)	96.7
**Model composition**	
Non-hydrogen atoms	8756
Protein residues	1082
Ligands	T14:2/29L:2/ANP:2
**B factors (Å2)**	
Protein	9.88/147.71/67.95
Ligand	18.18/128.78/69.40
**R.m.s. deviations**	
Bond lengths (Å)	0.002
Bond angles (°)	0.538
**Validation**	
MolProbity score	2.31
Clashscore	7.49
Poor rotamers (%)	5.72
**Ramachandran plot**	
Favored (%)	95.31
Allowed (%)	4.69
Outlier	0.00

Table S5. Cryo-EM data collection, refinement, and validation statistics

Kinase Profiling Screening
% Enzyme Activity (relative to DMSO controls)

Kinases	TWG-07-148 (1uM) Data 1	TWG-07-148 (1uM) Data 2	IC50 (M) Control Cmpd	Control Cmpd ID
ABL1	95.89	93.53	1.06E-05	STAUROSPORINE
AKT1	101.57	99.69	4.47E-07	STAUROSPORINE
ALK4/ACVR1B	94.80	93.82	1.45E-05	LDN193189
ARK5/NUAK1	105.25	99.75	1.84E-09	STAUROSPORINE
Aurora A	88.54	87.60	4.97E-08	STAUROSPORINE
BRAF	88.93	88.85	3.07E-08	GW5074
BTK	103.90	101.75	1.41E-06	STAUROSPORINE
c-Kit	98.00	95.89	1.41E-09	STAUROSPORINE
c-MET	112.69	99.53	1.63E-06	STAUROSPORINE
c-Src	103.16	98.93	6.39E-08	STAUROSPORINE
CAMK1a	95.76	94.88	9.83E-07	STAUROSPORINE
CDK1/cyclin B	110.99	106.53	1.41E-08	STAUROSPORINE
CDK4/cyclin D1	107.04	103.00	3.32E-08	STAUROSPORINE
CDK7/cyclin H	105.47	105.32	6.54E-07	STAUROSPORINE
CDK9/cyclin T2	93.72	93.01	5.05E-08	STAUROSPORINE
CHK1	107.39	104.86	7.76E-10	STAUROSPORINE
CK1a1	100.06	97.08	3.58E-06	MU1742
CK1g1	93.97	93.26	1.56E-06	NERATINIB
CK2a	112.74	110.91	3.54E-05	GW5074
CLK2	100.07	96.48	6.01E-08	STAUROSPORINE
DAPK2	87.67	85.22	2.96E-07	STAUROSPORINE
DCAMKL1	99.38	98.65	1.19E-06	STAUROSPORINE
DYRK1/DYRK1A	104.23	102.86	7.99E-08	STAUROSPORINE
EGFR	94.32	94.01	4.39E-06	STAUROSPORINE
EPHA5	104.26	103.92	2.27E-06	STAUROSPORINE
EPHB2	104.36	103.36	2.85E-05	STAUROSPORINE
ERK1	103.76	101.89	1.59E-08	SCH772984
FGFR2	104.23	100.94	6.77E-08	STAUROSPORINE
FLT1/VEGFR1	92.79	92.23	9.72E-08	STAUROSPORINE
FLT3	104.74	104.48	4.43E-09	STAUROSPORINE
GSK3b	94.65	92.32	3.62E-07	STAUROSPORINE
HIPK2	96.44	95.90	1.24E-05	STAUROSPORINE
IGF1R	95.16	94.67	3.49E-06	STAUROSPORINE
IKKe/IKBKE	121.42	114.67	1.08E-09	STAUROSPORINE
IRAK4	98.25	96.91	1.48E-08	STAUROSPORINE
JAK2	94.93	92.35	6.19E-09	STAUROSPORINE
JNK3	103.72	98.24	9.04E-06	JNKI VIII
LIMK1	115.94	109.27	3.02E-08	STAUROSPORINE
LOK/STK10	102.73	102.60	5.40E-07	RO-31-8220
LYN	95.97	95.68	3.48E-08	STAUROSPORINE
MARK2/PAR-1Ba	102.63	96.03	2.78E-10	STAUROSPORINE
MAST3	103.68	99.57	1.14E-06	DOVITINIB
→ MEK1	25.35	24.72	2.29E-07	STAUROSPORINE
MEKK1	111.53	106.94	1.28E-04	STAUROSPORINE
MLK1/MAP3K9	100.70	100.61	2.15E-09	STAUROSPORINE
MNK2	91.36	90.91	3.20E-08	STAUROSPORINE
MSK2/RPS6KA4	107.69	97.97	1.20E-07	STAUROSPORINE
MST1/STK4	91.22	91.03	3.35E-09	STAUROSPORINE
MYLK3	104.05	98.62	6.71E-06	STAUROSPORINE
NEK1	93.29	90.38	2.85E-07	STAUROSPORINE
P38a/MAPK14	100.10	99.14	6.58E-08	SB202190
p70S6K/RPS6KB1	98.50	96.36	3.80E-09	STAUROSPORINE
PAK4	113.12	111.84	5.97E-07	STAUROSPORINE
PIM3	107.25	100.10	3.73E-09	STAUROSPORINE
PKA	104.88	101.04	9.30E-08	STAUROSPORINE
PKCa	101.37	101.11	5.23E-09	STAUROSPORINE
PKCtheta	103.69	98.47	8.03E-09	STAUROSPORINE
PLK1	100.17	99.78	1.11E-08	BI2536
RET	88.24	86.41	2.15E-07	STAUROSPORINE
ROCK1	99.53	96.69	2.14E-08	STAUROSPORINE
RSK1	106.02	105.63	1.90E-09	STAUROSPORINE
STK22D/TSSK1	93.31	93.13	2.51E-09	STAUROSPORINE
TEC	109.39	102.59	1.49E-05	STAUROSPORINE
TGFBR2	105.45	100.87	1.42E-05	LDN193189
TRKA	102.82	99.01	8.63E-10	STAUROSPORINE
ULK1	103.59	99.30	6.48E-08	STAUROSPORINE
PI3Ka (p110a/p85a)	89.32	87.68	5.98E-08	PI-103
PI3Kb (p110b/p85a)	99.93	102.82	2.94E-07	PI-103
PI3Kd (p110d/p85a)	98.16	98.29	2.97E-07	PI-103
PI3Kg (p110g)	102.32	101.50	1.93E-06	PI-103

Table S6. Kinome-wide selectivity of TWG-07-148 against a panel of 70 different human kinases

Movie S1:

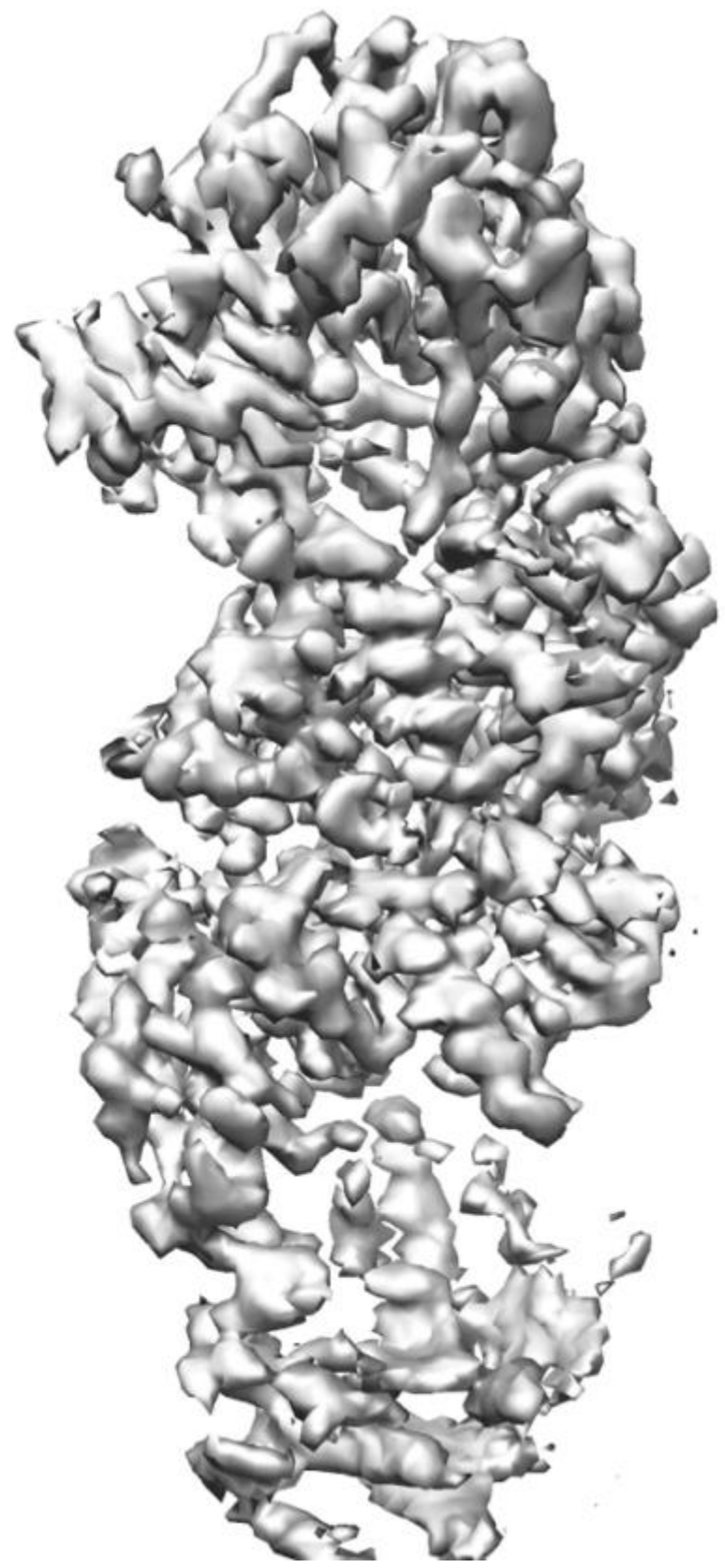

Synthesis and Characterization for all 11 target compounds

Chemistry

Analytical grade solvents and commercially available reagents were purchased from commercial sources and used directly without further purification unless otherwise stated. Thin-layer chromatography (TLC) was carried out on Merck 60 F254 precoated, glass silica plates which were visualized with either ultraviolet light (λ = 254 nm) or stained with $KMnO_4$. Experiments were conducted under ambient conditions unless otherwise stated. ^1H NMR and ^{13}C NMR spectra were recorded at room temperature using a Bruker 500 (^1H NMR at 500 MHz and ^{13}C NMR at 126 MHz). Chemical shifts are reported in ppm with reference to solvent signals [^1H NMR: $CDCl_3$ (7.26 ppm) and DMSO-d_6 (2.50 ppm); ^{13}C NMR: DMSO-d_6 (39.52 ppm)]. Signal patterns are indicated as: s, singlet; *br* s, broad singlet; d, doublet; dd, doublet of doublets; ddd, doublet of doublets of doublets; dt, doublet of triplets; t, triplet; td, triplet of doublets; q, quartet; p, pentet; and m, multiplet. Mass spectrometry (MS) analysis was obtained on a Waters Acquity UPLC-MS system using electrospray ionization (ESI) in positive ion mode, reporting the molecular ion $[M+H]^+$, or a suitable fragment ion. Flash chromatography purification was conducted using an ISCO CombiFlash RF+ with RediSep Rf silica cartridges. Preparative reverse-phase HPLC purification was conducted using a ISCO ACCQPrep HP125 using RediSep Prep C18 column (20x150 mm, 20 mL/min flow rate), and a gradient solvent system of water (0.035% TFA) / acetonitrile (0.035% TFA).

Chemical compounds

TWG-07-148 *N*-(3-(3-cyclopropyl-5-((2-fluoro-4-iodophenyl)amino)-6,8-dimethyl-2,4,7-trioxo-3,4,6,7-tetrahydropyrido[4,3-d]pyrimidin-1(2H)-yl)phenyl)acrylamide

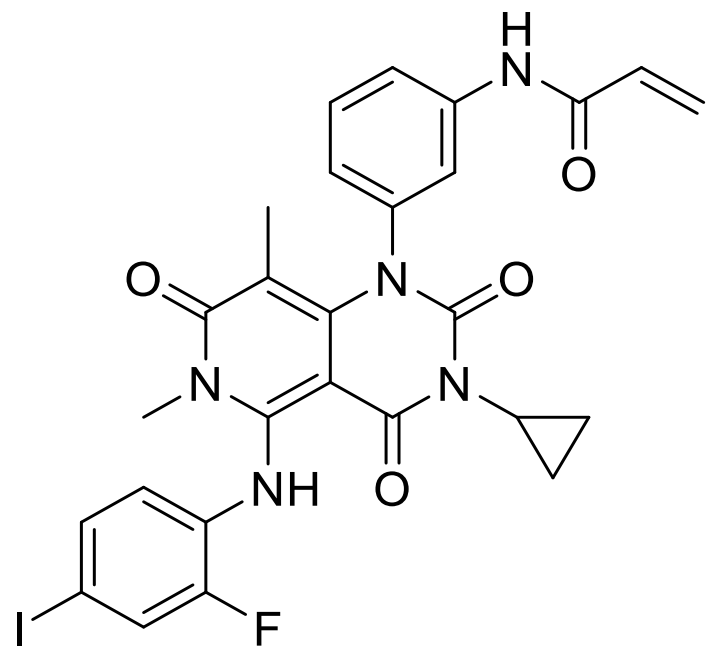

A mixture of 1-(3-aminophenyl)-3-cyclopropyl-5-((2-fluoro-4-iodophenyl)amino)-6,8-dimethylpyrido[4,3-d]pyrimidine-2,4,7(1H,3H,6H)-trione (30 mg, 0.056 mmol), HATU (43 mg, 0.112 mmol), acrylic acid (4.3 uL, 0.062 mmol), diisopropylethylamine (29 uL, 0.168 mmol), and DMF (0.5 mL) was stirred at RT for 1.5 hours. The reaction mixture was purified by reverse phase HPLC, 0-80% ACN/H_2O (TFA modifier) to give the title compound (4.9 mg, 14 %). ^{1}H NMR (500 MHz, DMSO-d_6) δ ppm 11.08 (s, 1H) 10.32 (s, 1H) 7.79 (dd, 1H) 7.70 (m, 2H), 7.56 (dd, 1H) 7.42 (t, 1H) 7.09 (dd, 1H) 6.93 (t, 1H) 6.43 (dd, 1H) 6.27 (dd, 1H) 5.78 (d, 1H) 3.08 (s, 3H) 2.63 (m, 1H) 1.27 (s, 3H) 0.96 (m, 2H) 0.68 (m, 2H) 3 protons masked by solvent; MS *m/z*: 627.82 $[M+1]^+$.

1-(3-Aminophenyl)-3-cyclopropyl-5-((2-fluoro-4-iodophenyl)amino)-6,8-dimethylpyrido[4,3-d]pyrimidine-2,4,7(1H,3H,6H)-trione was prepared according to the procedures in Abe et al (*67*).

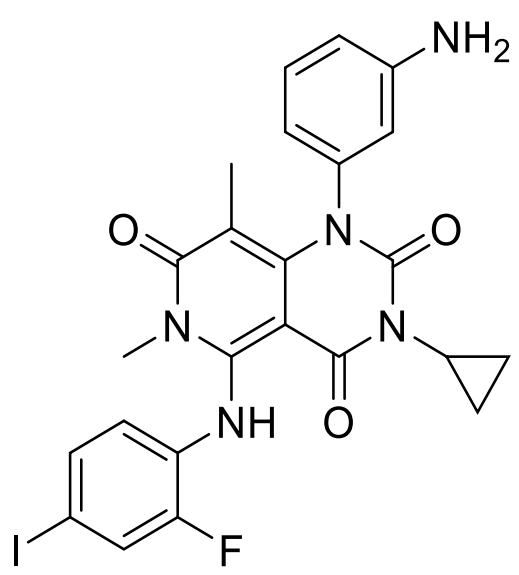

GD-01-012 *N*-(2-fluoro-3-((4-methyl-2-oxo-7-(pyrimidin-2-yloxy)-2H-chromen-3-yl)methyl)phenyl) acrylamide

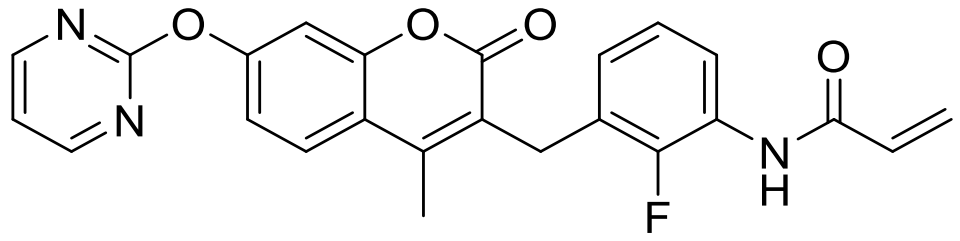

A solution of 3-(3-amino-2-fluorobenzyl)-4-methyl-7-(pyrimidin-2-yloxy)-2H-chromen-2-one (50 mg, 0.13 mmol), diisopropylethylamine (34.5 uL, 0.20 mmol), and DCM (2 mL) at 0°C was treated with acryloyl chloride (12.8 uL, 0.16 mmol). The reaction mixture was allowed to warm to room temperature and stirred for 15 minutes, then concentrated under reduced pressure. The residue was purified by reverse phase-HPLC, 0-80% ACN/H_2O (TFA modifier) to give the title compound (46 mg, 81%). ^{1}H NMR (500 MHz, DMSO-d_6) δ: 9.92 (s, 1H) 8.69 (d, 2H) 7.92 (d, 1H) 7.87 (m, 1H) 7.37 (d, 1H) 7.34 (t, 1H) 7.27 (dd, 1H) 7.06 (t, 1H) 6.96 (m, 1H) 6.62 (dd, 1H) 6.28 (dd, 1H) 5.78 (dd, 1H) 4.03 (s, 2H), 3 protons masked by solvent peak; MS *m/z*: 432.11 $[M+1]^+$.

3-(3-Amino-2-fluorobenzyl)-4-methyl-7-(pyrimidin-2-yloxy)-2H-chromen-2-one was prepared according to the procedures in Hyohdoh et al (*68*).

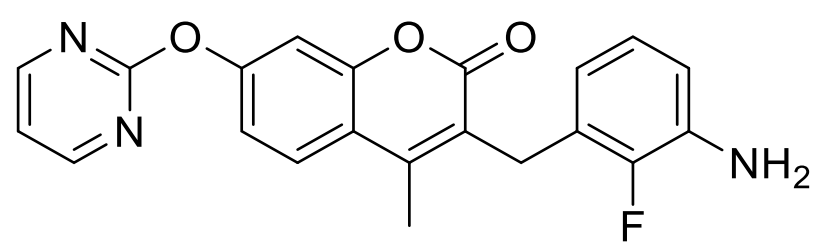

Procedures and Characterizations

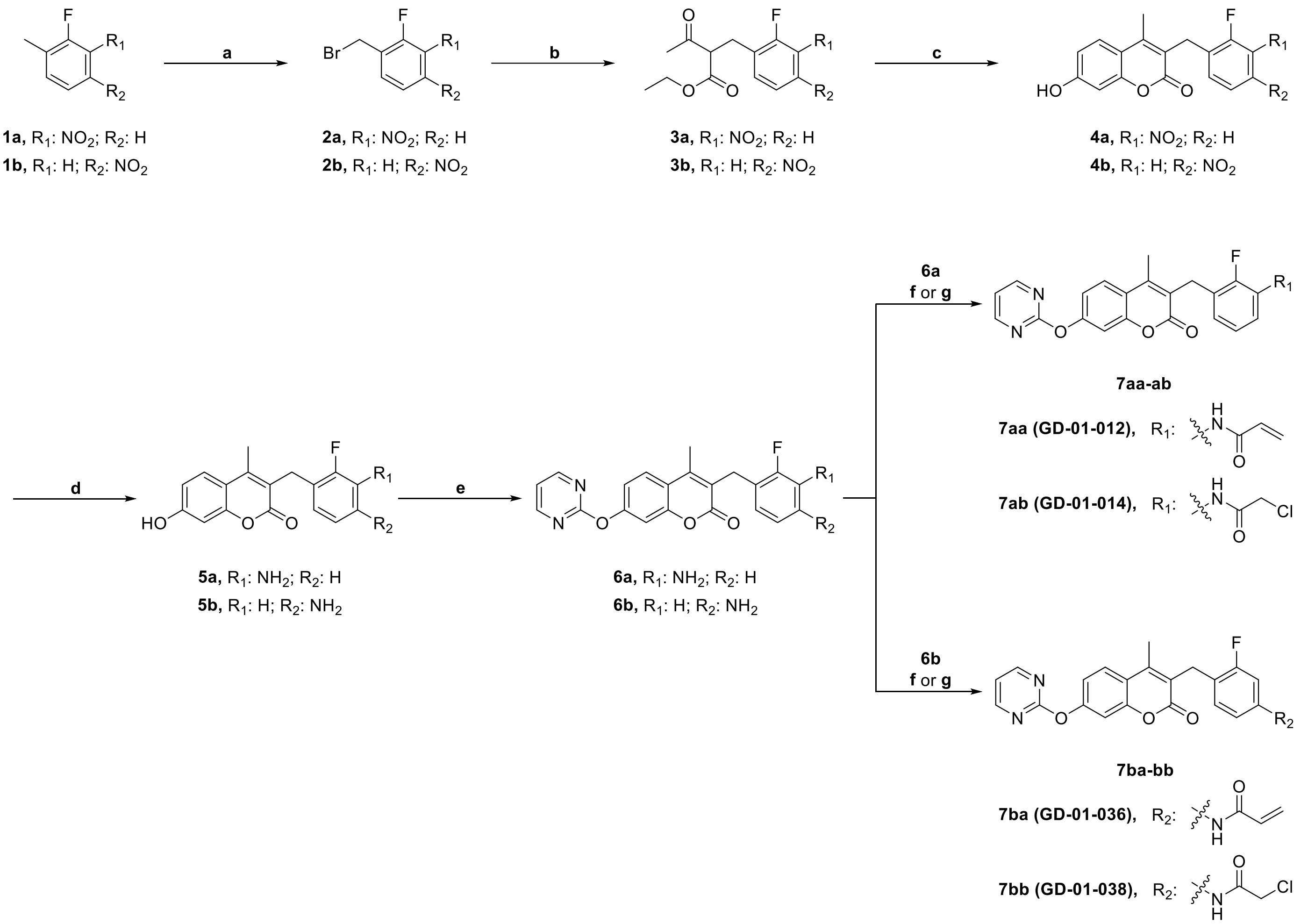

Fig. S6. Synthesis of **GD-01-012**, **GD-01-014**, **GD-01-036**, and **GD-01-038**. Reagents and conditions: (**a**) *N*-bromosuccinimide (NBS), azobisisobutyronitrile (AIBN), CCl_4, reflux, 16 h. (**b**) sodium hydride (NaH), ethylacetoacetate, dry THF, 0 °C to room temperature (RT), 16 h. (**c**) sulfuric acid, resorcinol, 0 °C to RT, 72 h. (**d**) tin(II) chloride dihydrate ($SnCl_2$•2 H_2O), EtOAc, reflux, 7 h. (**e**) 2-chloropyrimidine, Cs_2CO_3, DMF, 100 °C, 1 h. (**f**) acryloyl chloride, *N*,*N*-diisopropylethylamine (DIEA), DCM, 0 °C, 0.5 h. (**g**) chloroacetyl chloride, DIEA, DCM, 0 °C, 0.5 h.

<u>3-(3-amino-2-fluorobenzyl)-4-methyl-7-(pyrimidin-2-yloxy)-2*H*-chromen-2-one</u> (**6a**)[1]

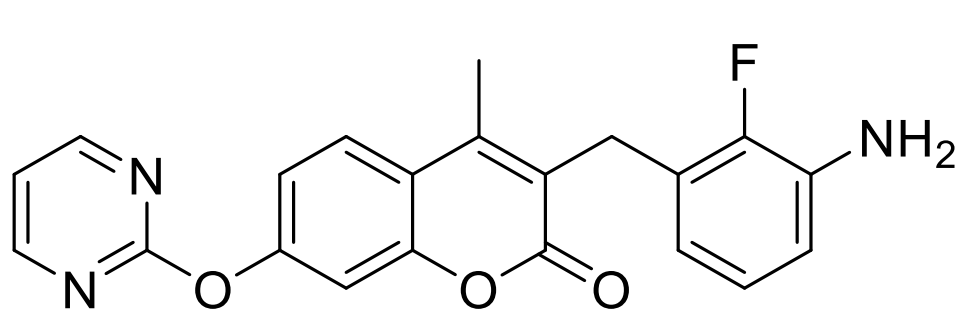

Intermediate **6a** and its precursors **2a-5a** were prepared according to procedures described previously by Hyohdoh and co-workers.[1] MS (*m/z*) ESI $[M+1]^+$ calc'd for $C_{21}H_{16}FN_3O_3$: 378.12, found 378.06.[1]

N-(2-Fluoro-3-((4-methyl-2-oxo-7-(pyrimidin-2-yloxy)-2*H*-chromen-3-yl)methyl)phenyl)acrylamide (**7aa**, **GD-01-012**)

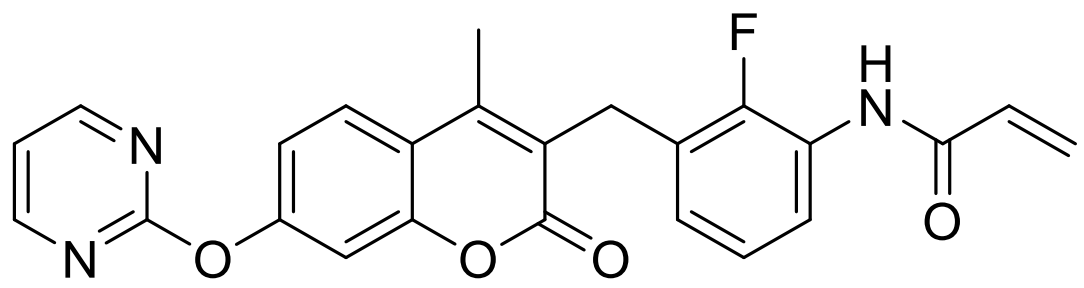

General Procedure A: A solution of **6a** (50 mg, 0.13 mmol), *N*,*N*-diisopropylethylamine (34.5 µL, 0.20 mmol), and DCM (2 mL) at 0 °C was treated with acryloyl chloride (12.8 µL, 0.16 mmol). The reaction mixture was allowed to warm to room temperature (RT) and stirred for 0.5 h, then concentrated under reduced pressure. The residue was purified by reverse phase-HPLC, 0–80% ACN/H_2O (0.035% TFA modifier) to give the title compound (46 mg, 81%). ^{1}H NMR (500 MHz, DMSO-d_6) δ 9.92 (s, 1H), 8.69 (d, J = 4.8 Hz, 2H), 7.92 (d, J = 8.8 Hz, 1H), 7.87 (t, J = 7.2 Hz, 1H), 7.38 (d, J = 2.4 Hz, 1H), 7.34 (t, J = 4.8 Hz, 1H), 7.27 (dd, J = 8.7, 2.4 Hz, 1H), 7.06 (t, J = 7.9 Hz, 1H), 6.96 (t, J = 6.8 Hz, 1H), 6.62 (dd, J = 17.0, 10.2 Hz, 1H), 6.27 (dd, J = 17.0, 1.9 Hz, 1H), 5.78 (d, J = 1.9 Hz, 1H), 2.49 (s, 3H, 3 protons masked by solvent peak). ^{13}C NMR (125 MHz, DMSO) δ 166.50, 166.48, 163.0, 160.4, 156.7, 155.4, 153.4, 144.8, 127.9, 127.8, 126.7, 126.2, 126.0, 124.5, 123.2, 122.8, 121.0, 120.5, 116.1, 115.5, 104.9, 32.3, 17.9. MS (*m/z*) ESI $[M+1]^+$ calc'd for $C_{24}H_{18}FN_3O_4$: 432.14, found 432.07.

1-(bromomethyl)-2-fluoro-4-nitrobenzene (**2b**)

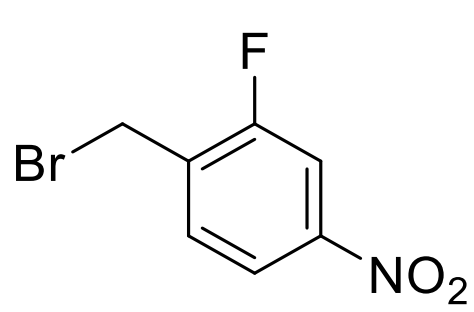

A solution of 2-fluoro-1-methyl-4-nitrobenzene (2.4 g, 15 mmol), *N*-bromosuccinimide (3.2 g, 18 mmol), and azobisisobutyronitrile (0.25 g, 1.5 mmol) in 50 mL of CCl_4 was heated to reflux for 16 h. After cooling, the mixture was filtered. The filtrate was concentrated under reduced pressure and the residue purified by flash chromatography, 0–20% EtOAc/hexane over 33 minutes. Fractions containing product were combined and concentrated under reduced pressure to afford the title compound (2.07 g, 59%). ^{1}H NMR (500 MHz, $CDCl_3$) δ 8.07 – 8.00 (m, 1H) 7.77 – 7.71 (m, 1H) 7.36 – 7.29 (m, 1H) 4.63 – 4.54 (m, 2H).

Ethyl 2-(2-fluoro-4-nitrobenzyl)-3-oxobutanoate (**3b**)

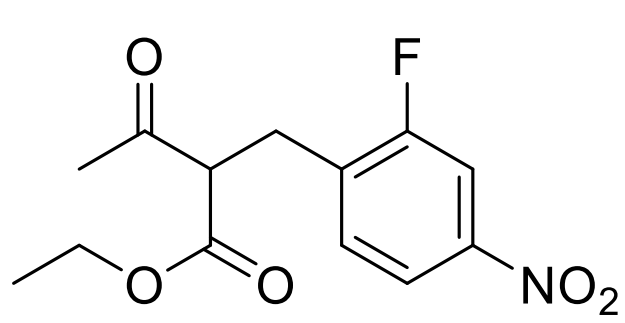

A suspension of sodium hydride (0.33 g, 65% in oil, 8.8 mmol) in anhydrous THF (20 mL) under N_2 was cooled to 0 °C in an ice bath. Ethyl acetoacetate (1.2 mL, 8.8 mmol) was added, and the mixture stirred at RT for 0.5 h. A solution of 1-(bromomethyl)-2-fluoro-4-nitrobenzene (2.07 g, 8.8 mmol) in 15 mL of THF was added. Cooling

was removed and the mixture was stirred 16 h at RT. The resulting mixture was treated with 50 mL of 2 N HCl and extracted with 3 x 75 mL of EtOAc. The combined organic extracts were washed consecutively with water and brine, dried over Na_2SO_4, filtered and concentrated under reduced pressure. The residue was purified by flash chromatography, 0–30% EtOAc/hexane over 33 minutes. Like fractions containing product were combined and concentrated under reduced pressure to give the title compound (1.54 g, 61%). ^{1}H NMR (500 MHz, $CDCl_3$) δ 7.99 – 7.87 (m, 1 H) 7.62 – 7.52 (m, 1 H) 7.25 – 7.15 (m, 1 H) 4.26 – 4.10 (m, 2 H) 3.93 – 3.80 (m, 1 H) 3.41 – 3.14 (m, 2 H) 2.33 – 2.25 (m, 3 H) 1.30 – 1.15 (m, 3 H).

<u>3-(2-Fluoro-4-nitrobenzyl)-7-hydroxy-4-methyl-2*H*-chromen-2-one</u> (**4b**)

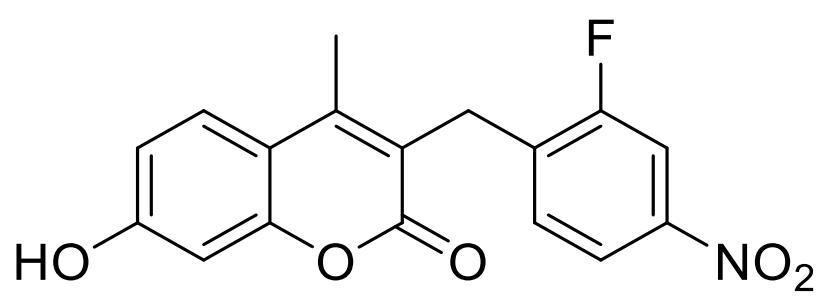

Ethyl 2-(2-fluoro-4-nitrobenzyl)-3-oxobutanoate (1.5 g, 4.55 mmol) in 3 mL of sulfuric acid at 0 °C was treated with resorcinol (0.5 g, 4.55 mmol). The mixture was stirred for 72 h at RT and quenched with 30 mL of ice water. The resulting precipitate was filtered, washed with water and dried to give the title compound (1.18 g, 78.6 %). ^{1}H NMR (500 MHz, DMSO-d_6) δ 10.56 – 10.38 (m, 1 H) 8.07 – 7.92 (m, 1 H) 7.80 – 7.66 (m, 1 H) 7.61 – 7.50 (m, 1 H) 7.37 – 7.26 (m, 1 H) 6.98 – 6.79 (m, 1 H) 6.79 – 6.66 (m, 1 H) 4.13 – 3.94 (m, 2 H) 2.45 – 2.40 (m, 3 H). MS (*m/z*) ESI $[M+1]^+$ calc'd for $C_{17}H_{12}FNO_5$: 330.14, found 330.10.

<u>3-(4-Amino-2-fluorobenzyl)-7-hydroxy-4-methyl-2*H*-chromen-2-one</u> (**5b**)

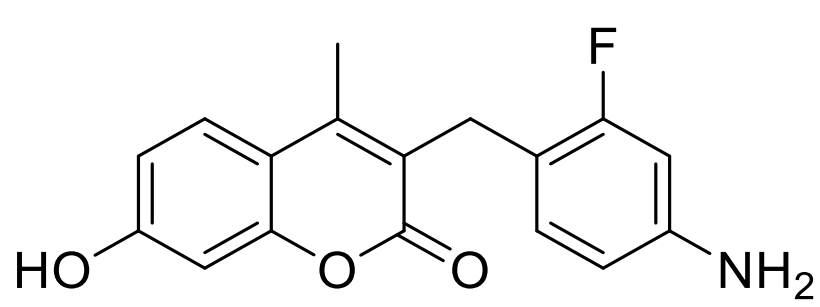

A mixture of 3-(2-fluoro-4-nitrobenzyl)-7-hydroxy-4-methyl-2*H*-chromen-2-one (1.17g, 3.55 mmol), tin (II) chloride dihydrate (4.01 g, 17.76 mmol), and EtOAc (50 mL) was heated to reflux for 7 h. After cooling, the mixture was quenched with 30 mL of saturated $NaHCO_3$ and extracted with EtOAc (2 x 50 mL). The combined organic extracts were washed with brine, dried over Na_2SO_4, filtered and concentrated under reduced pressure to give the title compound (0.48 g, 45 %). ^{1}H NMR (500 MHz, DMSO-d_6) δ 10.36 – 10.49 (m, 1 H) 7.57 – 7.73 (m, 1 H) 6.78 – 6.87 (m, 1 H) 6.65 – 6.78 (m, 2 H) 6.53 – 6.65 (m, 1 H) 6.15 – 6.31 (m, 1 H) 4.97 – 5.12 (m, 2 H) 3.73 – 3.92 (m, 2 H) 2.31 – 2.41 (m, 3 H). MS (*m/z*) ESI $[M+1]^+$ calc'd for $C_{17}H_{14}FNO_3$: 330.14, found 330.28.

<u>3-(4-Amino-2-fluorobenzyl)-4-methyl-7-(pyrimidin-2-yloxy)-2H-chromen-2-one</u> (**6b**)

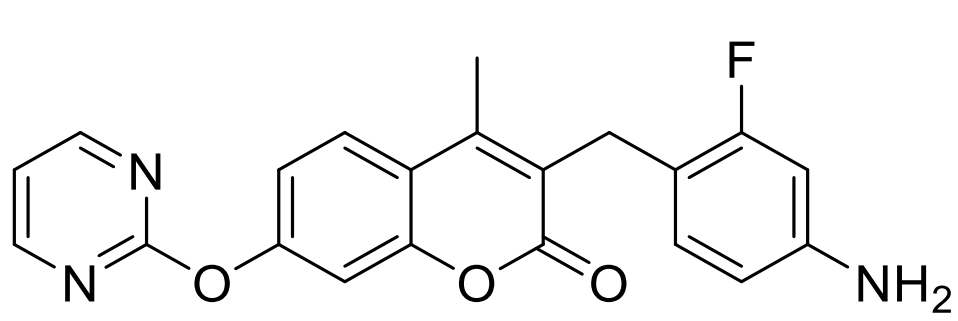

A mixture of 3-(4-amino-2-fluorobenzyl)-7-hydroxy-4-methyl-2H-chromen-2-one (0.48 g, 1.6 mmol), 2-chloropyrimidine (0.73 g, 604 mmol), Cs_2CO_3 (0.63 mg, 1.9 mmol) and DMF (15 mL), was heated to 100 °C for 1 h. After cooling, the reaction mixture was poured into 150 mL of water and extracted with 3 x 100 mL of EtOAc. The combined organic extracts were washed with brine, dried over Na_2SO_4,, filtered and concentrated under reduced pressure. The residue was purified by flash chromatography eluting with 0–100 % EtOAc/hexane over 33 minutes. Like fractions were pooled and concentrated under reduced pressure to give the title compound (0.18 g, 30 %). ^{1}H NMR (500 MHz, DMSO-d_6) δ 8.73 – 8.64 (m, 2 H) 7.88 (d, J = 8.80 Hz, 1 H) 7.41 – 7.30 (m, 2 H) 7.30 – 7.20 (m, 1 H) 6.78 (t, J = 8.62 Hz, 1 H) 6.21 – 6.39 (m, 2 H) 5.15 – 5.26 (m, 2 H) 3.76 – 3.82 (m, 2 H) 2.41 – 2.47 (m, 3 H). MS (*m/z*) ESI $[M+1]^+$ calc'd for $C_{21}H_{16}FN_3O_3$: 330.12, found 378.24.

2-chloro-*N*-(2-fluoro-3-((4-methyl-2-oxo-7-(pyrimidin-2-yloxy)-2*H*-chromen-3-yl)methyl)phenyl)acetamide (**7ab**, **GD-01-014**)

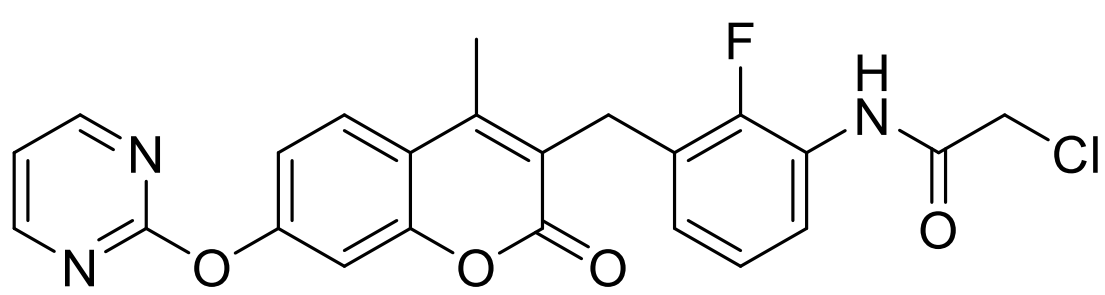

The final compound **7ab** (**GD-01-014**) was prepared as described for **7aa** (**GD-01-012**), following **General Procedure A**, from **6a** and substituting acryloyl chloride by chloroacetyl chloride. The crude was purified by reverse phase-HPLC, 0–80% ACN/H_2O (0.035% TFA modifier) to give the title compound (66 mg, 72%). ^{1}H NMR (500 MHz, DMSO-d_6) δ 10.07 (s, 1H), 8.69 (d, J = 4.8 Hz, 2H), 7.92 (d, J = 8.8 Hz, 1H), 7.75 (t, J = 7.4 Hz, 1H), 7.38 (d, J = 2.3 Hz, 1H), 7.34 (t, J = 4.8 Hz, 1H), 7.27 (dd, J = 8.7, 2.4 Hz, 1H), 7.07 (t, J = 7.9 Hz, 1H), 6.98 (t, J = 6.9 Hz, 1H), 4.35 (s, 2H), 4.03 (s, 2H), 2.49 (s, 3H, 3 protons masked by solvent peak). MS (*m/z*) ESI $[M+1]^+$ calc'd for $C_{23}H_{17}ClFN_3O_4$: 454.10, found 454.04.

N-(3-fluoro-4-((4-methyl-2-oxo-7-(pyrimidin-2-yloxy)-2*H*-chromen-3-yl)methyl)phenyl)acrylamide (**7ba**, **GD-01-036**)

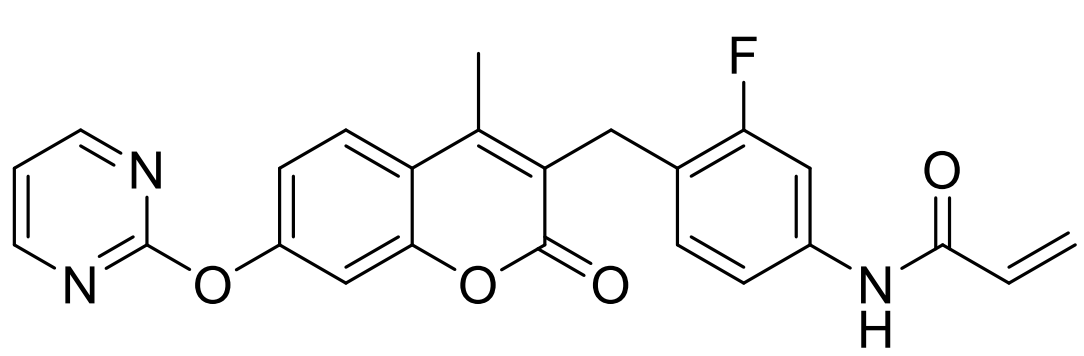

The final compound **7ba** (**GD-01-036**) was prepared as described for **7aa** (**GD-01-012**), following **General Procedure A**, substituting **6a** by **6b**. The crude was purified by reverse phase-HPLC, 0–80% ACN/H_2O (0.035% TFA modifier) over 30 minutes to give the title compound (42 mg, 74%). ^{1}H NMR (500 MHz, DMSO-d_6) δ 10.29 (s, 1H), 8.69 (d, J = 4.8 Hz, 2H), 7.91 (d, J = 8.8 Hz, 1H), 7.70 (dd, J = 12.7, 1.9 Hz, 1H), 7.38 (d, J = 2.4 Hz, 1H), 7.34 (t, J = 4.8 Hz, 1H), 7.27 (dd, J = 8.7, 2.4 Hz, 1H), 7.22 (dd, J = 8.4, 2.0 Hz, 1H), 7.12 (t, J = 8.6 Hz, 1H), 6.40 (dd, J = 17.0, 10.1 Hz, 1H), 6.26 (dd, J = 17.0, 2.0 Hz, 1H), 5.77 (dd, J = 10.1, 2.0 Hz, 1H), 3.95 (s, 2H), 2.48 (s, 3H). MS (*m/z*) ESI $[M+1]^+$ calc'd for $C_{24}H_{18}FN_3O_4$: 432.14, found 432.06.

2-chloro-*N*-(3-fluoro-4-((4-methyl-2-oxo-7-(pyrimidin-2-yloxy)-2*H*-chromen-3-yl)methyl)phenyl)acetamide (**7bb**, **GD-01-038**)

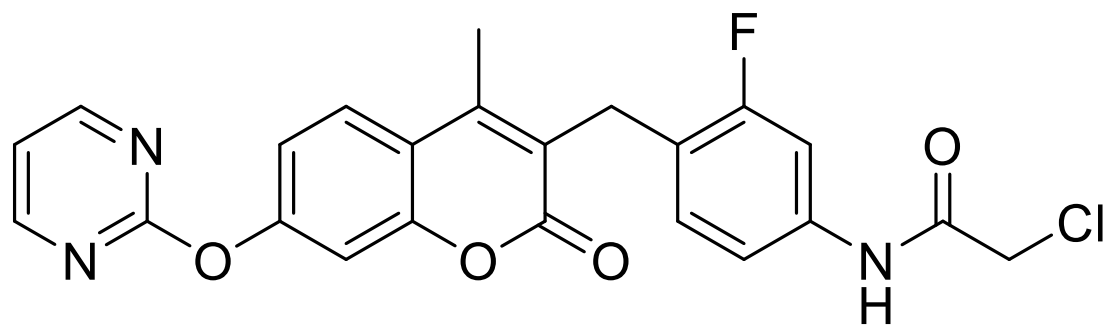

The final compound **7bb** (**GD-01-038**) was prepared as described for **7aa** (**GD-01-012**), following **General Procedure A**, from **6b** and substituting acryloyl chloride by chloroacetyl chloride. The crude was purified by reverse phase-HPLC, 0–80% ACN/H_2O (0.035% TFA modifier) to give the title compound (42 mg, 56%). ^1H NMR (500 MHz, DMSO-d_6) δ 10.45 (s, 1H), 8.69 (d, J = 4.8 Hz, 2H), 7.91 (d, J = 8.8 Hz, 1H), 7.59 (dd, J = 12.5, 1.8 Hz, 1H), 7.38 (d, J = 2.3 Hz, 1H), 7.34 (t, J = 4.8 Hz, 1H), 7.27 (dd, J = 8.7, 2.4 Hz, 1H), 7.18 (dd, J = 8.4, 1.9 Hz, 1H), 7.13 (t, J = 8.4 Hz, 1H), 4.25 (s, 2H), 3.95 (s, 2H), 2.48 (s, 3H). MS (*m/z*) ESI $[M+1]^+$ calc'd for $C_{23}H_{17}ClFN_3O_4$: 454.10, found 454.05.

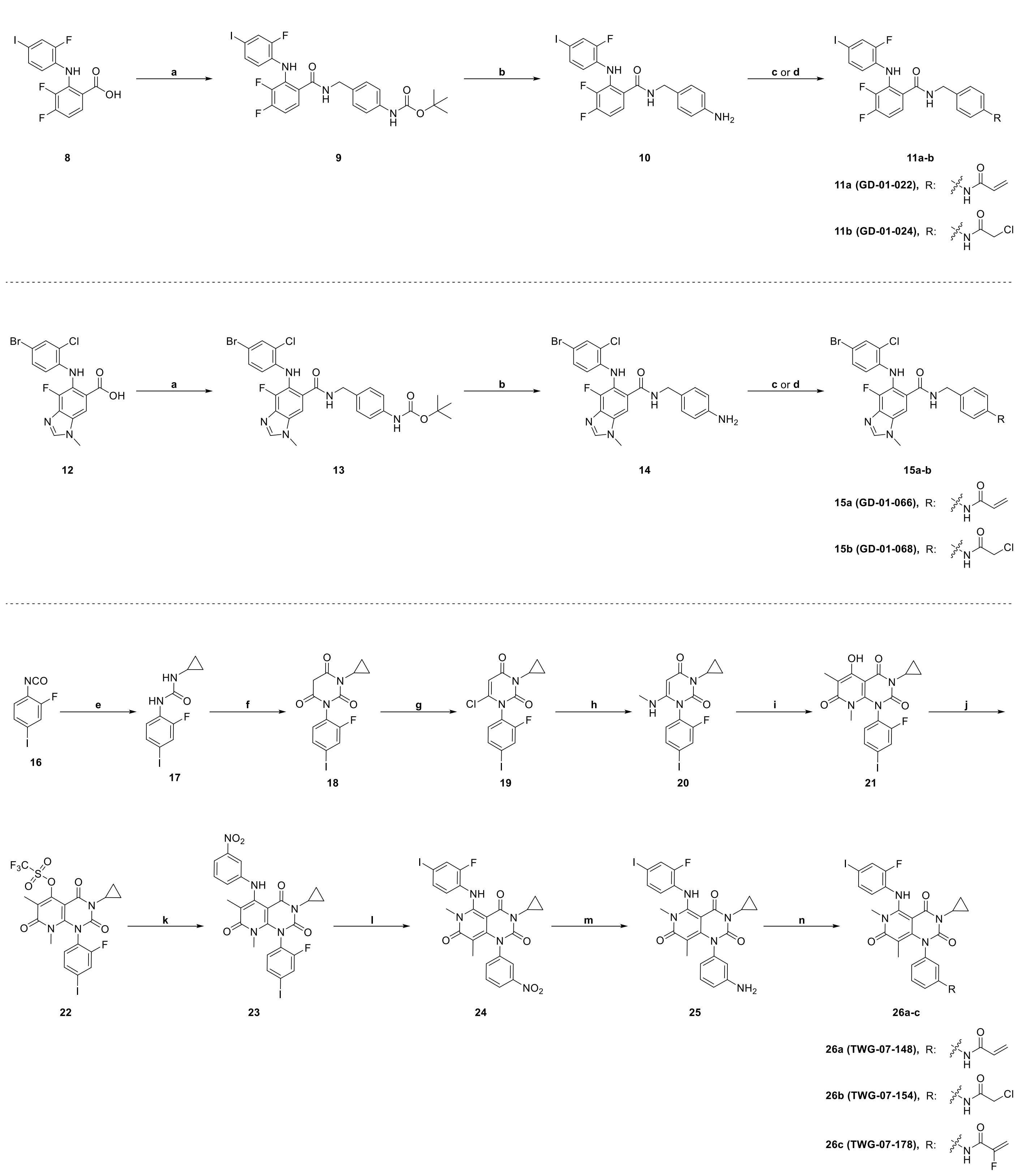

Fig. S7. Synthesis of **GD-01-022**, **GD-01-024**, **GD-01-066**, **GD-01-068**, and for compounds **TWG-07-148**, **TWG-07-154**, and **TWG-07-178**. Reagents and conditions: (**a**) *tert*-butyl (4-(aminomethyl)phenyl)carbamate, DIEA, HATU, DMF, RT, 1 h. (**b**) trifluoroacetic acid, DCM, RT, 0.5 h. (**c**) acryloyl chloride, DIEA, DCM, 0 °C, 0.5 h. (**d**) chloroacetyl chloride, DIEA, DCM, 0 °C, 0.5 h. (**e**) cyclopropylamine, Et_2O, RT, 2 h. (**f**) malonic acid, acetic anhydride, 100 °C, 6 h. (**g**) phosphorus oxychloride ($POCl_3$), H_2O, 90 °C, 3 h. (**h**) methyl amine, EtOH, 80 °C, 2 h. (**i**) diethylmalonate, diphenyl ether (Ph_2O), 220 °C, 5 h. (**j**) trifluoromethanesulfonic anhydride ($(CF_3SO_2)_2O$), 2,6-lutidine, $CHCl_3$, RT, 2 h. (**k**) 3-nitroaniline, neat, 130 °C, 2 h. (**l**) K_2CO_3, MeOH/THF (1:2), 80 °C, 2 h. (**m**) sodium hydrosulfite ($Na_2S_2O_4$), DMF, 90 °C, 1 h. (**n**) carboxylic acid, DIEA, HATU, DMF, RT, 1 h.

tert-Butyl (4-((3,4-difluoro-2-((2-fluoro-4-iodophenyl)amino)benzamido)methyl)phenyl)carbamate (**9**)

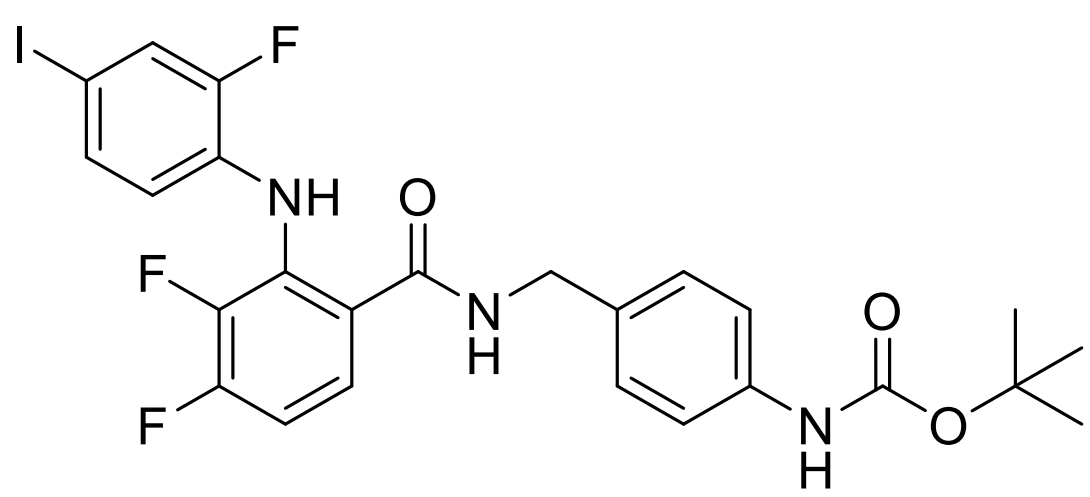

General Procedure B: A mixture of 3,4-difluoro-2-((2-fluoro-4-iodophenyl)amino)benzoic acid (**8**, 0.5 g, 1.27 mmol), *tert*-butyl (4-(aminomethyl)phenyl)carbamate (0.489 g, 2.20 mmol), DIEA (333 µL, 1.9 mmol), HATU (0.967 g, 2.54 mmol), and DMF (4 mL) was stirred at RT for 1 h. The reaction mixture was poured into 50 mL of ice water and extracted with 2 x 50 mL of EtOAc. The combined organic extracts were washed with brine, dried over Na_2SO_4,, filtered and concentrated under reduced pressure. The crude material was purified by flash chromatography eluting with 0-50% EtOAc/hexane over 20 minutes. Tubes containing product were pooled and concentrated under reduced pressure to give the title compound (0.558 g, 73.5%). ^{1}H NMR (500 MHz, DMSO-d_6) δ 9.34 – 9.24 (m, 2H) 9.24 – 9.11 (m, 1H) 7.67 – 7.53 (m, 2H) 7.47 – 7.31 (m, 3H) 7.26 – 7.07 (m, 3H) 6.80 – 6.58 (m, 1H) 4.51 – 4.27 (m, 2H) 1.47 (s, 9H). MS (*m/z*) ESI $[M+1]^+$ calc'd for $C_{25}H_{23}F_3IN_3O_3$: 598.08, found 598.37.

N-(4-Aminobenzyl)-3,4-difluoro-2-((2-fluoro-4-iodophenyl)amino)benzamide (**10**)

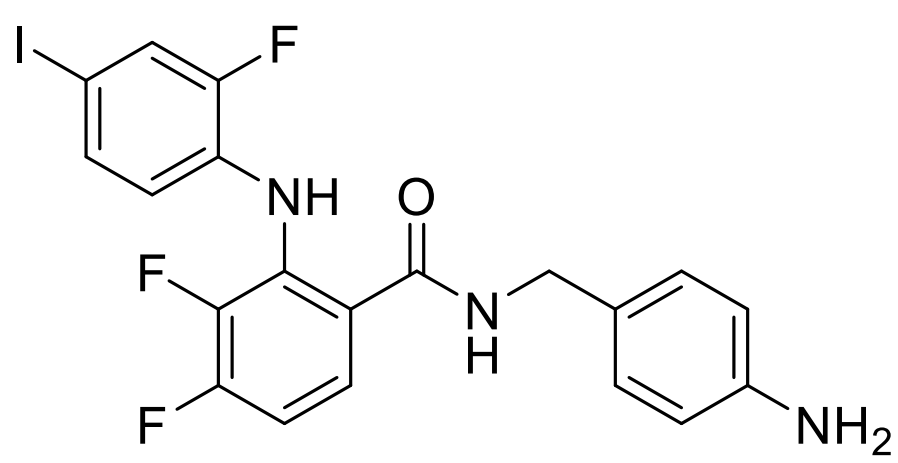

General Procedure C: A solution of **9** (0.36 g, 0.6 mmol) in 1 mL of DCM was treated with 1 mL of trifluoroacetic acid (TFA). After 0.5 h, the reaction was deemed complete by LCMS. Excess solvent and reagent were removed under reduced pressure to give a quantitative yield of the title compound (0.367 g, TFA salt). ^{1}H NMR (500 MHz, DMSO-d_6) δ 9.39 – 9.21 (m, 2H) 7.66 – 7.58 (m, 2H) 7.42 – 7.35 (m, 1H) 7.33 – 7.26 (m, 2H) 7.26 – 7.15 (m, 1H) 7.14 – 6.96 (m, 2H) 6.76 – 6.62 (m, 1H) 4.44 – 4.35 (m, 2H). MS (*m/z*) ESI $[M+1]^+$ calc'd for $C_{20}H_{15}F_3IN_3O$: 498.03, found 498.23.

N-(3-acrylamidobenzyl)-3,4-difluoro-2-((2-fluoro-4-iodophenyl)amino)benzamide (**11a**, **GD-01-022**)

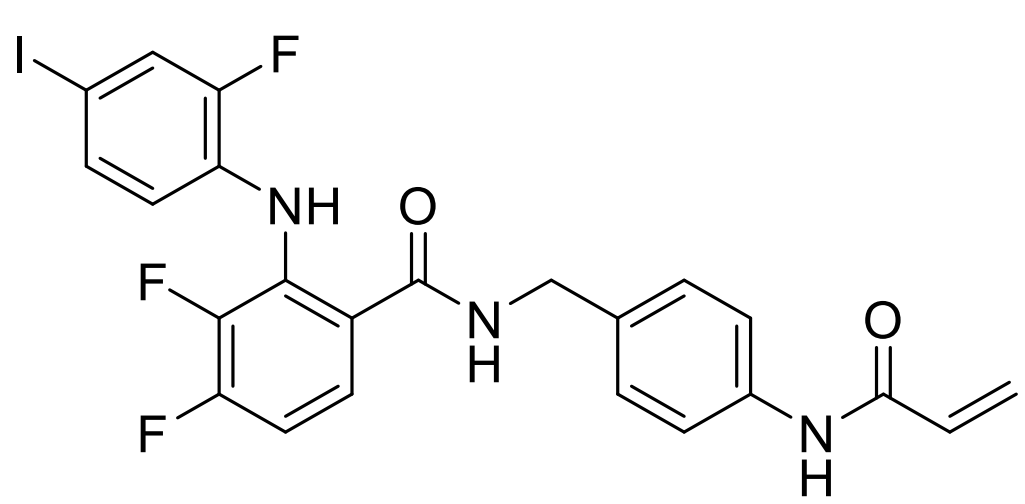

The final compound **11a** (**GD-01-022**) was prepared as described for **7aa** (**GD-01-012**), following **General Procedure A**, substituting **6a** by **10**. The crude was purified by reverse phase-HPLC, 0–80% ACN/H_2O (0.035% TFA modifier) over 35 minutes to give the title compound (51 mg, 68%). ^{1}H NMR (500 MHz, DMSO-*d*6) *δ* 10.11 (s, 1H), 9.27 (s, 1H), 9.23 (t, *J* = 5.9 Hz, 1H), 7.64 – 7.57 (m, 4H), 7.39 (dd, *J* = 8.5, 1.3 Hz, 1H), 7.25 (d, *J* = 8.6 Hz, 2H), 7.23 – 7.18 (m, 1H), 6.71 (td, *J* = 8.9, 5.2 Hz, 1H), 6.42 (dd, *J* = 17.0, 10.1 Hz, 1H), 6.25 (dd, *J* = 17.0, 2.0 Hz, 1H), 5.74 (dd, *J* = 10.1, 2.0 Hz, 1H), 4.38 (d, *J* = 5.8 Hz, 2H). MS (*m/z*) ESI $[M+1]^+$ calc'd for $C_{23}H_{17}F_3IN_3O_2$: 552.04, found 551.98.

N-(3-(2-chloroacetamido)benzyl)-3,4-difluoro-2-((2-fluoro-4-iodophenyl)amino)benzamide (**11b**, **GD-01-024**)

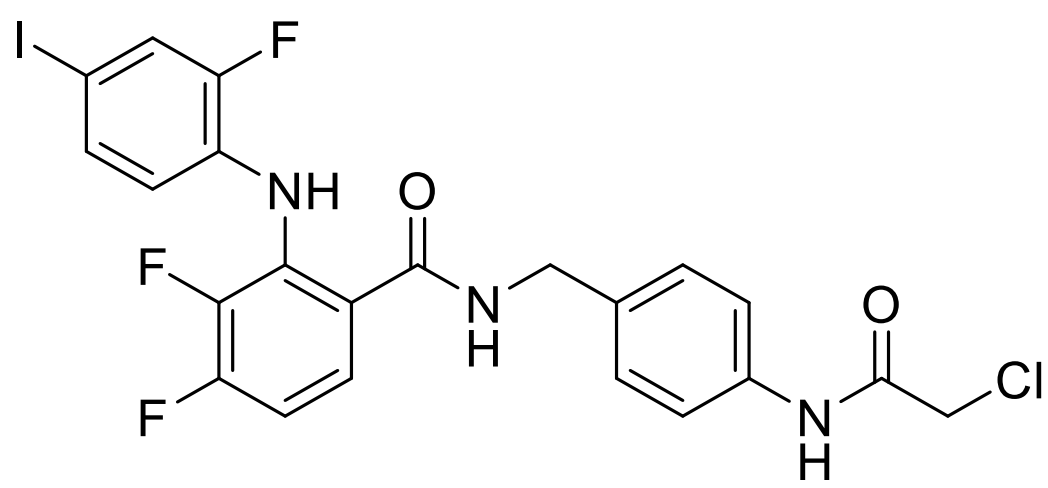

Compound **11b** (**GD-01-024**) was prepared as described for **7aa** (**GD-01-012**), following **General Procedure A**, substituting **6a** by **10**, and acryloyl chloride by chloroacetyl chloride. The crude was purified by reverse phase-HPLC, 0–80% ACN/H_2O (0.035% TFA modifier) over 35 minutes to give the title compound (62 mg, 71%). ^{1}H NMR (500 MHz, DMSO-*d*6) *δ* 10.27 (s, 1H), 9.26 (s, 1H), 9.23 (t, *J* = 5.8 Hz, 1H), 7.64 – 7.61 (m, 1H), 7.59 (dd, *J* = 10.8, 1.9 Hz, 2H), 7.52 (d, *J* = 8.5 Hz, 2H), 7.38 (dd, *J* = 8.4, 1.3 Hz, 1H), 7.26 (d, *J* = 8.6 Hz, 2H), 7.21 (td, *J* = 9.3, 7.5 Hz, 1H), 6.71 (td, *J* = 8.9, 5.1 Hz, 1H), 4.39 (d, *J* = 5.8 Hz, 2H), 4.24 (s, 2H). MS (*m/z*) ESI $[M+1]^+$ calc'd for $C_{22}H_{16}ClF_3IN_3O_2$: 574.00, found 573.95.

Methyl 5-((4-bromo-2-chlorophenyl)amino)-1-(4-((tert-butoxycarbonyl)amino)benzyl)-4-fluoro-1*H*-benzo[*d*]imidazole-6-carboxylate (**13**)

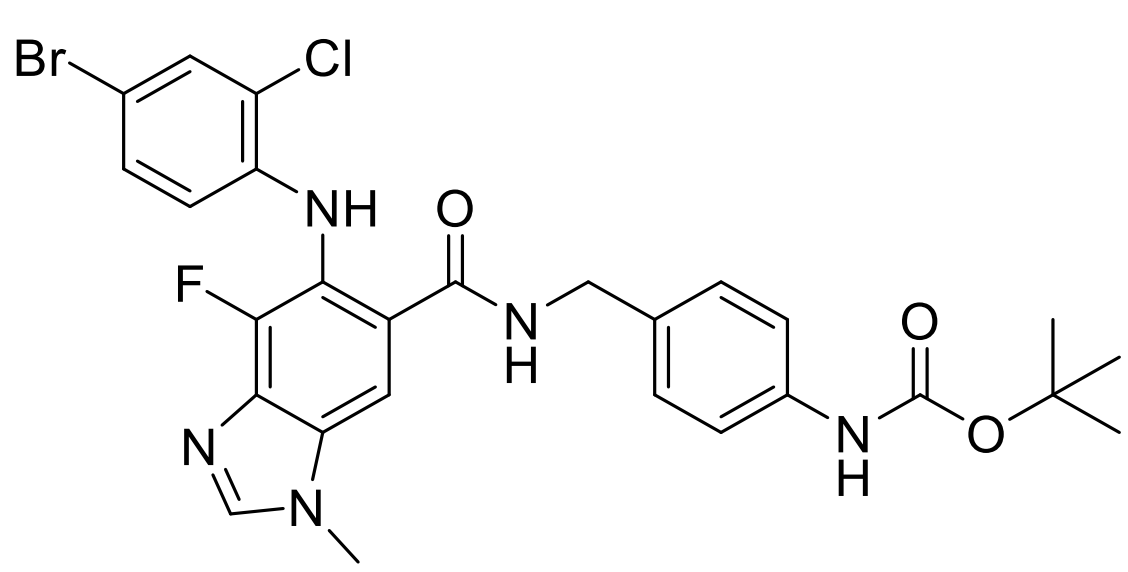

Intermediate **13** was prepared as described for **9** following **General Procedure B**, substituting **8** by methyl 5-((4-bromo-2-chlorophenyl)amino)-4-fluoro-1*H*-benzo[*d*]imidazole-6-carboxylate (**12**, 0.272 g, 0.68 mmol). The mixture was purified by reverse phase-HPLC, 0-80% ACN/H_2O (0.035% TFA modifier) over 1 h. Like fractions containing product were pooled and lyophilized to give the title compound (80 mg, 20%). ^{1}H NMR (500 MHz, DMSO-*d*6) *δ* 9.42 – 9.34 (m, 1H) 8.70 – 8.60 (m, 1H) 8.14 – 8.07 (m, 1H) 8.07 – 7.96 (m, 1H) 7.70 – 7.59 (m, 1H) 7.51 – 7.38 (m, 2H) 7.31 – 7.17 (m, 3H) 6.51 – 6.37 (m, 1H) 5.59 – 5.48 (m, 2H) 3.83 (s, 3H) 1.54 (s, 9H). MS (*m/z*) ESI $[M+1]^+$ calc'd for $C_{27}H_{26}BrClFN_5O_3$: 602.10, found 602.21.

Methyl 1-(4-aminobenzyl)-5-((4-bromo-2-chlorophenyl)amino)-4-fluoro-1*H*-benzo[*d*]imidazole-6-carboxylate (**14**)

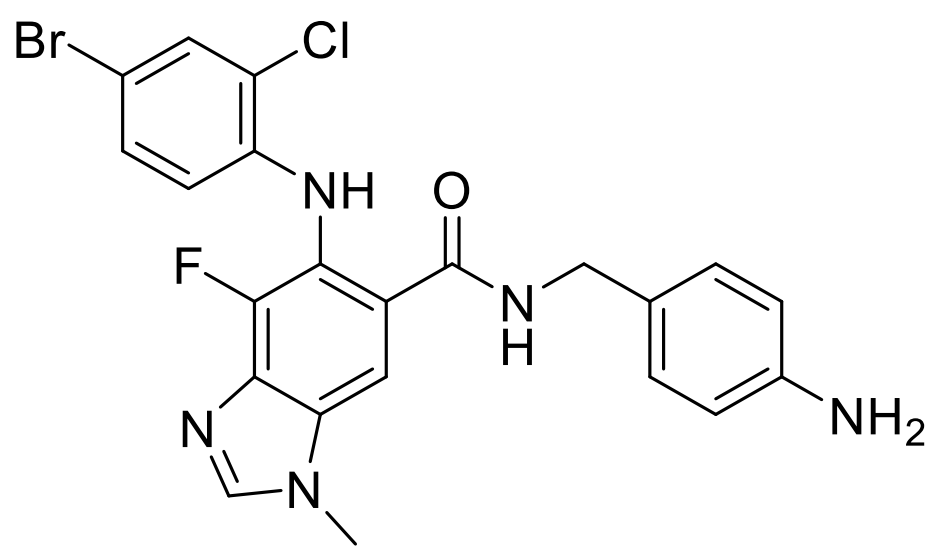

Intermediate **14** was prepared as described for **10** following **General Procedure C**, substituting **9** by methyl 5-((4-bromo-2-chlorophenyl)amino)-1-(4-((*tert*-butoxycarbonyl)amino)benzyl)-4-fluoro-1*H*-benzo[*d*]imidazole-6-carboxylate (**13**, 48 mg, 0.08 mmol). After 0.5 h, the reaction was deemed complete by LCMS. Excess solvent and reagent were removed under reduced pressure to give a quantitative yield of the title compound in quantitative yields (40 mg). MS (*m/z*) ESI $[M+1]^+$ calc'd for $C_{22}H_{18}BrClFN_5O$: 502.04, found 502.18.

N-(4-acrylamidobenzyl)-5-((4-bromo-2-chlorophenyl)amino)-4-fluoro-1-methyl-1*H*-benzo[*d*]imidazole-6-carboxamide (**15a**, **GD-01-066**)

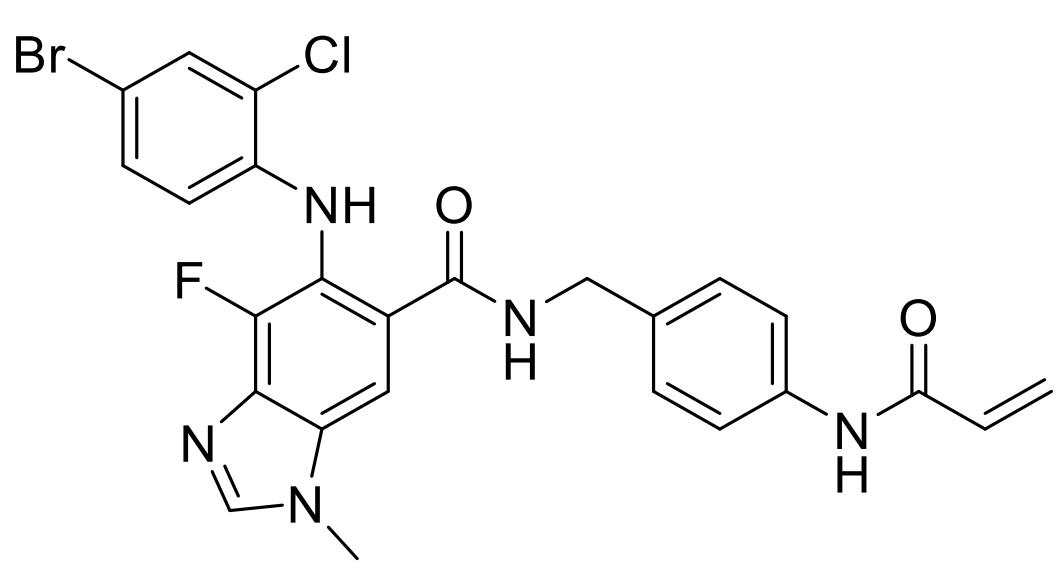

The title compound **15a** (**GD-01-066**) was prepared as described for **7aa** (**GD-01-012**), following **General Procedure A**, substituting **6a** by **14**. The crude was purified by reverse phase-HPLC, 0–80% ACN/H_2O (0.035% TFA modifier) over 33 minutes to give the title compound (42 mg, 51%). ^1H NMR (500 MHz, DMSO-d_6) δ 10.11 (s, 1H), 9.10 (t, J = 5.9 Hz, 1H), 8.41 (d, J = 2.1 Hz, 2H), 7.87 (s, 1H), 7.61 (d, J = 2.3 Hz, 1H), 7.58 (d, J = 8.5 Hz, 2H), 7.28 (dd, J = 8.8, 2.3 Hz, 1H), 7.24 (d, J = 8.5 Hz, 2H), 6.47 – 6.38 (m, 2H), 6.25 (dd, J = 17.0, 2.0 Hz, 1H), 5.80 – 5.70 (m, 1H), 4.41 (d, J = 5.8 Hz, 2H), 3.92 (s, 3H). MS (*m/z*) ESI $[M+1]^+$ calc'd for Chemical Formula: $C_{25}H_{20}BrClFN_5O_2$: 556.05, found 556.00.

5-((4-bromo-2-chlorophenyl)amino)-*N*-(4-(2-chloroacetamido)benzyl)-4-fluoro-1-methyl-1*H*-benzo[*d*]imidazole-6-carboxamide (**15b**, **GD-01-068**)

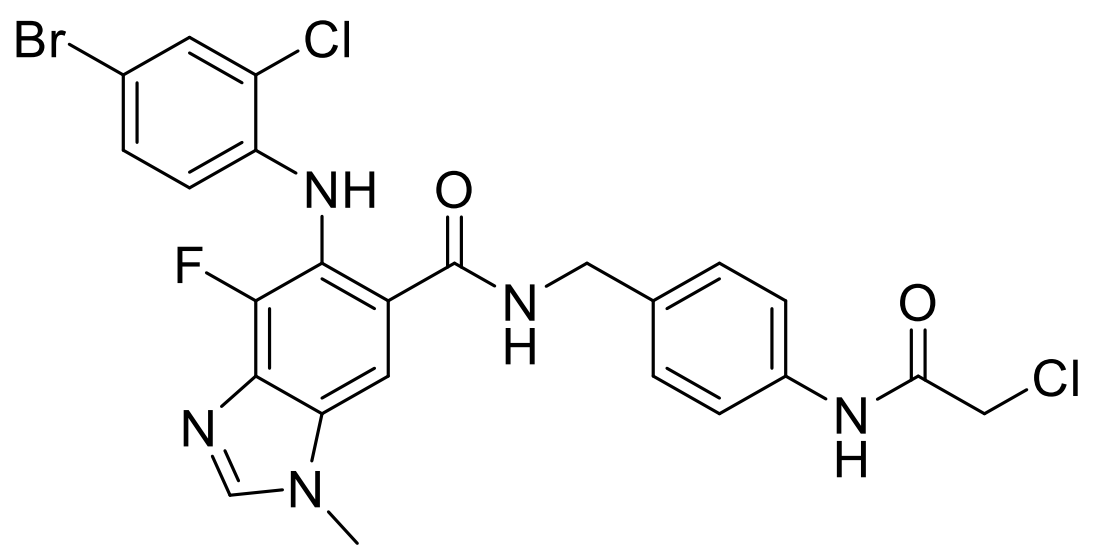

Compound **15b** (**GD-01-068**) was prepared as described for **7aa** (**GD-01-012**), following **General Procedure A**, substituting **6a** by **14**, and acryloyl chloride by chloroacetyl chloride. The crude was purified by reverse phase-HPLC, 0–80% ACN/H_2O (0.035% TFA modifier) over 33 minutes to give the title compound (57 mg, 55%). ^1H NMR (500 MHz, DMSO-d_6) δ 10.26 (s, 1H), 9.10 (t, J = 5.9 Hz, 1H), 8.40 (s, 1H), 8.38 (s, 1H), 7.86 (s, 1H), 7.61 (d, J = 2.3 Hz, 1H), 7.50 (d, J = 8.5 Hz, 2H), 7.27 (dd, J = 8.8, 2.3 Hz, 1H), 7.24 (d, J = 8.5 Hz, 2H), 6.40 (dd, J = 8.8, 4.7 Hz, 1H), 4.41 (d, J = 5.8 Hz, 2H), 4.24 (s, 2H), 3.91 (s, 3H). MS (*m/z*) ESI $[M+1]^+$ calc'd for Chemical Formula: $C_{24}H_{19}BrCl_2FN_5O_2$: 580.01, found 579.98.

1-(3-aminophenyl)-3-cyclopropyl-5-((2-fluoro-4-iodophenyl)amino)-6,8-dimethylpyrido[4,3-*d*]pyrimidine-2,4,7(1*H*,3*H*,6*H*)-trione (**25**)[2]

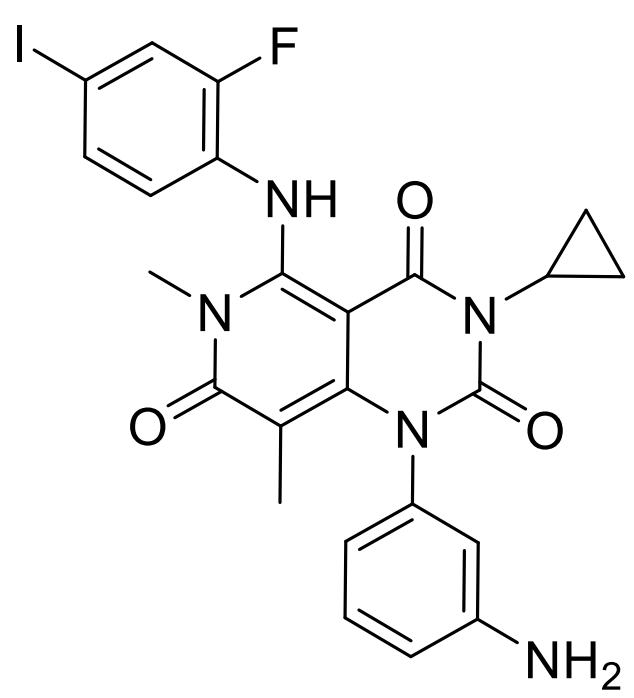

Intermediate **25** and its precursors **17-24** were prepared according to procedures described previously by Abe and co-workers.[2] MS (*m/z*) ESI $[M+1]^+$ calc'd for $C_{24}H_{21}FIN_5O_3$: 574.07, found 573.96.[2]

N-(3-(3-cyclopropyl-5-((2-fluoro-4-iodophenyl)amino)-6,8-dimethyl-2,4,7-trioxo-3,4,6,7-tetrahydropyrido[4,3-*d*]pyrimidin-1(2*H*)-yl)phenyl)acrylamide (**26a**, **TWG-07-148**)

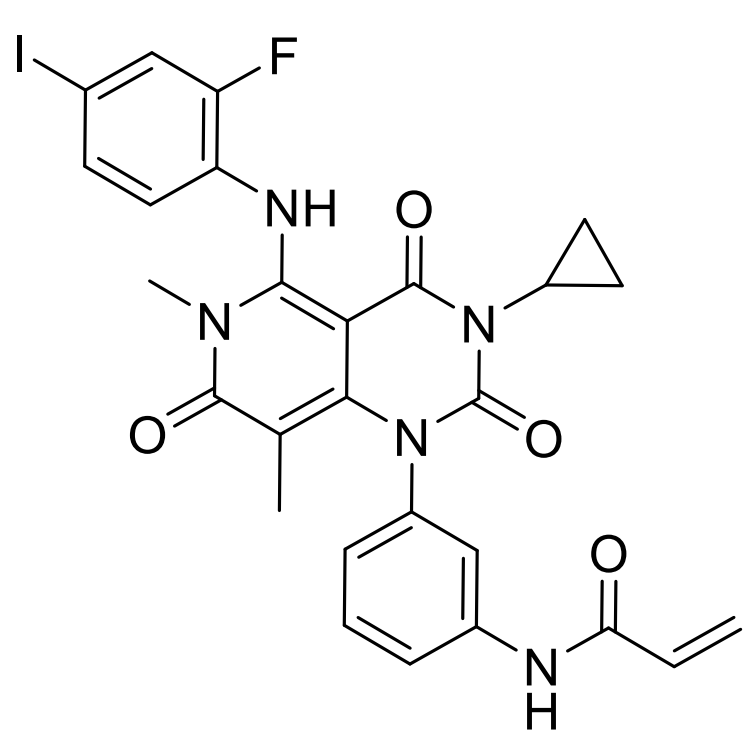

General Procedure D: A mixture of 1-(3-aminophenyl)-3-cyclopropyl-5-((2-fluoro-4-iodophenyl)amino)-6,8-dimethylpyrido[4,3-*d*]pyrimidine-2,4,7(1*H*,3*H*,6*H*)-trione (**25**, 30 mg, 0.15 mmol), HATU (0.114 g, 0.30 mmol), acrylic acid (12 mg, 0.165 mmol), DIEA (78 µL, 0.45 mmol), and DMF (0.5 mL) was stirred at RT for 0.5 h. The crude mixture was purified by reverse phase-HPLC, 0–80% ACN/H_2O (0.035% TFA modifier) over 30 minutes. Like fractions containing product were pooled and lyophilized to give the title compound (14 mg, 15%). ^{1}H NMR (500 MHz, DMSO) δ 11.08 (s, 1H), 10.32 (s, 1H), 7.79 (dd, J = 10.3, 1.8 Hz, 1H), 7.70 (m, 2H), 7.56 (dd, J = 8.4, 1.2 Hz, 1H), 7.41 (t, J = 8.3 Hz, 1H), 7.11 – 7.06 (m, 1H), 6.93 (t, J = 8.6 Hz, 1H), 6.44 (dd, J = 17.0, 10.1 Hz, 1H), 6.27 (dd, J = 17.0, 1.9 Hz, 1H), 5.78 (dd, J = 10.1, 1.9 Hz, 1H), 3.08 (s, 3H), 2.67 – 2.60 (m, 1H), 1.27 (s, 3H), 0.99 – 0.93 (m, 2H), 0.71 – 0.64 (m, 2H). ^{13}C NMR (125 MHz, DMSO) δ 169.0, 165.9, 164.3, 160.8, 155.6, 150.1, 142.29, 140.8, 136.5, 132.0, 129.8, 128.6, 126.5, 125.9, 124.6, 123.2, 123.1, 122.8, 121.4, 98.5, 92.6, 70.8, 31.0, 28.1, 11.3, 9.7 (2C). MS (*m/z*) ESI [M+1]$^+$ calc'd for $C_{27}H_{23}FIN_5O_4$: 628.09, found 627.79.

<u>2-chloro-*N*-(3-(3-cyclopropyl-5-((2-fluoro-4-iodophenyl)amino)-6,8-dimethyl-2,4,7-trioxo-3,4,6,7-tetrahydropyrido[4,3-*d*]pyrimidin-1(2*H*)-yl)phenyl)acetamide</u> (**26b**, **TWG-07-154**)

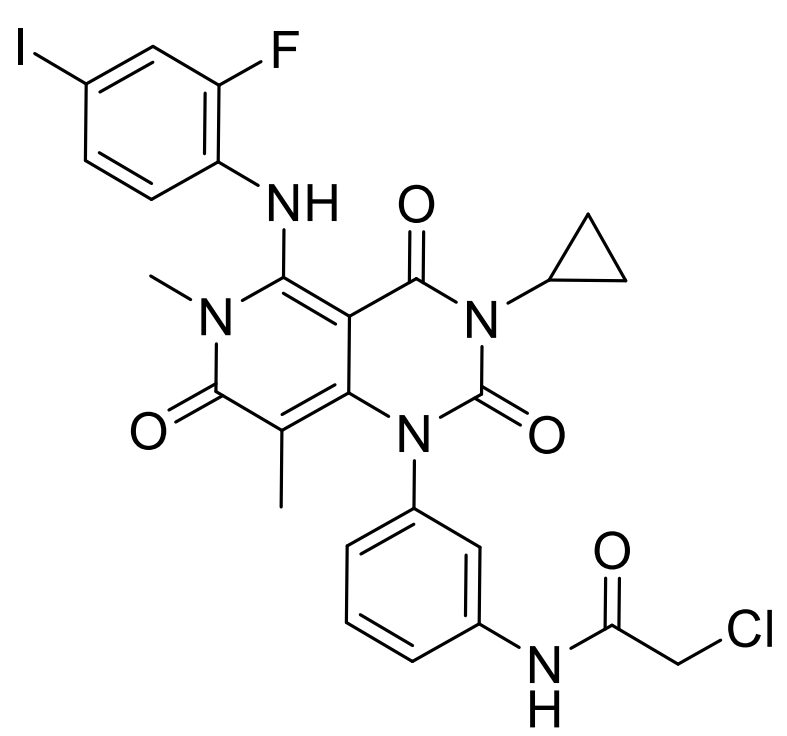

Compound **26b** (**TWG-07-154**) was prepared as described for **26a** (**TWG-07-148**), following **<u>General Procedure D</u>**, substituting acrylic acid by chloroacetic acid. The crude was purified by reverse phase-HPLC, 0–80% ACN/H_2O (0.035% TFA modifier) over 30 minutes to give the title compound (21 mg, 20%). ^{1}H NMR (500 MHz, DMSO) δ 11.08 (s, 1H), 10.48 (s, 1H), 7.79 (dd, J = 10.3, 1.9 Hz, 1H), 7.63 (d, J = 0.9 Hz, 1H), 7.61 (s, 1H), 7.56 (dd, J = 8.4, 1.4 Hz, 1H), 7.42 (t, J = 8.2 Hz, 1H), 7.13 – 7.08 (m, 1H), 6.93 (t, J = 8.6 Hz, 1H), 4.27 (s, 2H), 3.08 (s, 3H), 2.66 – 2.59 (m, 1H), 1.26 (s, 3H), 0.99 – 0.92 (m, 2H), 0.70 – 0.65 (m, 2H). MS (*m/z*) ESI [M+1]$^+$ calc'd for $C_{26}H_{22}ClFIN_5O_4$: 650.05, found 649.72.

<u>*N*-(3-(3-cyclopropyl-5-((2-fluoro-4-iodophenyl)amino)-6,8-dimethyl-2,4,7-trioxo-3,4,6,7-tetrahydropyrido[4,3-*d*]pyrimidin-1(2*H*)-yl)phenyl)-2-fluoroacrylamide</u> (**17c**, **TWG-07-178**)

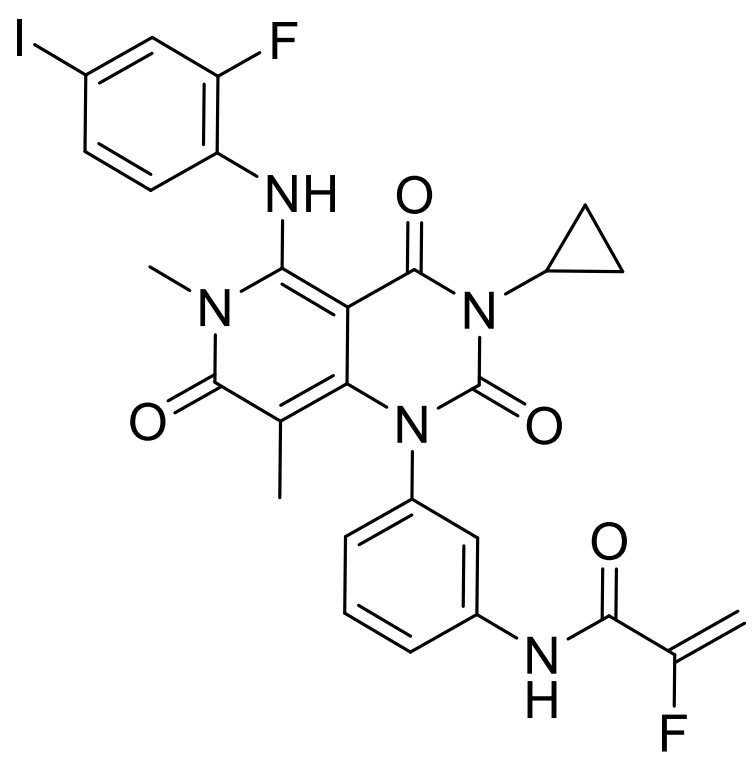

Compound **26c** (**TWG-07-178**) was prepared as described for **26a** (**TWG-07-148**), following **General Procedure D**, substituting acrylic acid by 2-fluoroacrylic acid. The crude was purified by reverse phase-HPLC, 0–80% ACN/H_2O (0.035% TFA modifier) over 30 minutes to give the title compound (14 mg, 13%). ^1H NMR (500 MHz, DMSO) δ 11.07 (s, 1H), 10.46 (s, 1H), 7.80 (dd, J = 10.3, 1.9 Hz, 1H), 7.77 – 7.73 (m, 2H), 7.56 (dd, J = 8.4, 1.3 Hz, 1H), 7.43 (t, J = 8.6 Hz, 1H), 7.16 (ddd, J = 8.0, 1.8, 1.0 Hz, 1H), 6.93 (t, J = 8.6 Hz, 1H), 5.73 and 5.68 (sum of two doublets, J = 3.7 Hz, 1H), 5.47 and 5.43 (sum of two doublets, J = 3.7 Hz, 1H), 3.09 (s, 3H), 2.66 – 2.60 (m, 1H), 1.26 (s, 3H), 0.99 – 0.92 (m, 2H), 0.72 – 0.63 (m, 2H). MS (*m/z*) ESI $[M+1]^+$ calc'd for $C_{27}H_{22}F_2IN_5O_4$: 646.08, found 646.06.

^{1}H / ^{13}C NMR SPECTRA

^{1}H NMR spectra for compound GD-01-012

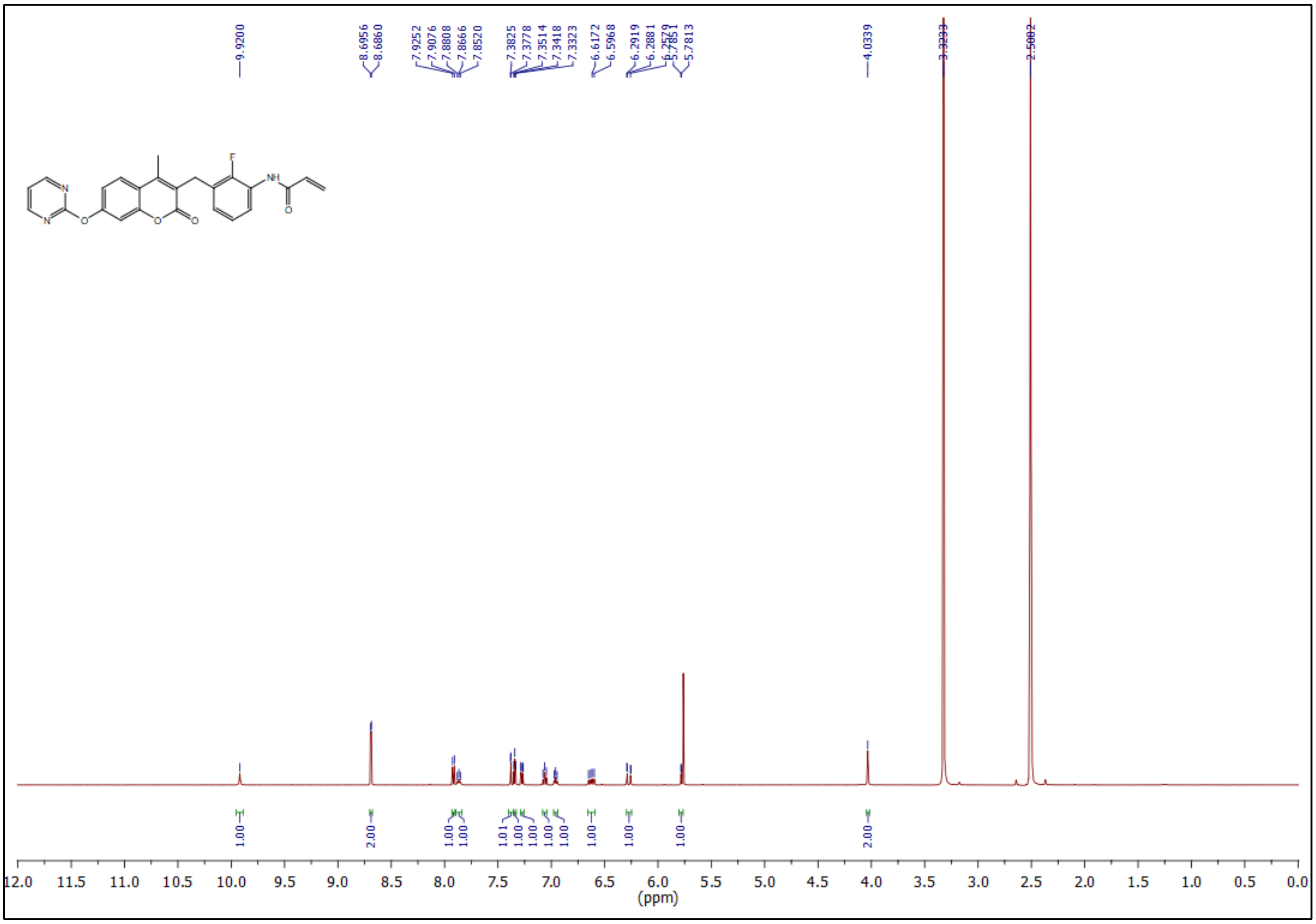

^{13}C NMR spectra for compound GD-01-012

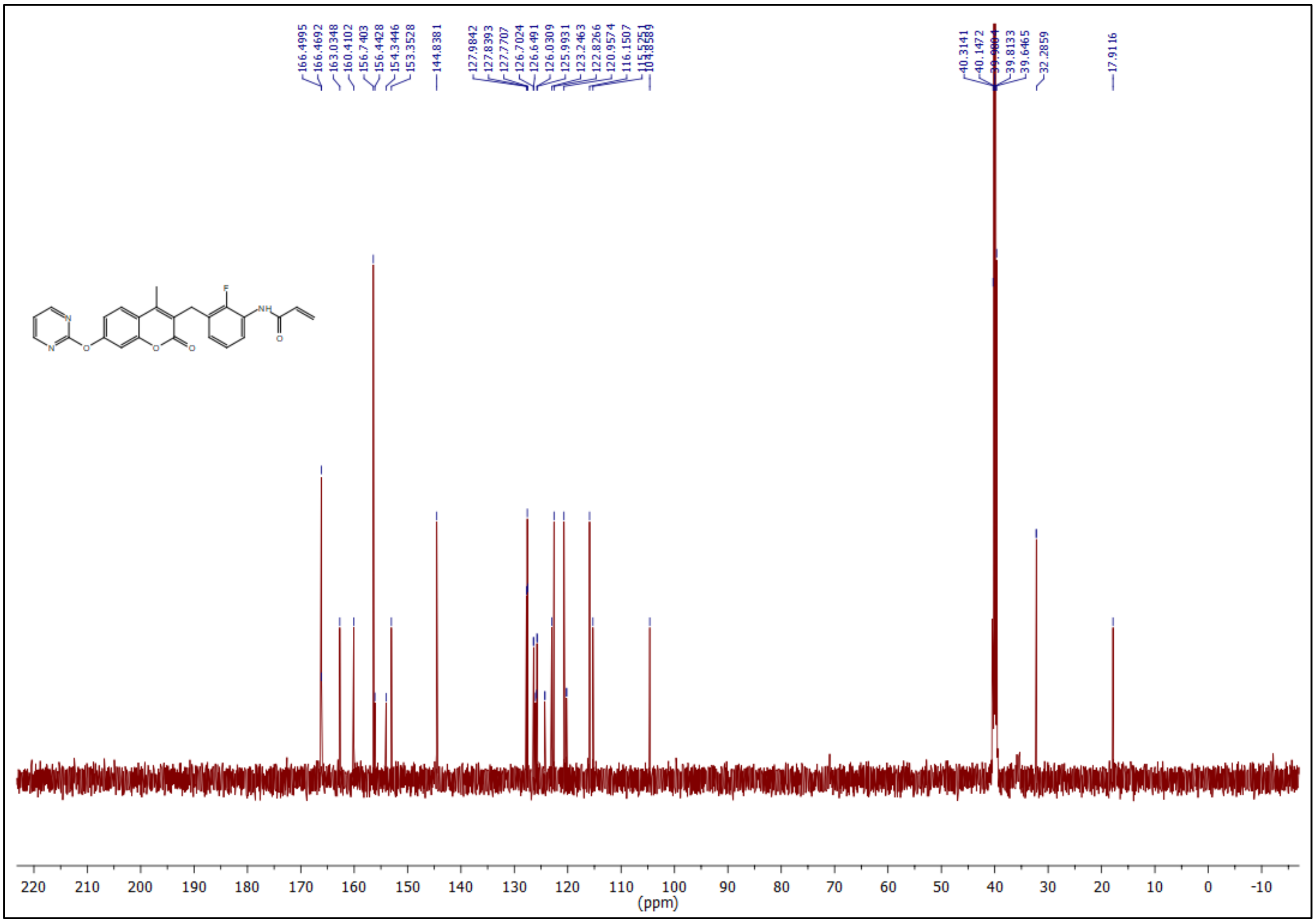

^{1}H NMR spectra for compound GD-01-014

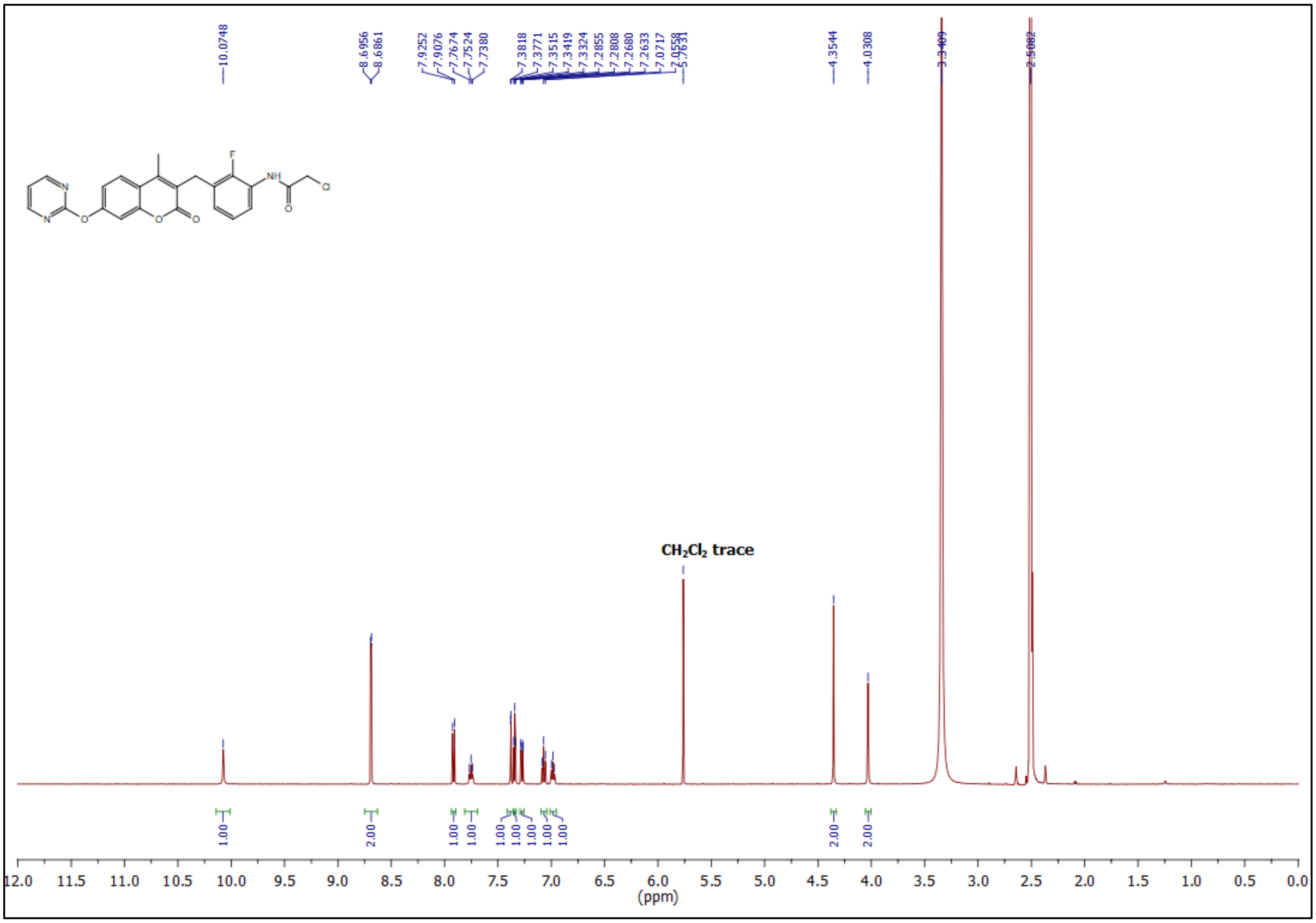

^{1}H NMR spectra for compound GD-01-022

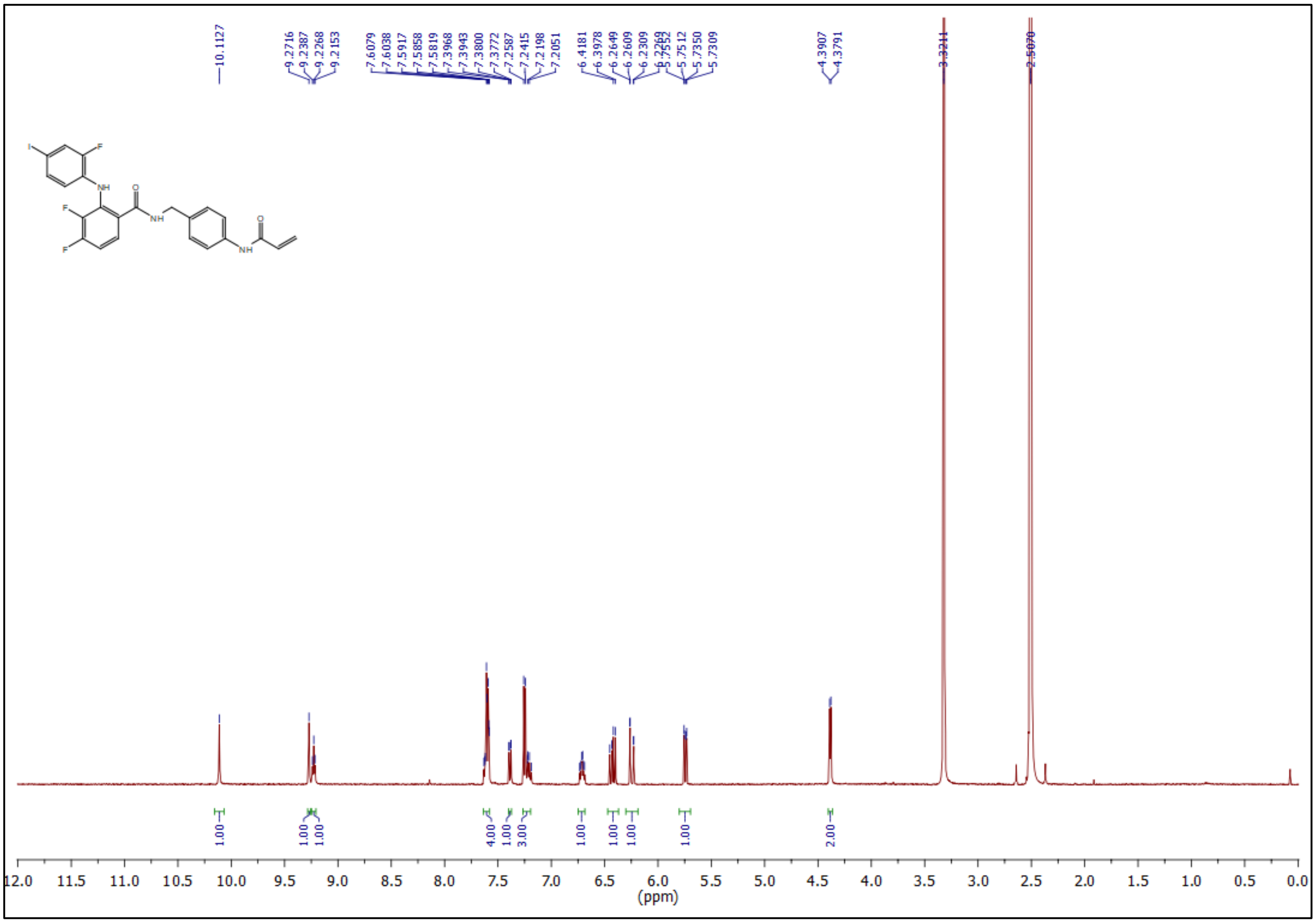

^{1}H NMR spectra for compound GD-01-024

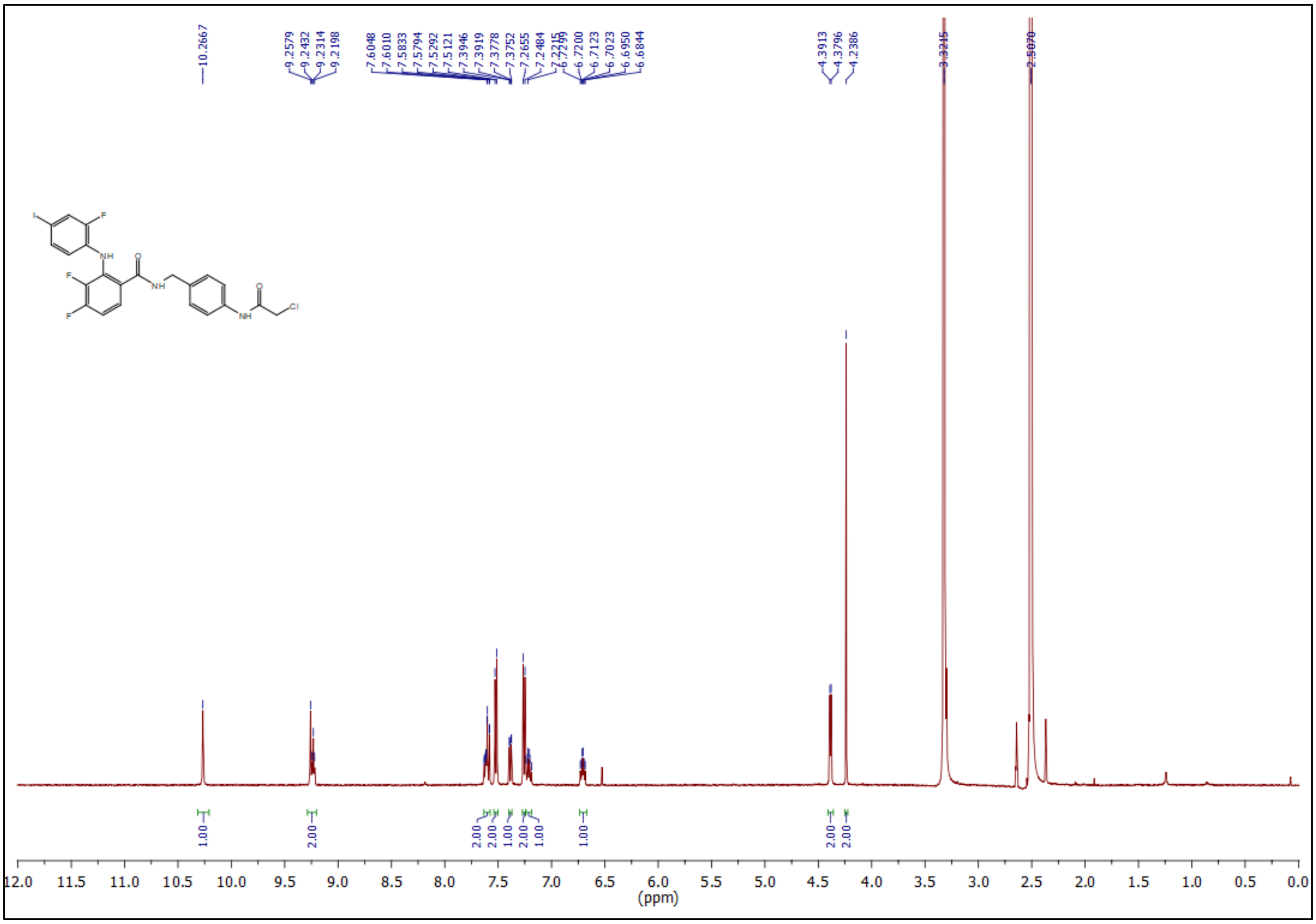

^{1}H NMR spectra for compound GD-01-036

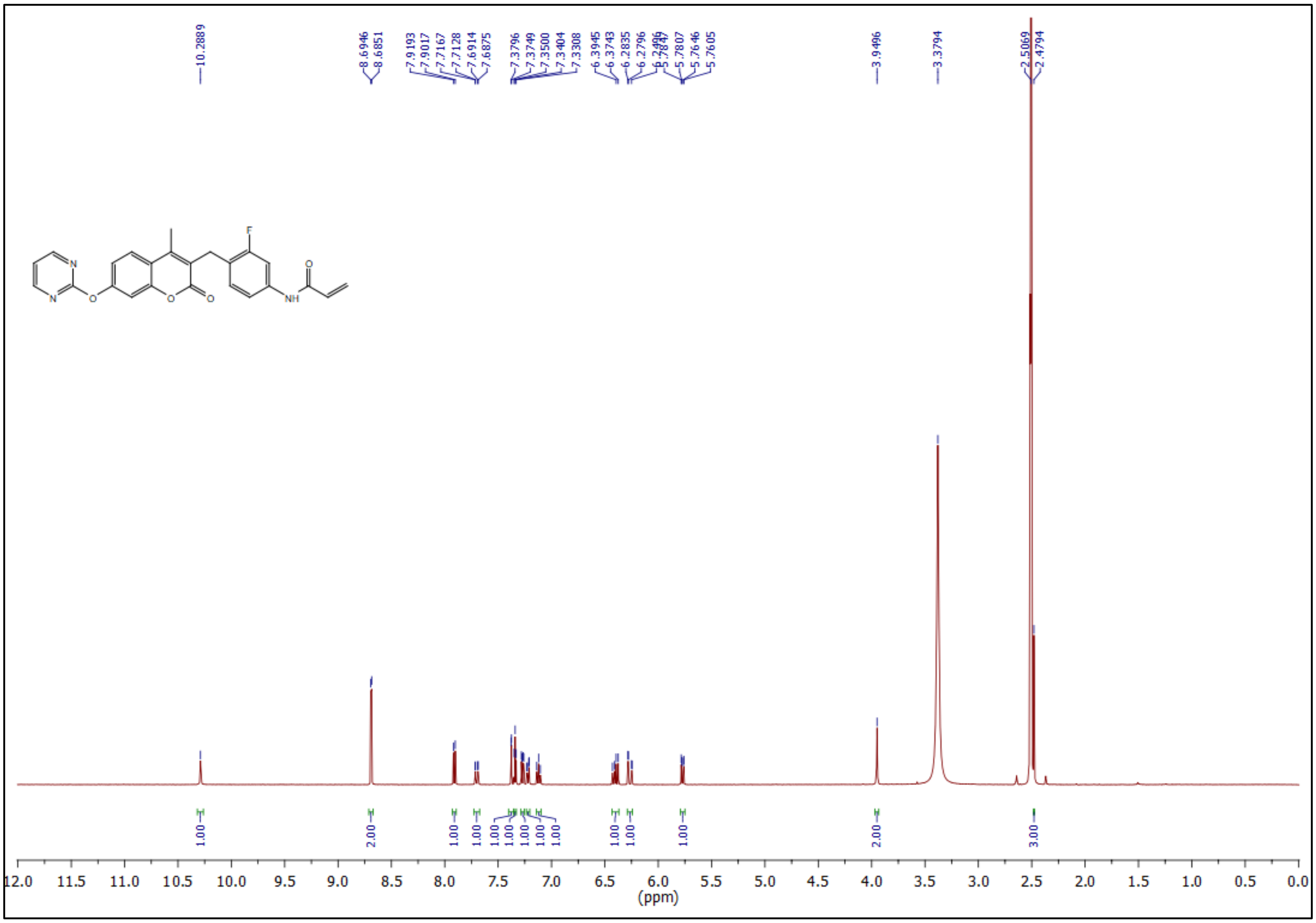

^{1}H NMR spectra for compound GD-01-038

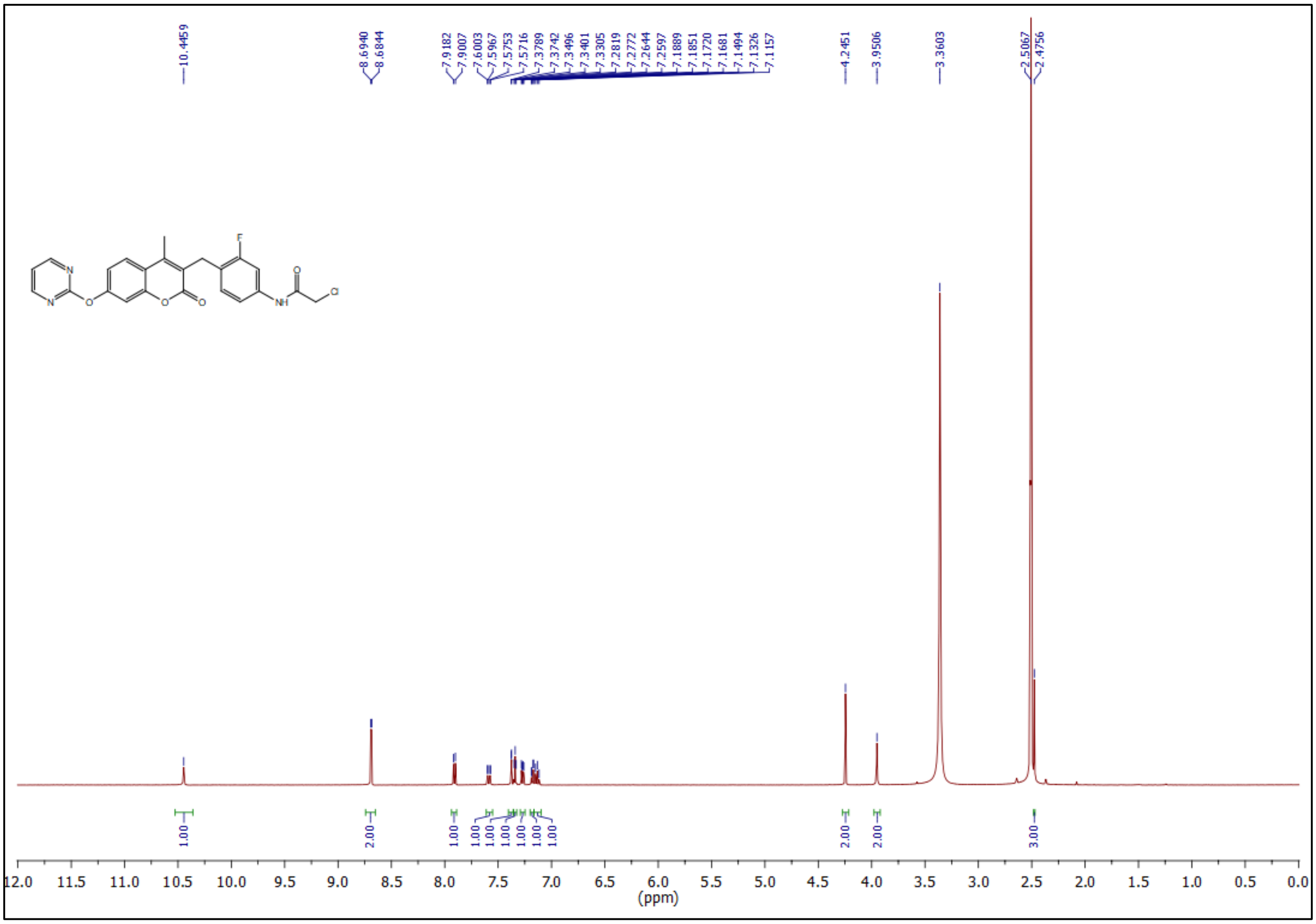

^{1}H NMR spectra for compound GD-01-066

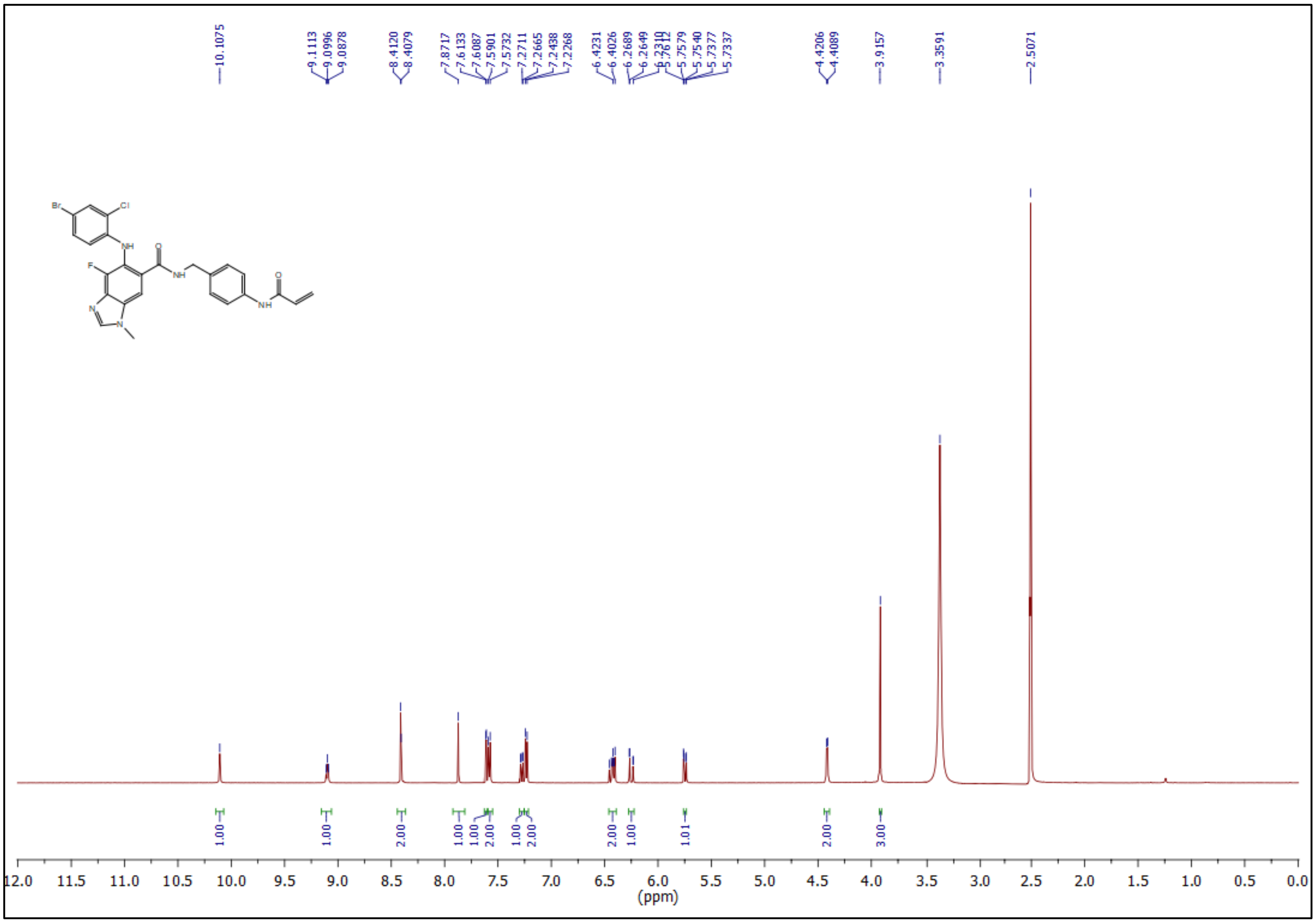

^{1}H NMR spectra for compound GD-01-068

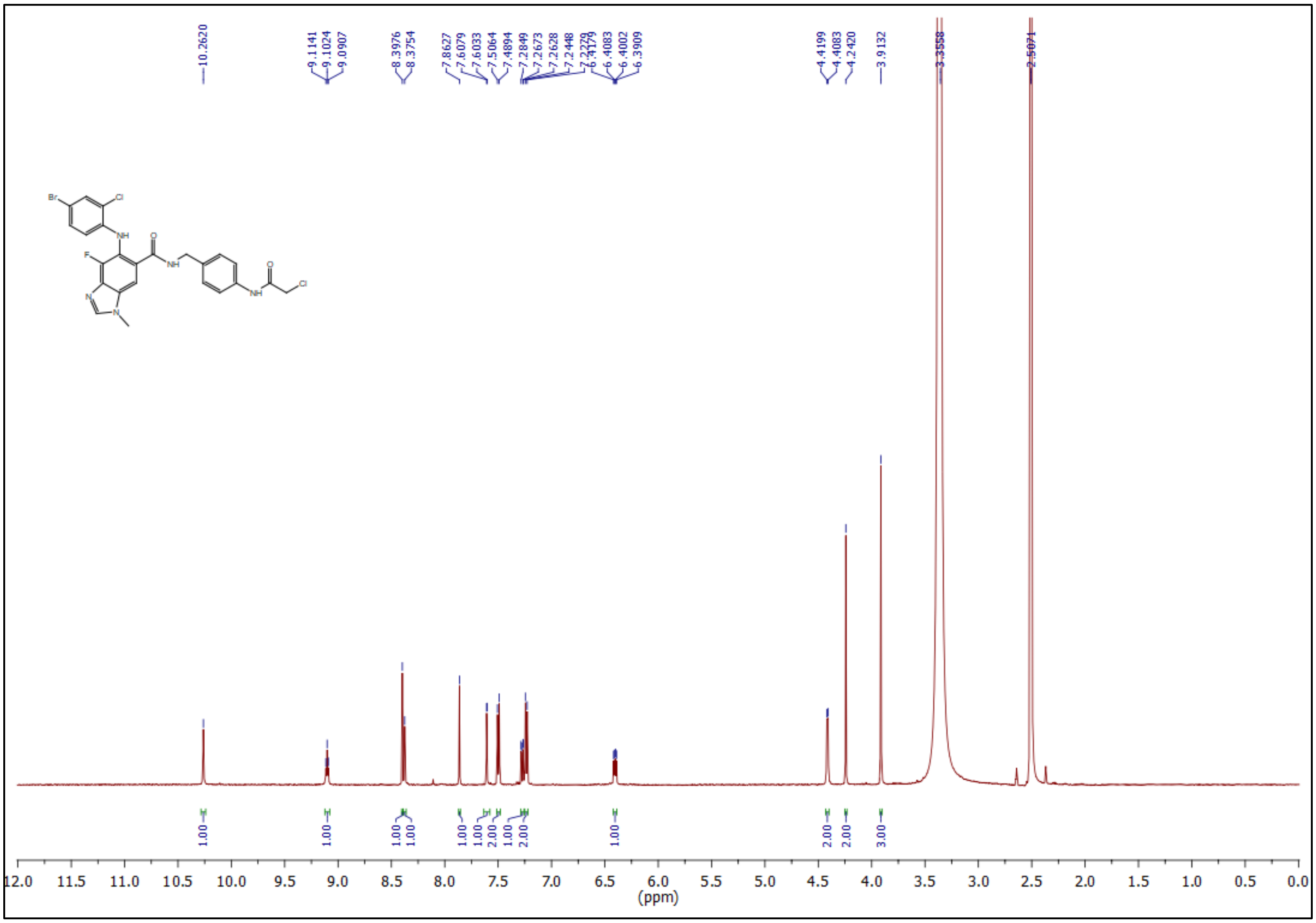

^{1}H NMR spectra for compound TWG-07-148

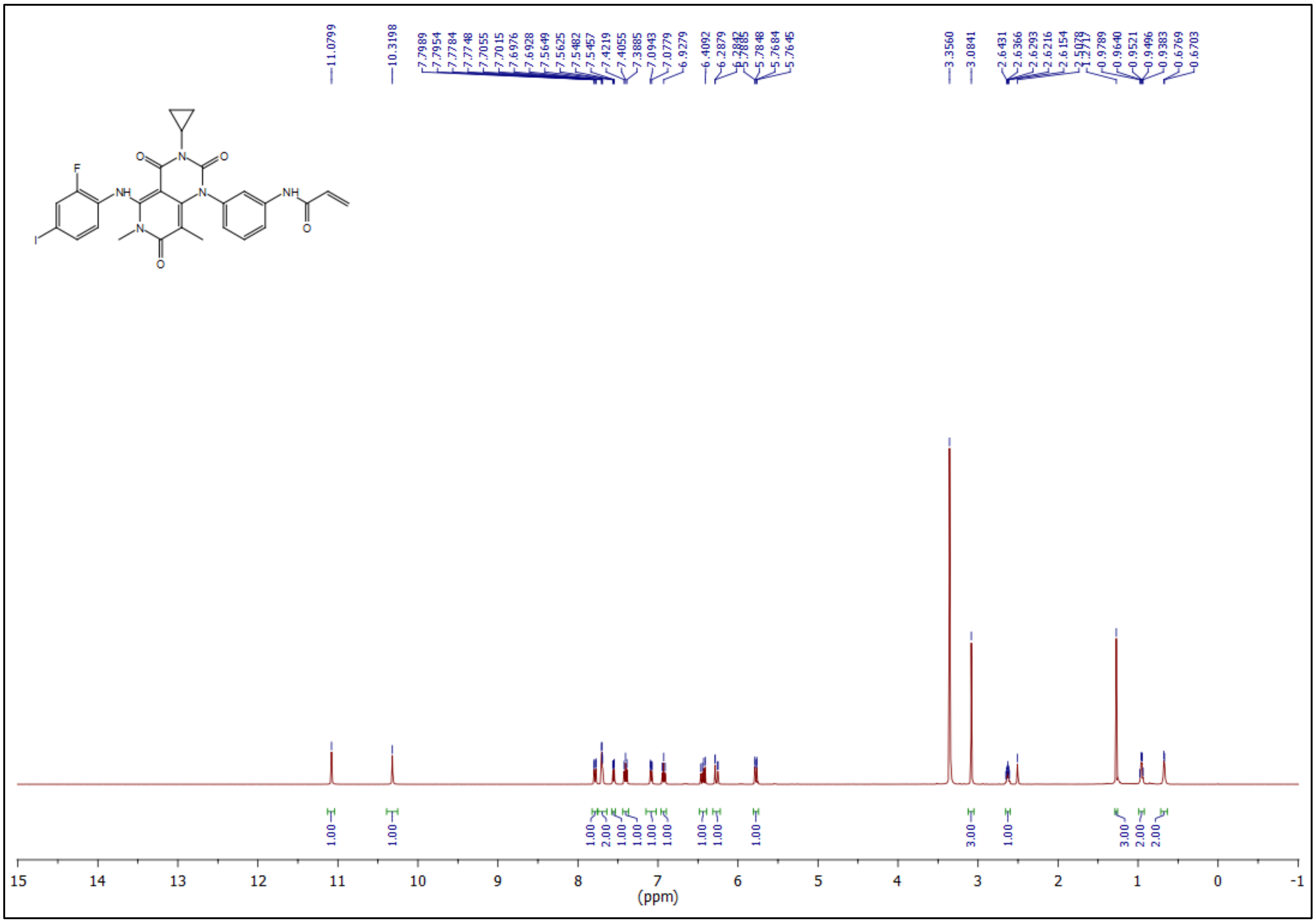

^{13}C NMR spectra for compound TWG-07-148

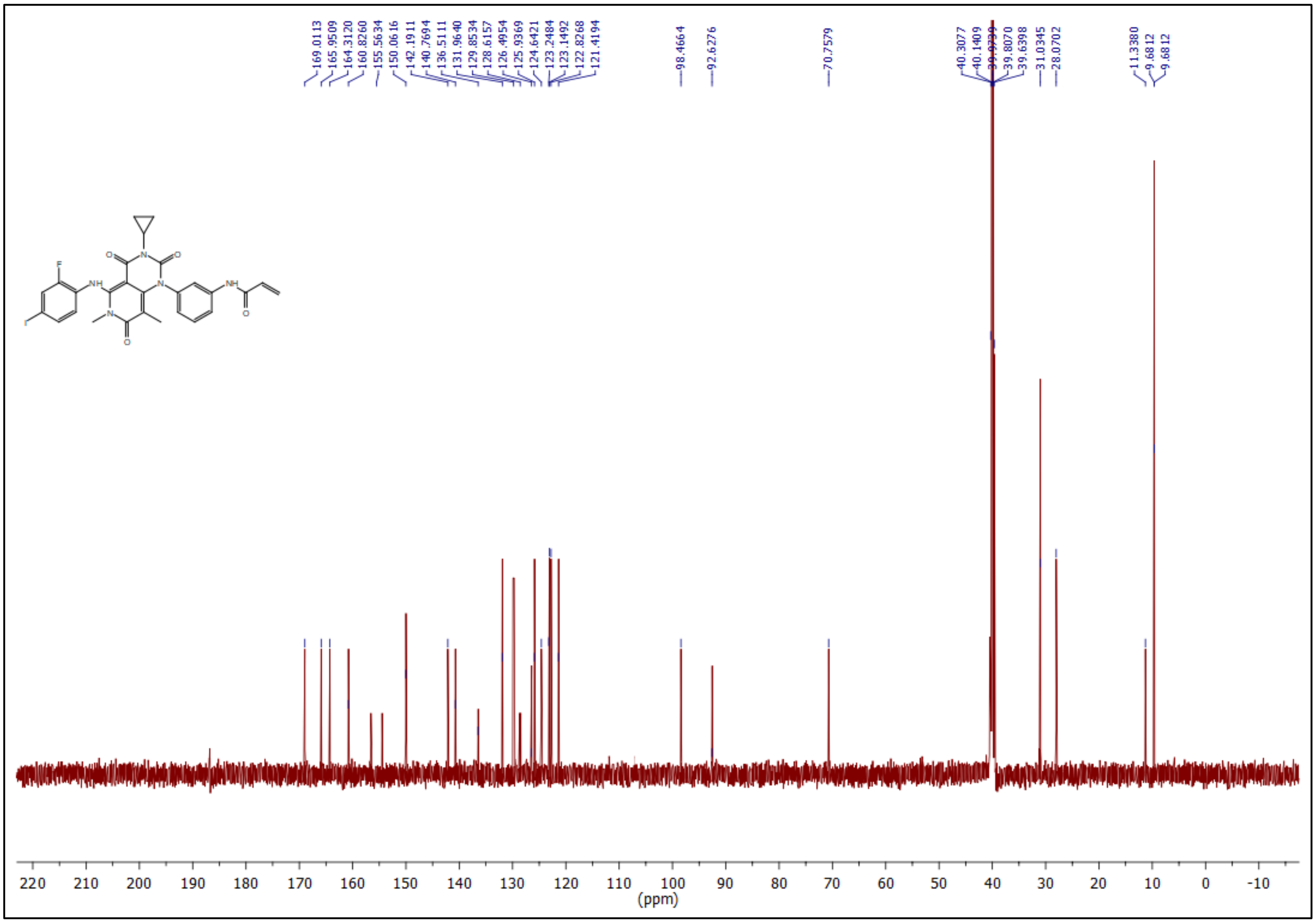

^{1}H NMR spectra for compound TWG-07-154

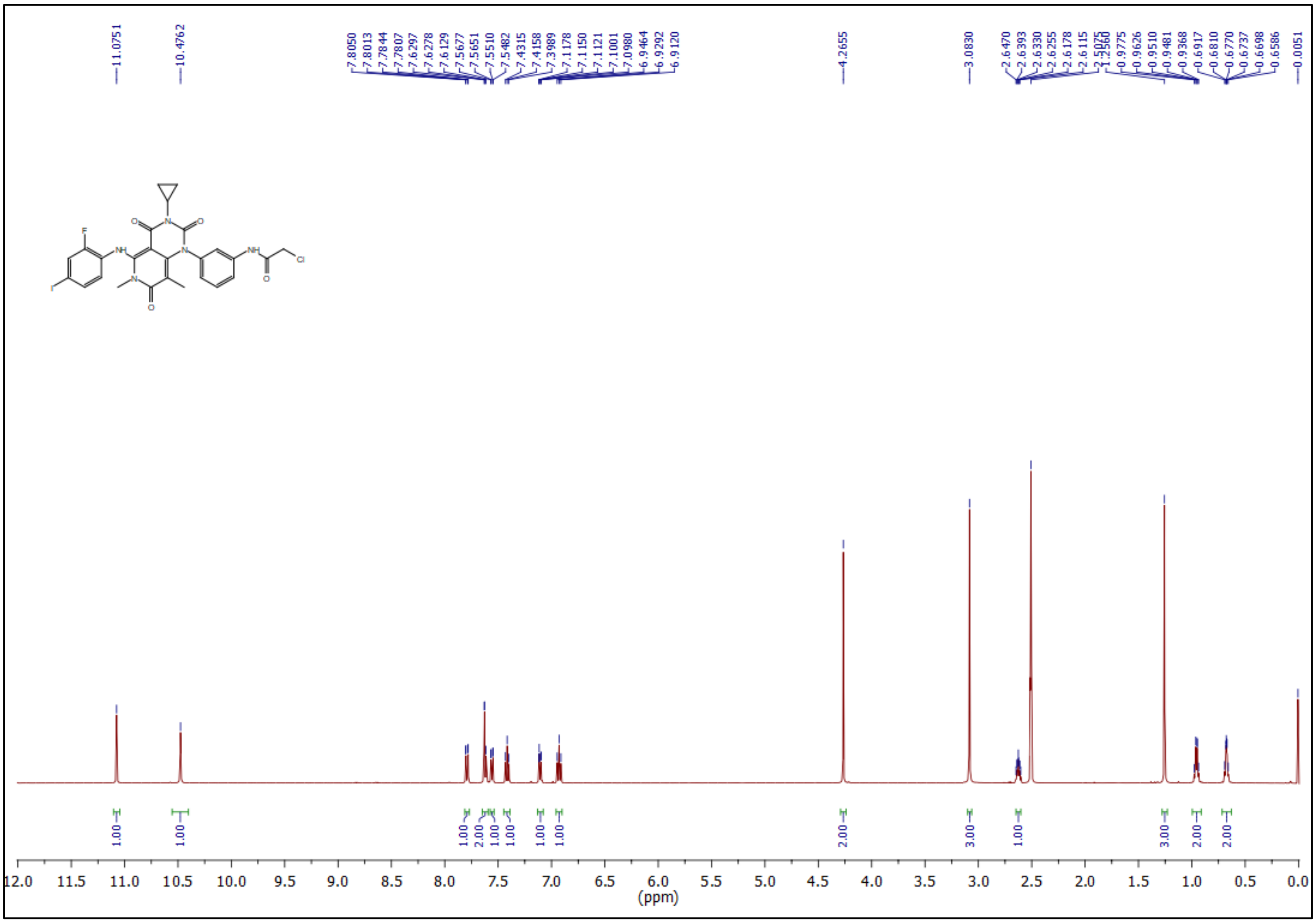

^{1}H NMR spectra for compound TWG-07-178

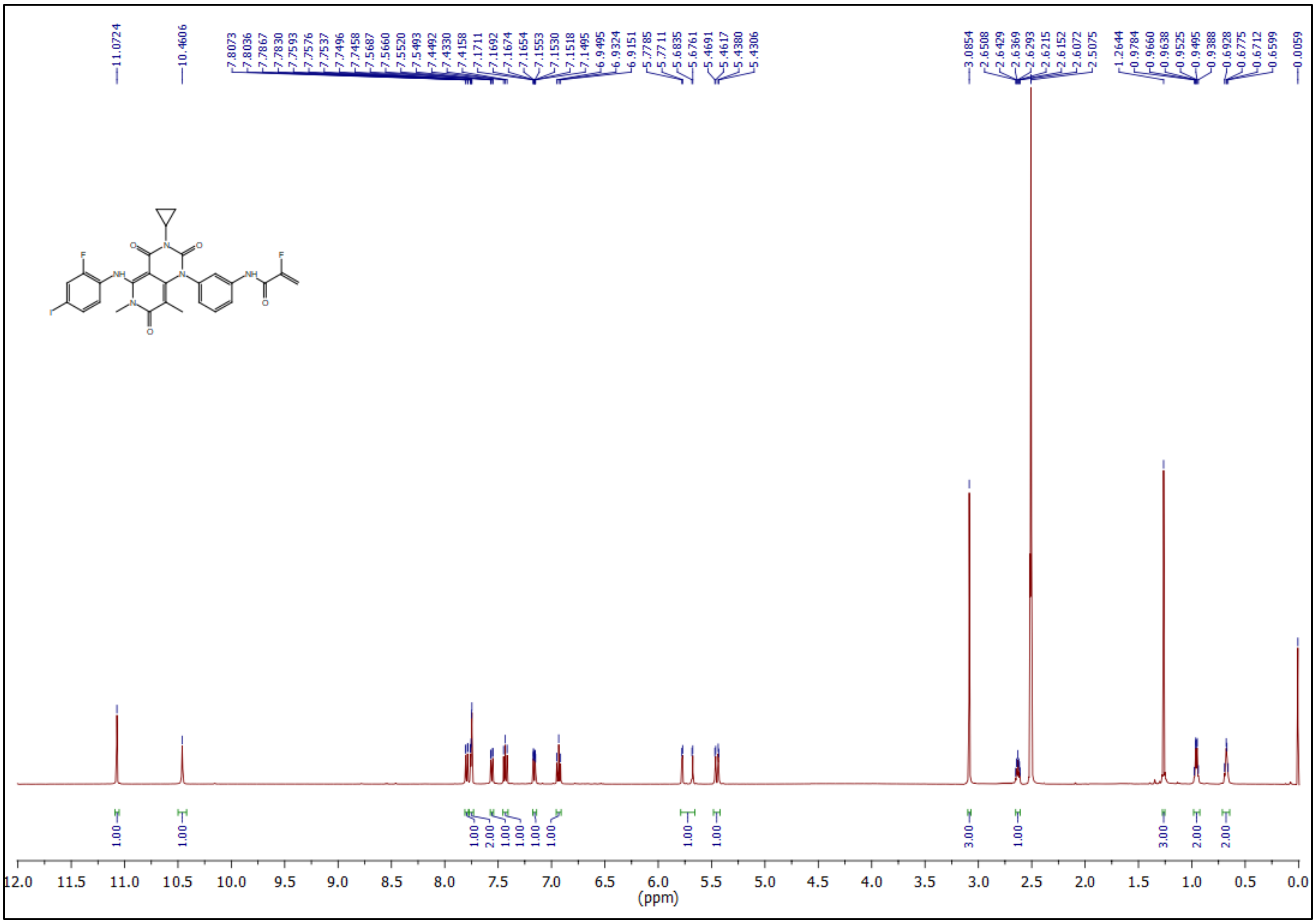

LCMS SPECTRA ANALYSIS

Compound GD-01-012

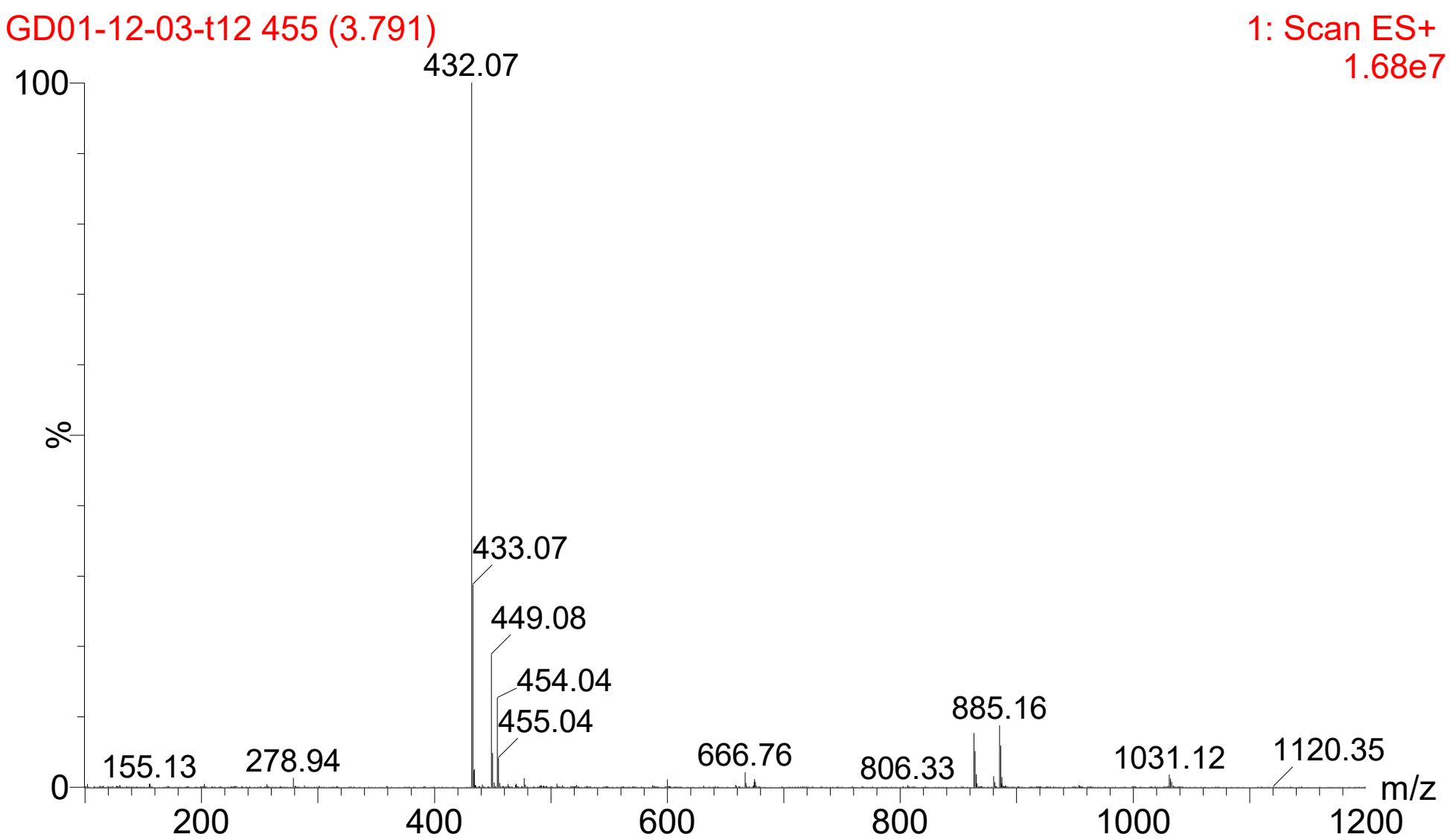

Compound GD-01-014

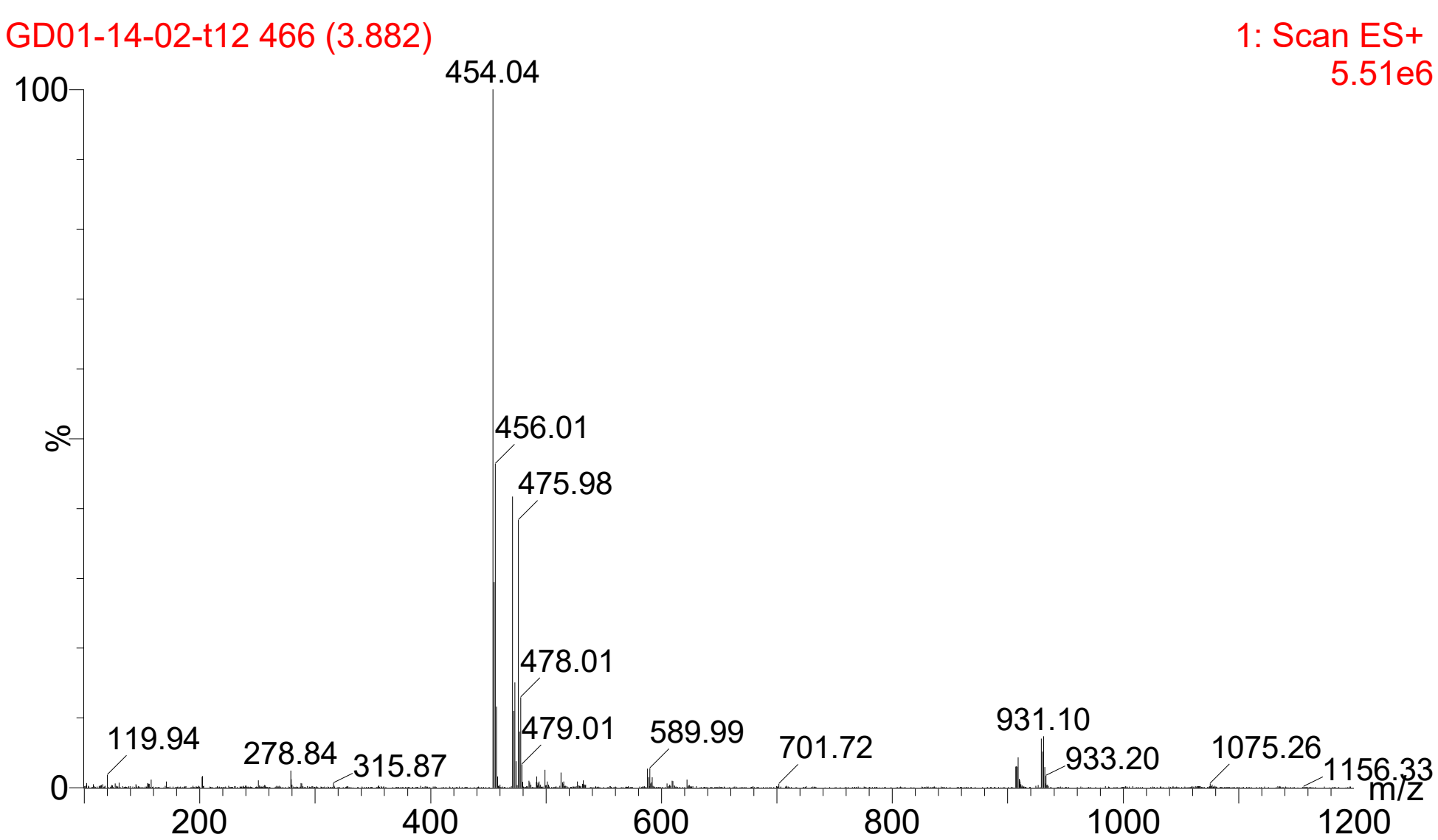

Compound GD-01-022

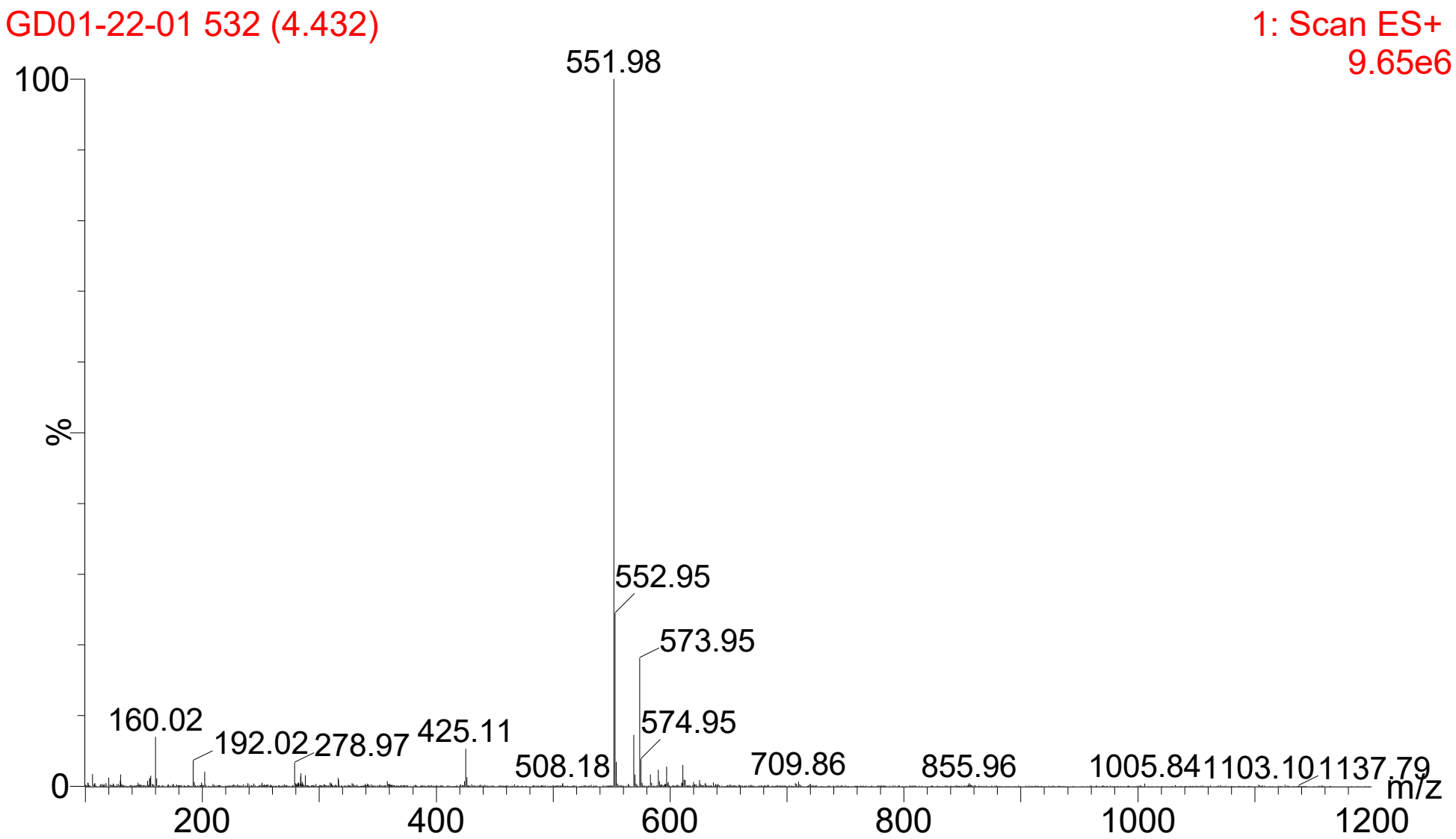

Compound GD-01-024

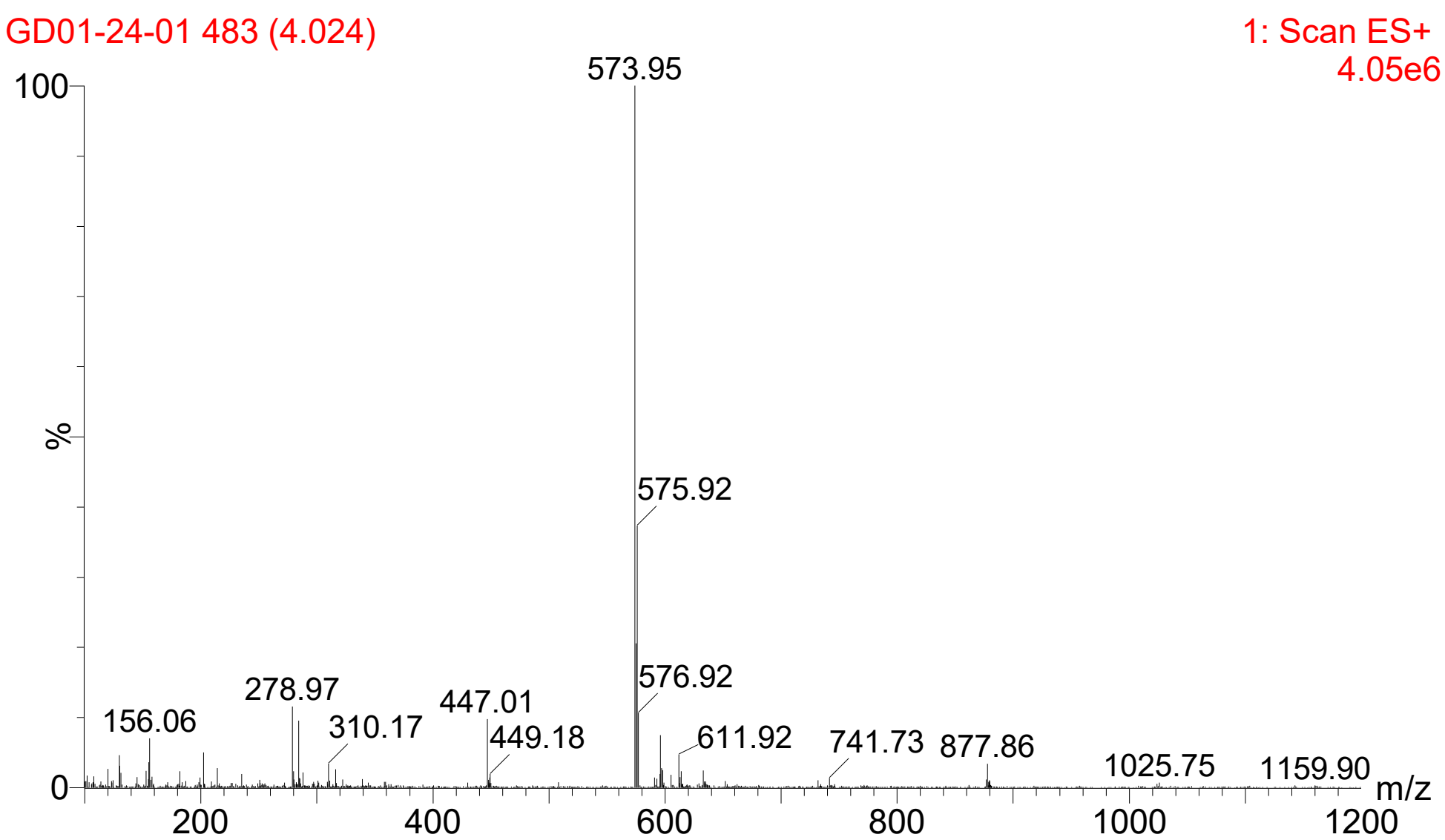

Compound GD-01-036

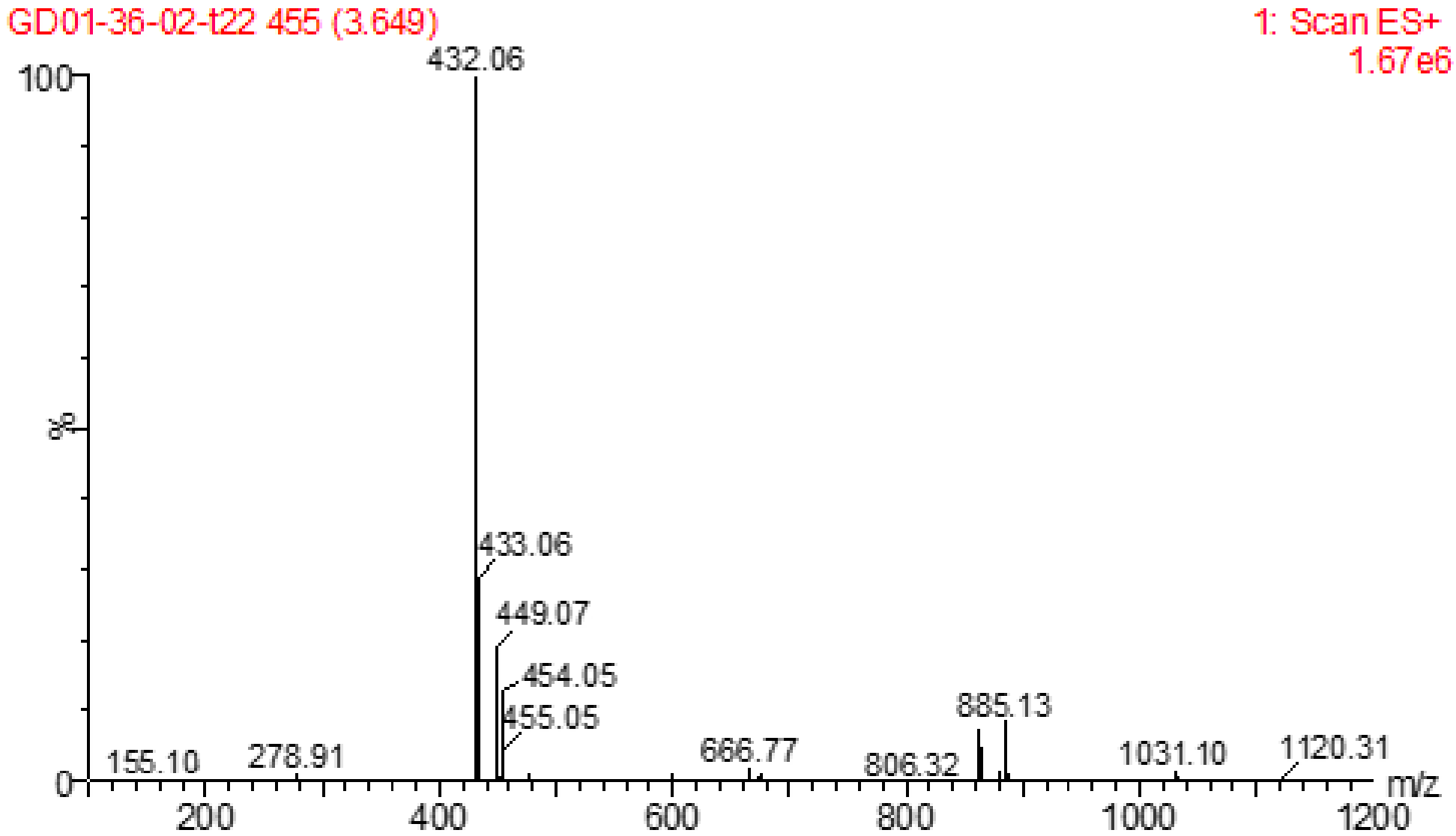

Compound GD-01-038

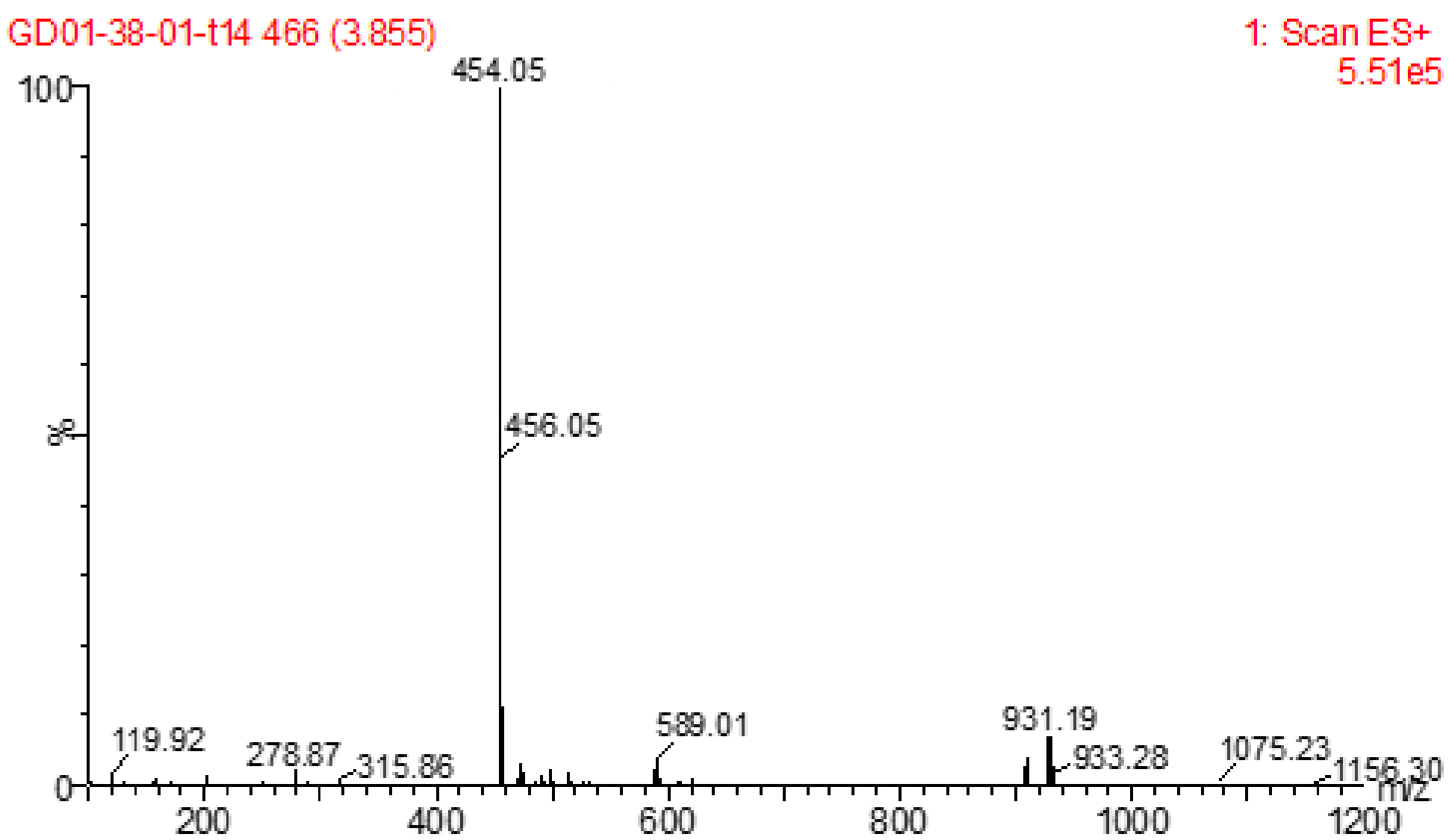

Compound GD-01-066

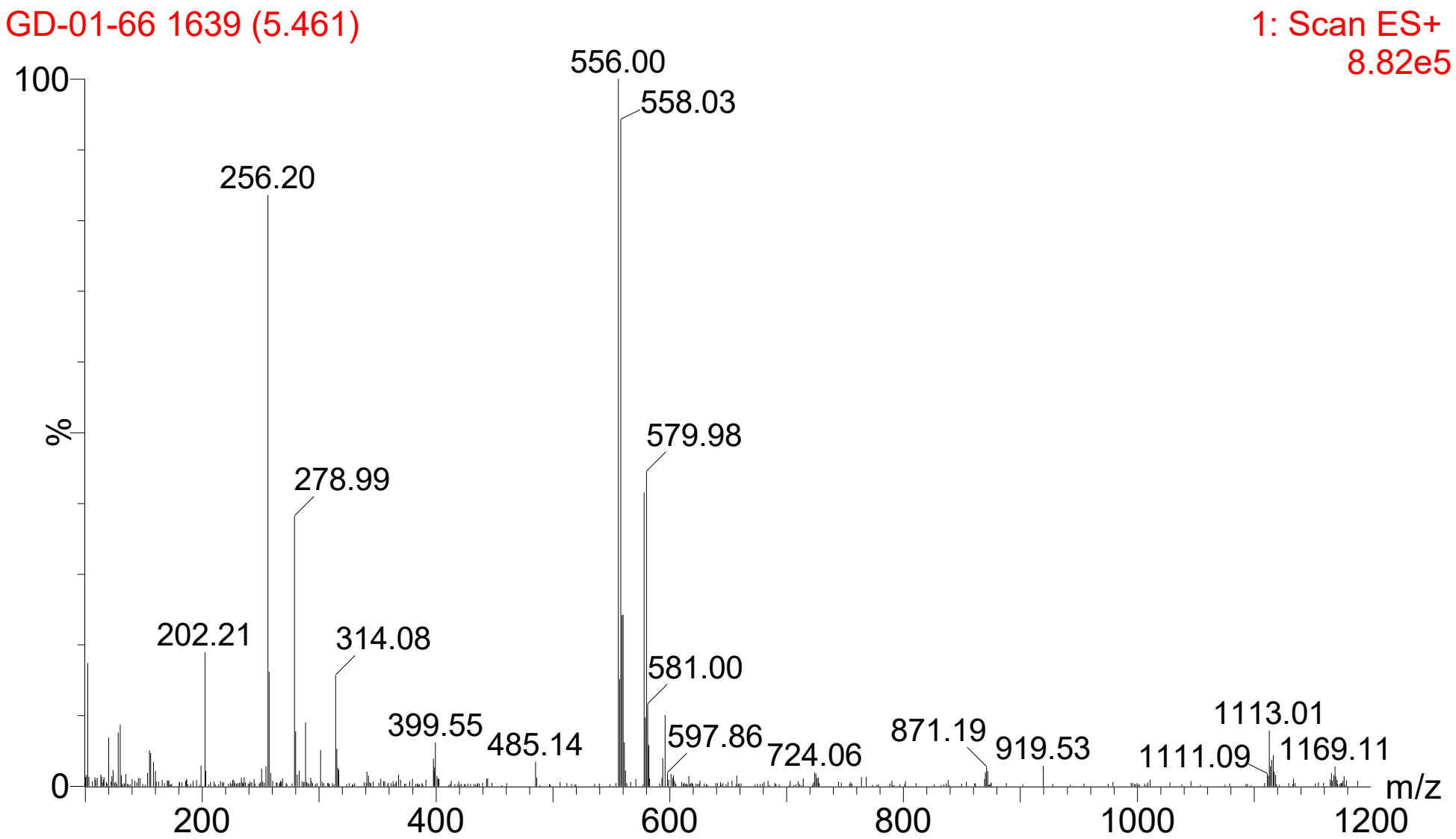

Compound GD-01-068

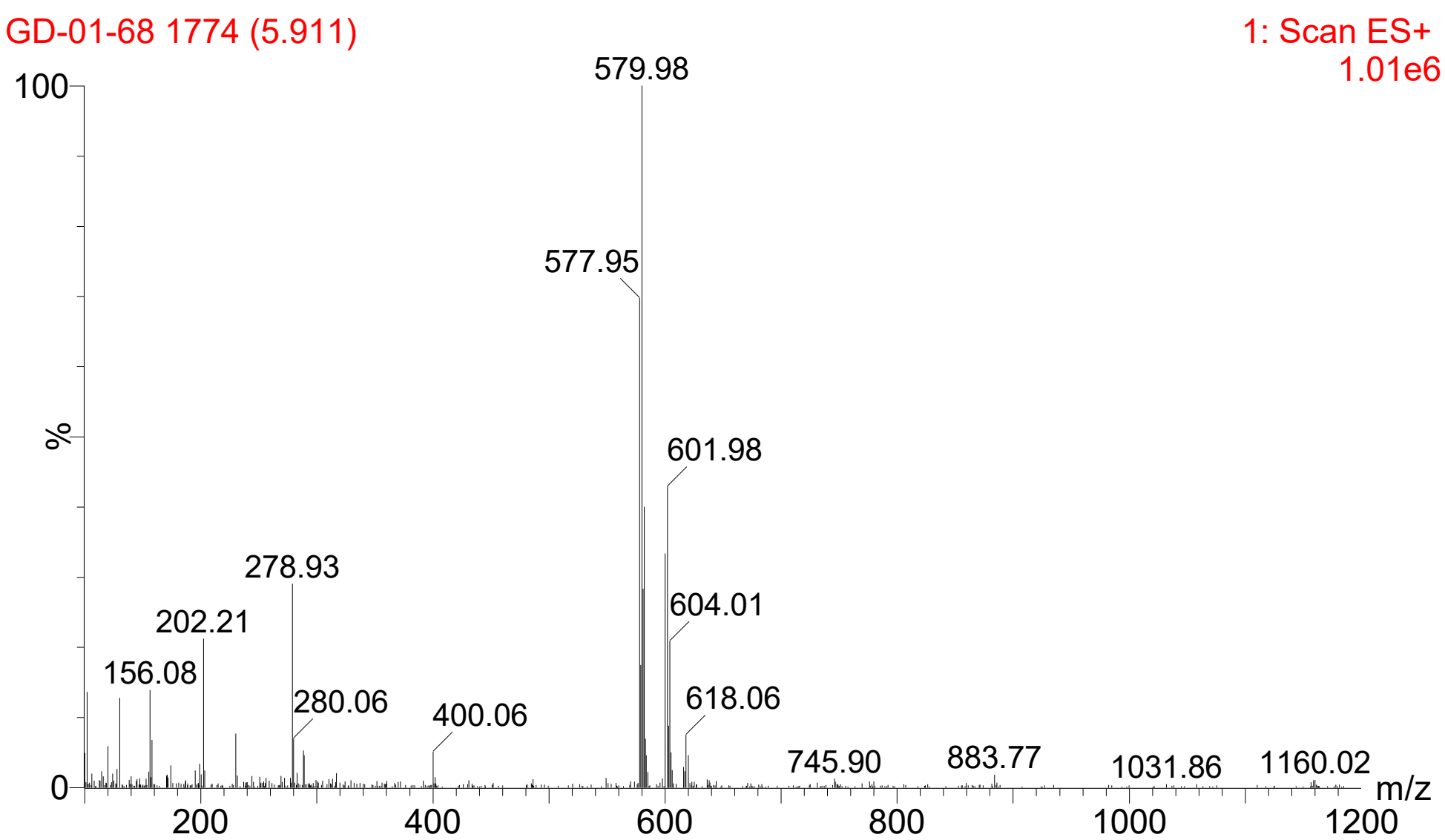

Compound TWG-07-148

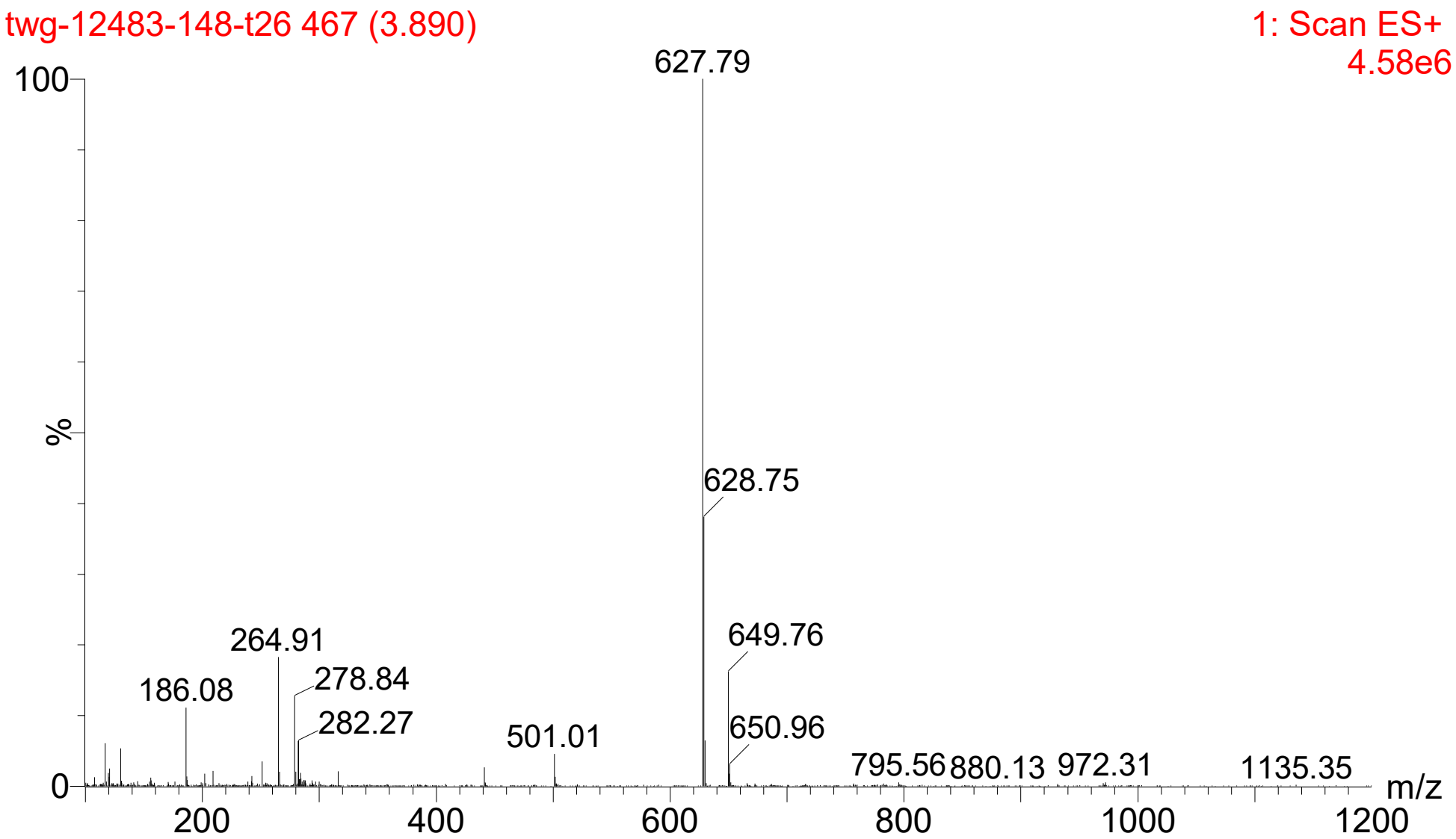

Compound TWG-07-154

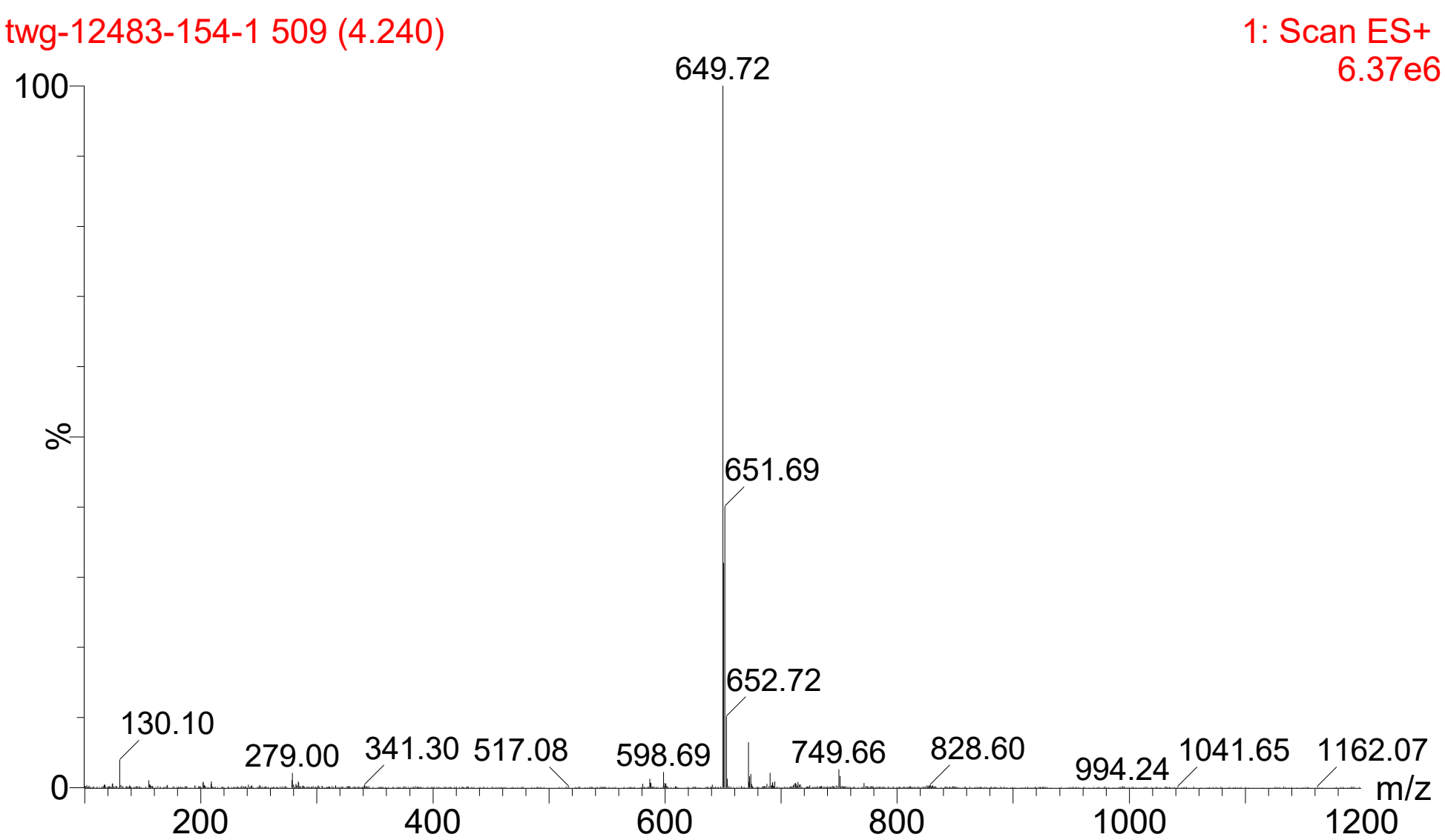

Compound TWG-07-178

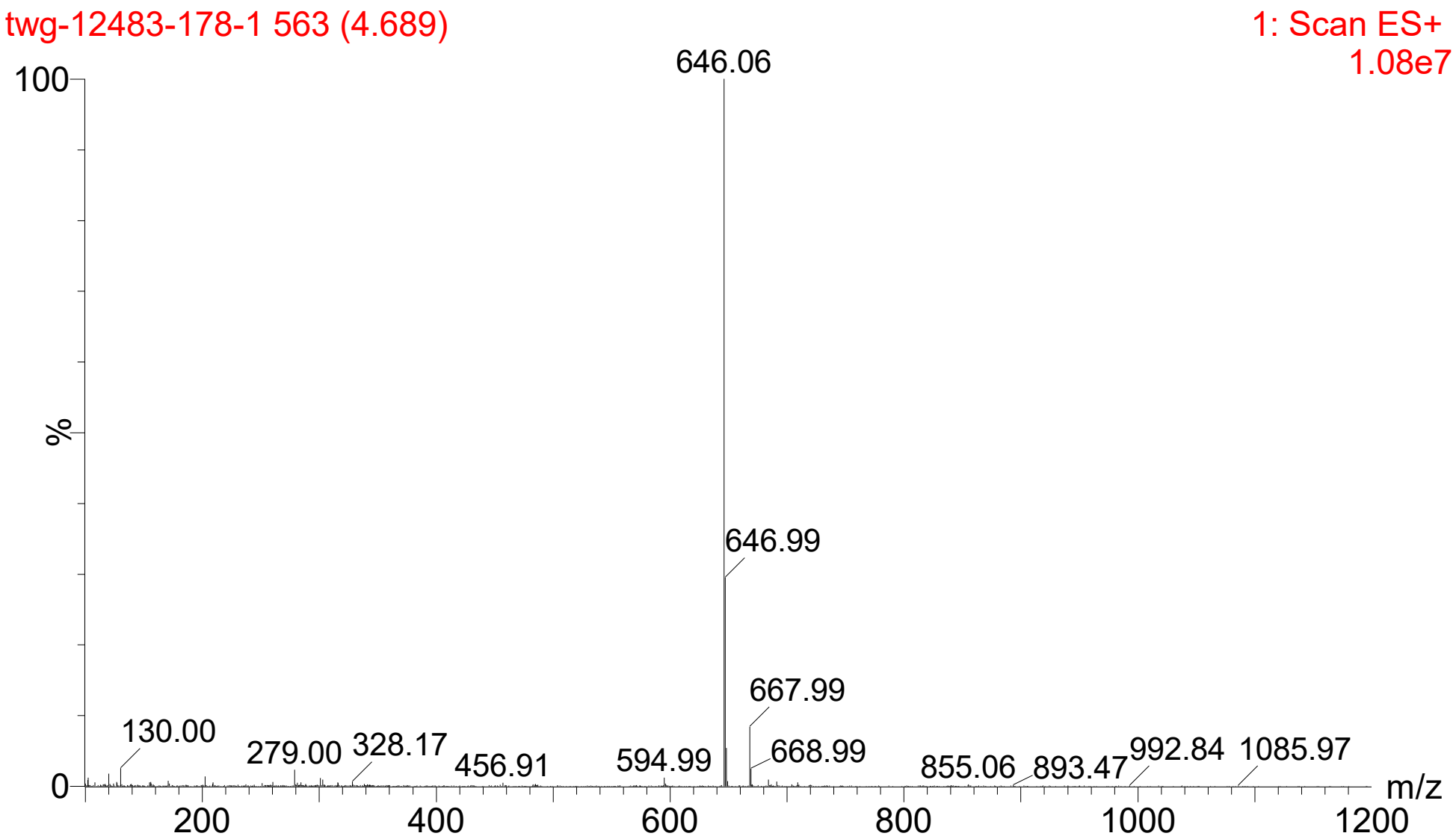

Movie S1: 3D variability analysis of the cryo-EM reconstruction showing a rocking motion of MEK1
